# Wrist morphology reveals substantial locomotor diversity among early catarrhines: an analysis of capitates from the early Miocene of Tinderet (Kenya)

**DOI:** 10.1038/s41598-019-39800-3

**Published:** 2019-03-06

**Authors:** Craig Wuthrich, Laura M. MacLatchy, Isaiah O. Nengo

**Affiliations:** 1 Department of Anthropology, University of Michigan, Ann Arbor, MI 48109 USA; 2 Department of Evolutionary Anthropology, Duke University, Durham, NC 27708 USA; 3 Turkana Basin Institute, Stony Brook University, Stony Brook, NY 11794 USA; 4 Turkana University College, P.O. Box 69-30500, Lodwar, Kenya

## Abstract

Considerable taxonomic diversity has been recognised among early Miocene catarrhines (apes, Old World monkeys, and their extinct relatives). However, locomotor diversity within this group has eluded characterization, bolstering a narrative that nearly all early catarrhines shared a primitive locomotor repertoire resembling that of the well-described arboreal quadruped *Ekembo heseloni*. Here we describe and analyse seven catarrhine capitates from the Tinderet Miocene sequence of Kenya, dated to ~20 Ma. 3D morphometrics derived from these specimens and a sample of extant and fossil capitates are subjected to a series of multivariate comparisons, with results suggesting a variety of locomotor repertoires were present in this early Miocene setting. One of the fossil specimens is uniquely derived among early and middle Miocene capitates, representing the earliest known instance of great ape-like wrist morphology and supporting the presence of a behaviourally advanced ape at Songhor. We suggest *Rangwapithecus* as this catarrhine’s identity, and posit expression of derived, ape-like features as a criterion for distinguishing this taxon from *Proconsul africanus*. We also introduce a procedure for quantitative estimation of locomotor diversity and find the Tinderet sample to equal or exceed large extant catarrhine groups in this metric, demonstrating greater functional diversity among early catarrhines than previously recognised.

## Introduction

While catarrhines (the clade including Old World monkeys and apes) of the early Miocene (ca. 23-16 Ma) are thought to have been taxonomically diverse, the range of locomotor diversity in this group has been more difficult to characterise due to a relative lack of fossil evidence. In accord with early and influential narratives positing primate locomotor evolution to have occurred in a series of distinct stages^1,2^, most early Miocene catarrhines are thought to have practiced a similar and somewhat limited set of positional behaviours^3^. This inference, derived largely from functional analysis of the extensive postcranial hypodigm of *Ekembo heseloni*^4–7^, is consistent with above-branch palmigrade quadrupedalism with occasional bouts of slow climbing or careful clambering^8–17^, and perhaps some leaping^18,19^. This behavioural repertoire has essentially become the null hypothesis in evaluating early Miocene catarrhine positional behaviour, with most postcrania thought to be insufficiently distinct from *E*. *heseloni* to indicate discernable behavioural divergence^3,20–22^. Derived positional behaviours (i.e., orthogrady rather than pronogrady, suspension rather than above-branch positional behaviours, versatile and/or acrobatic climbing involving highly abducted limbs rather than largely parasagittal limb movements, and substantial terrestriality) are not generally considered to have begun emerging until the middle Miocene (ca. 16-12 Ma), as represented by the identification of incipient terrestriality in *Equatorius africanus*^23–25^ and enhanced climbing ability in *Nacholapithecus kerioi*^26,27^, with more substantial locomotor adaptation and diversification occurring only later during the late-middle Miocene of Eurasia^28,29^.

Efforts to evaluate this narrative are hindered by a lack of fossil evidence, as early Miocene catarrhine postcrania tend to be rare, fragmentary, and unassociated^30–33^. Despite these limitations, several studies have argued for the presence of derived positional behaviours in some early Miocene taxa. For example, the stem catarrhines *Dendropithecus macinnesi* from Rusinga and *Simiolus enjiessi* from Kalodirr are thought to have some adaptations for climbing^20,34^ or suspension^4,10,11,35–37^. Among putative apes, *Morotopithecus bishopi* demonstrates adaptation for orthograde climbing^38–41^, and *Turkanapithecus kalakolensis* may also have had enhanced climbing abilities^11^. At Songhor, a mid-sized proximal femur has also been interpreted to evince adaptation for climbing and suspension^21,30^, two medial cuneiforms seem to resemble extant apes more than *E*. *heseloni*^42^, and a sample of hallucal metatarsals displays morphological diversity potentially related to function^32^. Nevertheless, evidence of derived behaviours is still often deemphasised in reviews of early Miocene catarrhine evolution^21,22^, and there has been no attempt to quantify locomotor diversity among early Miocene catarrhines.

**Figure 1:**
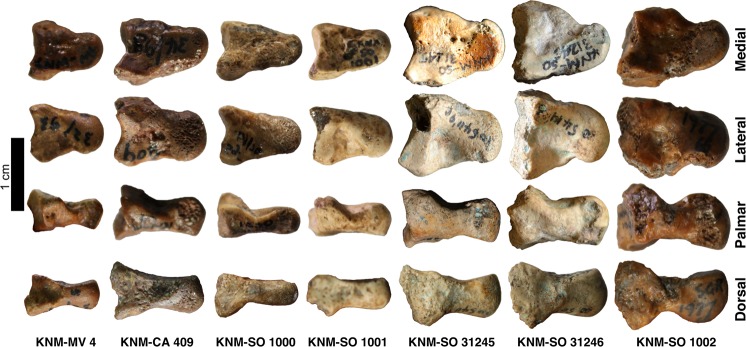
Tinderet fossil capitates, roughly to scale. KNM-SO 1000 was mirrored for ease of comparison. See Fig. S2 for additional views. Photos by C.W. and I.O.N.

Seven catarrhine capitates (Fig. 1) were recovered between 1931 and 1996 from Songhor, Chamtwara, and Mteitei Valley, penecontemporaneous sites within the Tinderet Miocene sequence of Western Kenya (Fig. S1, Table S1b) dated to around 20 Ma^43,44^. This sample provides a rare opportunity to evaluate locomotor diversity among the catarrhines of a geographically and temporally constrained period predating the Eurasian hominoid radiation^45^. While several of these specimens are discussed in the work of previous researchers^28,30^, none had been formally described. We compare the Tinderet capitates to a broad sample of extant anthropoids as well as *Ekembo heseloni* (Table S1) using morphometrics derived from 3D models (Table 1, Fig. S4; see also SI and ref. ^46^). Functional morphology is assessed with positional classifiers built using linear discriminant function analysis (DFA) and *glmnet*, an elastic net-regularised multinomial logistic regression machine learning algorithm^47^, and by quantitative estimation of locomotor proportions as well as qualitative comparisons. We attempt to constrain the species identity of each fossil specimen by considering these results in concert with body mass estimates, taxonomic classifiers and hierarchical clustering. Finally, we use an approach derived from two-block partial least squares analysis (PLS)^48^ to create, for the first time, a quantitative estimate of locomotor diversity in a fossil catarrhine sample relative to that of extant catarrhine groups, and discuss implications for our understanding of catarrhine evolution.

**Table 1:** Capitate shape metrics.

Variable name	Definition	Normalization
CpPx^a^	Proximoradial facet surface area	Capitate surface area
CpSc	Scaphoid/centrale facet surface area	Capitate surface area
CpLu	Lunate facet surface area	Capitate surface area
CpDn	Dorsal nonarticular surface area	Capitate surface area
Cp3	Mc3 facet surface area	Capitate surface area
CpHm	Hamate facet(s) surface area	Capitate surface area
Cp2	Mc2 facet(s) surface area	Capitate surface area
Cp4	Mc4 facet(s) surface area	Capitate surface area
Cp23A	Mc2-Mc3 facet angle	na
Cp3HmA	Mc3-hamate facet angle	na
CpPxA	Proximal angle^b^	na
CpScA	Scaphoid/centrale-dorsal nonarticular angle	na
Cp3SD	Mc3 facet complexity^c^	Cube root of capitate volume
CpHmC	Hamate surface concavity^d^	Proximodistal length of hamate facet
CpHP	Dorsopalmar head position^e^	Square root of Mc3 facet surface area

^a^Sum of CpSc and CpLu. Not included with its constituent metrics in multivariate analyses.

^b^Calculated between proximoradial (scaphoid/centrale + lunate) and hamate facets.

^c^Standard deviation from fitted plane, weighted to account for triangle size.

^d^Difference between maximum and minimum deviance from fitted plane.

^e^Linear distance from most proximal point to plane of dorsal nonarticular surface.

## Results and Discussion

Results of this study sort the seven Tinderet capitates into four groups: small- and medium-sized arboreal quadrupeds, a small suspensory form, and a derived, medium-sized morph with great ape affinities. When combined with the large quadruped *Proconsul major*, also present in this setting but not represented in the sample, at least five niches are currently distinguishable from postcrania, close in number to the 6 genera (8 species) recognised dentally from these sites (Table 2).

**Table 2:** Fossil catarrhines recognised at Songhor, Chamtwara, and Mteitei Valley.

Taxon	Localities	Body mass^a^	Refs
*Proconsul africanus*	SO, CA, MV	8–19 kg^b^	^21^
*Proconsul major*	SO, CA, MV	63–87 kg^c^	^100^
*Rangwapithecus gordoni*	SO	8–19 kg^d^	^33, 101^
*Dendropithecus macinnesi*	SO, CA	6–8 kg	^102, 103^
*Kalepithecus songhorensis*	SO, CA, MV	5–6 kg	^102^
*Limnopithecus evansi*	SO, MV	5 kg	^21^
*Limnopithecus legetet*	CA	5 kg	^21^
*Micropithecus clarki*	CA	3–4.5 kg	^102, 104^

^a^Published estimates; those for species other than *P*. *major* are based on qualitative comparisons.

^b^Estimate of *E*. *heseloni*, to which *P*. *africanus* is thought to be similar in size, albeit with perhaps slightly smaller teeth^21^. *E*. *heseloni* estimate based on regression of postcranial articular sizes or shaft dimensions and extrapolation from extant ontogenetic data in refs^100,105^.

^c^Based on linear regression of tibial and humeral shaft dimensions and talar and tibial articular size.

^d^Based on dentognathic size similarity with *E*. *heseloni*, although many postcrania attributed to *Rangwapithecus* exceed this estimate’s upper bound^32,52^.

### Group 1: Small arboreal quadrupeds

**Figure 2:**
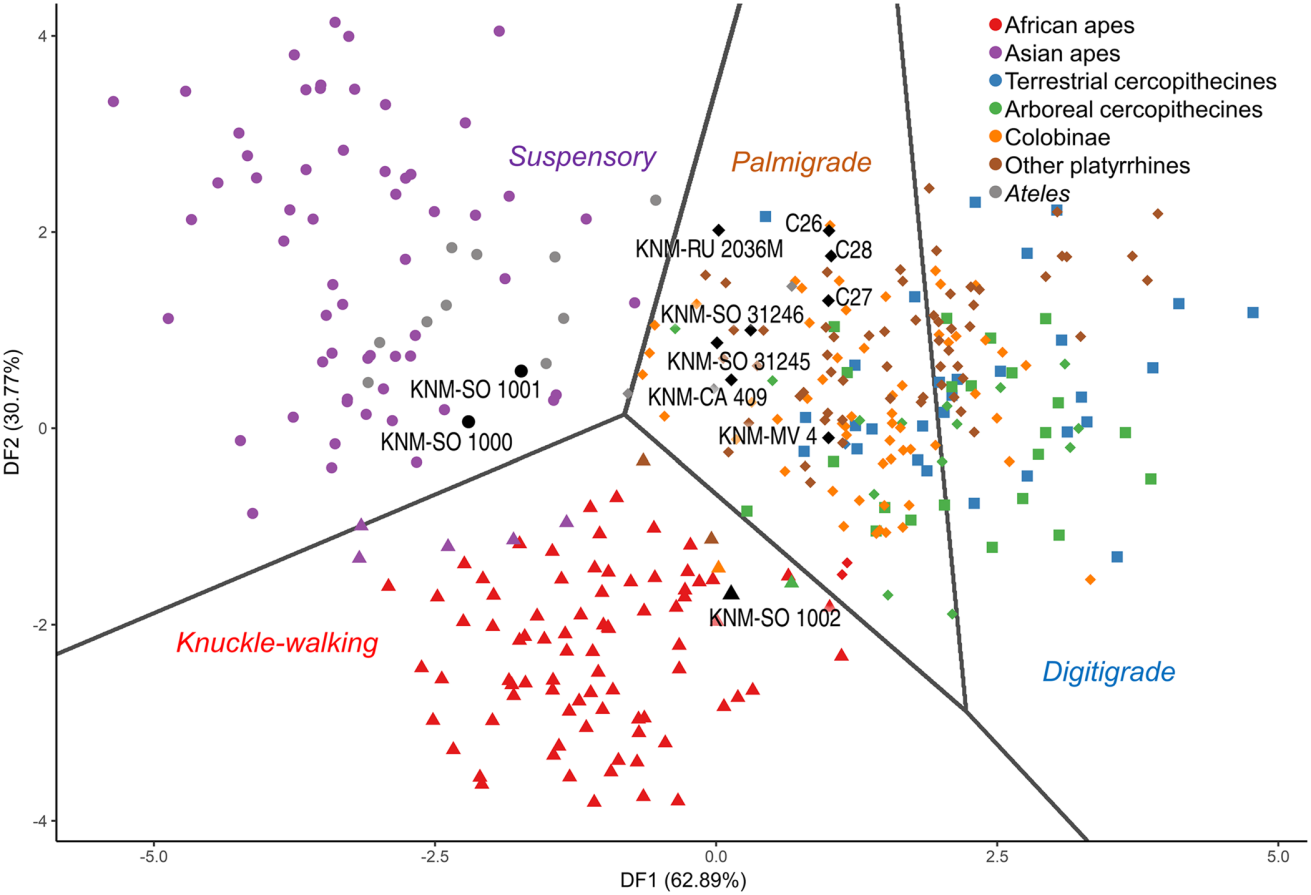
Discriminant scores based on nine shape variables best distinguishing extant positional classes. Points are coloured according to *a priori* class and shaped according to predicted class: circles, suspensory; triangles, knuckle-walking; squares, digitigrade; diamonds, palmigrade. Grey lines represent decision boundaries. See Tables S7 and S8 for model details and accuracy metrics.

KNM-MV 4 is the smallest, and in some ways most primitive, specimen of the sample. It has qualitative resemblance to some platyrrhine specimens, including a wide and medially-tilted palmar portion of the Mc4 facet and a mediolaterally expanded and relatively planar Mc3 facet (see SI for detailed morphological descriptions), and is grouped among them by the taxonomic DFA (Fig. S8). The positional classifiers identify it as a palmigrade quadruped (Fig. 2, Tables 3 and S7), a finding corroborated by its estimated locomotor proportions (Tables 3 and S12). Among species recognised dentally at Mteitei Valley, its size comports with *L*. *evansi* and *K*. *songhorensis*. As these species are unknown postcranially, a more definitive attribution is not currently possible. Of species known from other Tinderet sites, this specimen is also compatible in size with *L*. *legetet* and, to a lesser extent, *M*. *clarki* and *D*. *macinnesi*, although it lacks derived morphology linking it with the latter species.

**Table 3:** Summary of fossil specimen affinities.

Specimen	BM (kg)	Positional classification				Locomotor proportions				Plausible identity^a^
		DFA	prob	*glmnet*	prob	*QuadA*	*Quad*	*SuspA*	*Susp*	
KNM-MV 4	5.3	*PG*	0.94	*PG*	1.00	0.35	0.57	0.07	0.03	*L*. *evansi*, *K*. *songhorensis*
KNM-CA 409	9.2	*PG*	0.80	*PG*	0.99	0.61	0.54	0.06	0.08	***cf***. ***P***. ***africanus***
KNM-SO 1000	6.0	*S*	0.90	*S*	1.00	0.37	0.24	0.19	0.19	*cf*. *D*. *macinessi*, *L*. *evansi*, *K*. *songhorensis*
KNM-SO 1001	5.9	*S*	0.95	*S*	1.00	0.16	0.50	0.32	0.10	*cf*. *D*. *macinessi*, *L*. *evansi*, *K*. *songhorensis*
KNM-SO 31245	12.4	*PG*	0.95	*PG*	0.90	0.40	0.33	0.11	0.06	***cf***. ***P***. ***africanus***, *R*. *gordoni*
KNM-SO 31246	15.4	*PG*	0.96	*PG*	0.96	0.41	0.32	0.09	0.06	***cf***. ***P***. ***africanus***, *R*. *gordoni*
KNM-SO 1002	15.9	*KW*	0.89	*KW*	0.83	0.42	0.76	0.04	0.01	***R***. ***gordoni***, *P*. *africanus*
*E*. *heseloni* (means)	11.5	*PG*	0.75	*PG*	0.95	0.49	0.45	0.09	0.08	

^a^Species in bold are provisionally preferred.

### Group 2: Small suspensors

KNM-SO 1000 and KNM-SO 1001 are estimated to have been substantially reliant on below-branch behaviours, with both grouped among extant suspensors by the positional classifiers (Fig. 2, Table S7). Suspensory estimates for KNM-SO 1000 approach those observed in *Ateles* (Tables 3 and S11). Estimates of arboreal proportions (*QuadA* and *SuspA*) align KNM-SO 1001 with *Pongo*, but total proportions (*Quad* and *Susp*) differ only slightly from those of *E*. *heseloni* (Table S12). Both specimens are relatively narrow, with low hamate facet concavity and small, radially-oriented centrale facets, features associated with suspension^49^. KNM-SO 1000 has additional, qualitative features linking it with suspensory taxa. Its relatively small and discontinuous Mc2 articulation may indicate hypertrophy of the lateral carpometacarpal ligament, hypothesised to aid suspension^50^, although in the current sample this trait was variably present in each of the anthropoid subfamilies, with no apparent functional correspondence (Table S6a). Qualitative observations have been used to argue that the size of the canal transmitting this ligament is the more diagnostic feature^51^, a conclusion not examined here. KNM-SO 1000’s hamate facet is also discontinuous (Fig. S6), a trait typical only in brachiators of the extant sample (*Ateles* and the hylobatids) and otherwise found only in a minority of *Pongo* and *Nasalis* (Table S6b). This feature may relate to hypertrophy of the capitohamate interosseous ligament, potentially stabilizing this joint against sudden load transmission gradients experienced during brachiation or other acrobatic arborealism.

**Figure 3:**
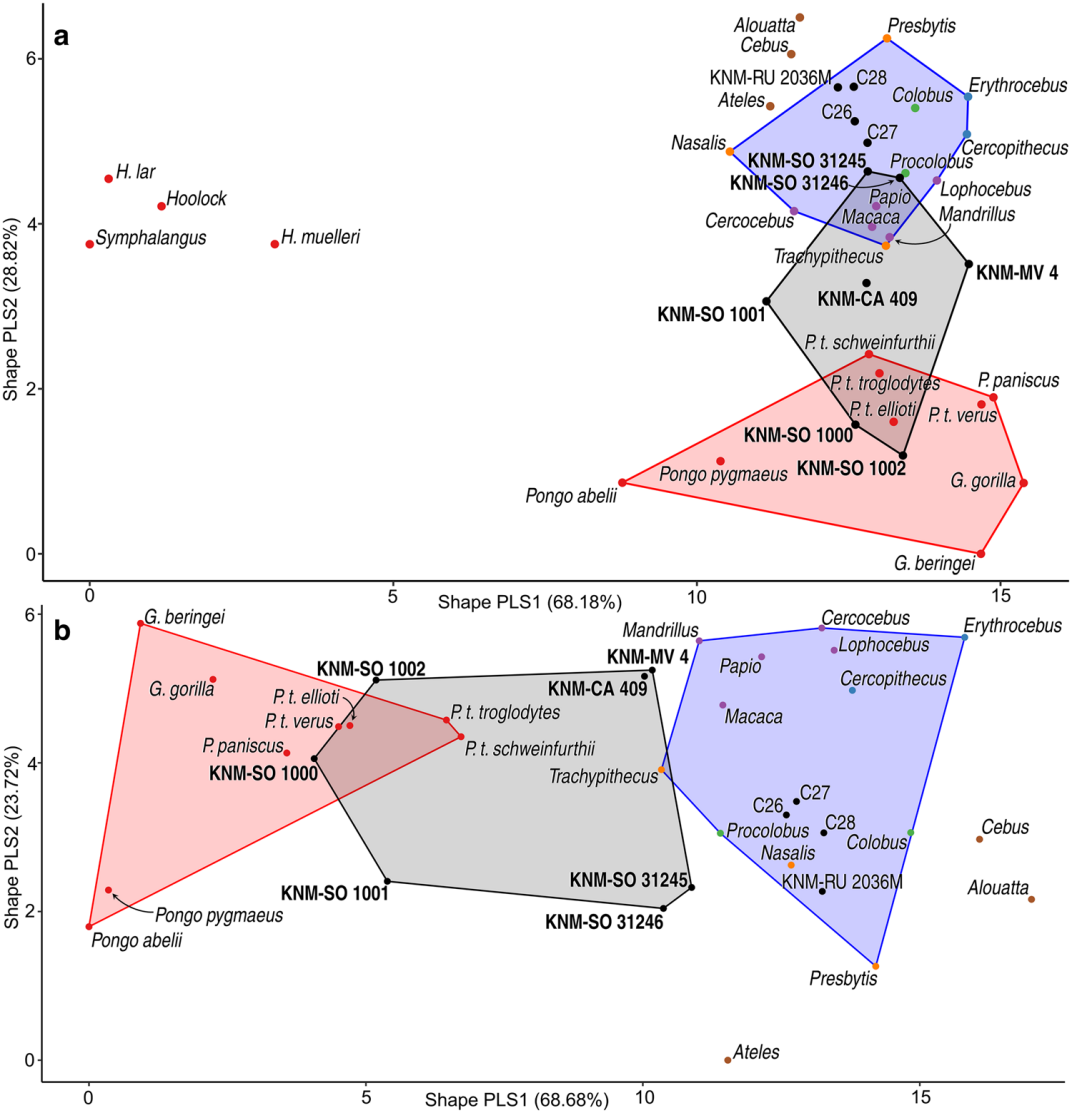
PLS shape-space with convex hulls characterizing the functional diversity of the Tinderet sample (shaded grey) relative to extant great apes (shaded red) and Old World monkeys (shaded blue), in the full sample (**a**) and with hylobatids excluded (**b**).

KNM-SO 1000 and KNM-SO 1001 are only slightly larger than KNM-MV 4, and therefore potentially compatible with the same set of taxa, although they further exceed the estimated size range of *M*. *clarki*. The suspensory features of these specimens are consistent with interpretations of *D*. *macinnesi*^4,10,11,35–37^, but confident attribution to this taxon is precluded by the potential for similar adaptations in the yet-unknown postcrania of the other small-bodied catarrhines at Songhor. Furthermore, although a strong suspensory signal is identified in both KNM-SO 1000 and KNM-SO 1001, the discussed dissimilarities (see also Figs 3 and S7) raise the possibility that these specimens are not conspecific, which would suggest derived arborealism in one of the other small-bodied Songhor taxa. These specimens are therefore only tentatively referred to *cf*. *D*. *macinnesi*.

### Group 3: Medium arboreal quadrupeds

KNM-CA 409, KNM-SO 31245, and KNM-SO 31246 are all reconstructed as mid-sized palmigrade quadrupeds resembling *E*. *heseloni*. The latter two specimens show particular affinity with *E*. *heseloni* in functional models (Figs 2 and 3, Table 3). KNM-CA 409 demonstrates less quantitative similarity with the Rusinga specimens (Figs 3b and S7) and its estimated locomotor proportions differ somewhat from those predicted for other medium-bodied quadrupeds, while its body mass estimate is lower than other group 3 specimens. However, it has the greatest qualitative resemblance to *E*. *heseloni* of the fossil sample, including the presence of the dorsodistal lip formerly exclusive to this taxon (Fig. S5), and it falls within the lower limit of the size range estimated for the medium-bodied taxa (Table 2).

The prevalence of *R*. *gordoni* relative to *P*. *africanus* at Songhor has been used to justify assigning mid-sized postcrania from this site to the former taxon^30,32,42,52^, but *R*. *gordoni* dental remains are only about twice as prevalent^53^, rendering this criterion uncompelling in the allocation of individual specimens. Given the discussed affinities of these specimens with *E*. *heseloni* and the close phylogenetic relationship of this taxon with *P*. *africanus*^21^, with which it was until recently thought to be congeneric^54^, we provisionally attribute the group 3 specimens to *P*. *africanus*.

### Group 4: Medium derived morph

KNM-SO 1002 is only slightly larger than the group 3 specimens, but its morphology is distinct. While in some ways, particularly its articulations with the Mc2 and Mc3, it remains similar to those of most other early Miocene specimens including *E*. *heseloni* (see SI), it pairs these ancestral traits with derived ones, reinforcing the piecemeal nature of catarrhine functional evolution^29,55,56^. Its large, globular, and laterally-expanded head, highly-waisted neck, medially and laterally expanded body, moderately-expressed dorsal ridge, and concave hamate facet contribute to a profile in dorsal or palmar view that is uniquely great ape-like among early or middle Miocene capitates (Figs 1 and S6), an especially surprising finding given the early date. These observations are born out in classification and hierarchical models, which group this specimen with African apes (Figs 2, 3, S7 and S8; Table S7). It is estimated to have the highest overall reliance on quadrupedalism of the fossil sample, matching the estimated (but not observed) African ape values (Tables S11 and S12). Its suspensory estimates are lower than the other fossils, with proportions corresponding to the baseline values assigned to extant non-suspensors.

While the locomotor behaviour of this specimen was therefore likely distinct from the others of the fossil sample, characterization of these differences is difficult. A waisted neck and dorsal ridge have been interpreted to contribute to a functional midcarpal complex reflecting limited extension and enhanced transmission of loads generated during knuckle-walking^57–59^, but this interpretation has been challenged on the basis of the relative rarity of these and other reputed knuckle-walking traits in *Gorilla*, their inconsistent presence in *Pan*, and their variable presence in non-knuckle-walking taxa^60–64^. The condition of KNM-SO 1002, also present to a lesser extent in KNM-SO 31246, in which the distal extent of the centrale facet is positioned palmarly to a laterally-projecting portion of the body’s dorsal margin (Fig. S7), would intuitively reflect enhanced midcarpal stability at full extension during knuckle-walking. However, this condition appears in sampled *Pongo* specimens roughly as often as in *Gorilla*. In most *Pan* specimens, but only occasionally in *Gorilla*, an elaborated condition is found in which the entire dorsum of the body projects laterally, reorienting a sizable portion of the scaphoid facet palmarly and creating a sharply-angled margin that is often confluent with a raised dorsal ridge separating the head and body (Fig. S7, inset). The greater prevalence of this morphology in *Pan* may be explained, as other reputed knuckle-walking traits have been, as reflecting a greater degree of wrist extension during stance phase relative to *Gorilla*^63,65–67^. However, sampled hylobatids frequently possess morphology matching the most pronounced African ape examples of this trait (Fig. S7h). Therefore, in addition to its plausible association with enhanced stability at limited midcarpal extension during knuckle-walking, this morphology likely has additional utility, perhaps reflecting a need to prevent over-pronation of the midcarpal joint (or over-supination of the forearm below a fixed grasp) during vertical climbing or suspension. While the lower incidence of this morphology in *Gorilla* could reflect their lesser reliance on these behaviours in adulthood, it would not account for similar reliance on these behaviours in early subadults of the two genera^68,69^, nor the presumably increasing utility of this morphology with body size^63^.

Whether this morphology is related to multiple positional behaviours, facilitates some unrecognised kinematic affinity among hominoids, or arose from nonadaptive factors, these findings support the view that classical knuckle-walking traits are not exclusively associated with knuckle-walking, and highlight the continuing difficulty of inferring this behaviour from wrist morphology^60–64^. Deciphering the positional repertoire of KNM-SO 1002 will therefore require a detailed understanding of differential carpal biomechanics among extant apes. While the presence of a knuckle-walking ape in this early setting would evince the homoplastic evolvability of the behaviour^70^, a hypothesis that KNM-SO 1002 practiced knuckle-walking is not favoured here. This specimen nevertheless adds to a growing body of evidence supporting the presence of a derived ape at Songhor. Although it falls within the estimated size range of *P*. *africanus*, its derived morphology strongly suggests it is taxonomically distinct from the group 3 specimens. It is provisionally attributed to *R*. *gordoni*.

### Early Miocene locomotor diversity and implications for hominoid evolution

The Tinderet sample occupies a greater proportion of the full-sample PLS shape-space (Fig. 3a) than do the cercopithecoids (6.41 vs. 5.58%; Table S13c), with the great ape value being even higher (8.93%). Because the shape-space structure (discussed in SI) is dominated by the extreme values of the hylobatids, the analysis was repeated with them excluded. In the resulting shape-space (Fig. 3b), the locomotor diversity estimate of the Tinderet capitates (17.18%) exceeds that of the great apes (12.96%) and cercopithecoids (15.43%), despite the extant groups being represented by a larger number of data points. While interpretation is complicated by the likely inclusion of both hominoids and stem catarrhines in the Tinderet sample, as well as the comparison of fossil individuals to extant taxon centroids, it suggests that the catarrhine clade had already undergone substantial functional diversification by 20 million years ago, resulting in the use of a broader range of positional repertoires than previously thought to characterise the clade’s early Miocene representatives.

Particularly intriguing among the range of forms documented in this study is that represented by KNM-SO 1002. Its morphology lends further support to the presence of a mid-sized catarrhine at Songhor with a behavioural repertoire more similar in some ways to that of extant great apes than to *E*. *heseloni*. The identity of this taxon cannot be determined with current evidence, but *R*. *gordoni* is currently the best candidate. Derived features have previously been identified among the nyanzapithecines^11,71^, of which *R*. *gordoni* is perhaps a basal member^72–74^, and this clade may be more closely related to crown hominoids than to *Proconsul* or *Ekembo*^74–76^, although there is not consensus on this point^22^. Implicit in the provisional allocation of KNM-SO 1002 to *R*. *gordoni* is the suggestion that the presence of derived, great ape-like features may be a useful criterion by which to distinguish the postcrania of *R*. *gordoni* and *P*. *africanus*. This hypothesis predicts that medium-sized postcranial specimens with similarly derived morphology should occur at Songhor and Kapurtay, but not at Koru, Chamtwara or Legetet, from which *Rangwapithecus* dental specimens have not been recovered.

Whether ape-like traits preserved at Songhor (and Moroto^39^) offer a glimpse of the ancestral crown hominoid morphotype or only of early examples of the evolutionary experimentation characterizing later hominoid evolution is difficult to address with current evidence. Homology cannot be ruled out, as although the mosaicism of later hominoid evolution has changed the calculus regarding the parsimony of extant ape homology^3,45,77–79^, the hierarchical nature of homoplasy supports the parsimony of some degree of homology in cases of derived morphology shared among extant ape lineages^80^. Furthermore, the time period represented at these Tinderet localities may coincide with the cladistic event separating hylobatids from great apes (mean: 20.19 Ma, median: 19.43 Ma^81^). The less derived anatomy of later African catarrhines decreases the likelihood of this explanation, however.

While the depositional and diagenetic environments of Miocene Tinderet sediments seem to have been unconducive to the preservation of associated crania and postcrania, continued work at these and other early Miocene sites leading to additional sets of overlapping postcranial elements like that presented here will be important in further characterizing functional diversity among early catarrhines. The morphological diversity of this sample offers a snapshot of differing lifestyles among a community of penecontemporaneous catarrhines and demonstrates a degree of functional diversity beyond what is generally accepted to have been present in early Miocene catarrhines. Although confident conclusions cannot be made based on a single skeletal element, the morphological diversity among these capitates raises the possibility of early hominoids and their contemporaries having diversified to fill multiple ecological niches early in their evolutionary career, well before the previously-inferred locomotor diversification of the later Miocene of Eurasia.

## Materials and Methods

### Shape data and body mass estimation

15 metrics characterizing articular surface area ratios, angles between regions of interest, and other aspects of shape (Table 1) were extracted from 3D models derived from μCT or laser scans of the Tinderet sample, four *E*. *heseloni* specimens, and a sample of 343 capitates from 28 extant taxa (detailed in SI; see ref.^46^ for further details and additional discussion. See refs^82–84^ for use of similar metrics). Body mass was estimated based on capitate volume, whose suitability for this purpose was evaluated via log-log ordinary least squares (OLS) regression against sex-specific body mass data gathered from museum records of sampled specimens when available, supplemented by published data^85,86^. Homogeneity of regression slopes between sexes and superfamilies was confirmed via analysis of covariance. Because sex-specific means were used to predict individual body masses, confidence intervals are not meaningful^87^. Prediction error was therefore characterised with residual standard error (0.147), percent mean prediction error (12.36%), and percent standard error of the estimate (%SEE; 16.15%) after 100 repetitions of 10-fold cross validation (CV).

### Functional and systematic affinities

Extant taxa were assigned to one of four broad classes characterizing the dominant positional behaviour in each taxon’s repertoire: *KW* = knuckle-walking; *S* = orthograde climbing, clambering, suspension and/or brachiation; *PG* = arboreal palmigrade quadrupedalism; *DG* = terrestrial digitigrade quadrupedalism (Table S1; see also SI text). Two positional classifiers were built from the shape variables found to covary with extant positional classes using phylogenetic generalised linear mixed modelling^88–91^ (detailed in SI). Linear DFA was chosen for its interpretability and ease of visualization, while *glmnet* was chosen for being less prone to overfitting and bias due to collinearity, less stringent in its assumptions regarding heteroscedasticity, and its ability to detect non-linear relationships^92,93^. The accuracy of each classifier was calculated after 100 repetitions of 10-fold CV with random, non-stratified sampling, chosen for its favorable combination of variance reduction and low bias relative to other CV techniques^94^. Taxonomic classifiers were also built following the same methods (Fig. S8). Fossil systematic affinities were further explored via BIONJ^95^, a neighbour-joining hierarchical clustering algorithm (Fig. S9).

Locomotor behaviour in the extant sample was also characterised by continuous variables based on published observations available for 22 of 28 sampled taxa (Table S1a). These metrics represent the proportion of locomotor time spent in four different modes: *Quad* = quadrupedalism/tripedalism; *Susp* = orthograde suspension, including brachiation, forelimb swinging, orthograde clambering/transferring, and inverted walking/running (after ref.^96^); *Climb* = vertical climbing, quadrupedal climbing and scrambling, vertical descent, bridging, sliding, and swaying (after ref.^97^); *Leap* = leaping and dropping (see ref.^98^ for definitions and discussion of behavioural terms). Each mode is represented by an additional variable (e.g., *SuspA*) representing its use as a proportion of the taxon’s arboreal locomotion. A final locomotor variable, *Arb*, represents the proportion of locomotion occurring on arboreal rather than terrestrial substrates. Bipedal proportions were also compiled but are not included here due to an expected lack of morphological association. The total and arboreal proportions of some taxa therefore do not sum to 1.

Models to estimate locomotor proportions were built in a multi-step process. After eliminating shape variables found not to covary with the locomotor variable under consideration using PGLS with size as a covariate, subsets of the remaining shape variables were ranked by ascending second-order Akaike Information Criterion (AICc), a metric useful in balancing the opposing concerns of model accuracy and reduced generalisability due to overfitting^99^. The best three or four variable subsets, depending on similarity of delta-AICc values, were used to predict that locomotor proportion in the extant sample using quasibinomial logistic regression. The most accurate of these models, as judged by %SEE after 100 repetitions of 10-fold CV, was chosen for use in estimating that locomotor proportion in the fossil specimens. Climbing, leaping, and arboreality were found to have insufficient covariance with capitate morphology to produce accurate predictive models, so only those for the quadrupedal and suspensory proportions are reported.

### Assessment of locomotor diversity

Two-block PLS analysis was used to create a two-dimensional shape space in which covariance with locomotor behaviour is maximised along each axis. The locomotor block consists of the four arboreal proportions along with the proportion of arboreality, a combination selected as having the strongest relationship with carpal morphology (detailed in ref.^46^). The fossil specimens and centroids of extant taxa for which locomotor proportions are unavailable were projected into PLS shape-space by multiplying their scaled shape variable matrix by the singular vectors of the PLS shape block. The functional diversity of the Tinderet sample was then estimated relative to that of extant groups by calculating the Euclidean area of the convex hull enveloping the constituent data points of each group as a proportion of PLS shape-space. See SI for additional analysis, results, and discussion.

## Supplementary information

Supplementary info
