# Supplementary material for: Wrist morphology reveals substantial locomotor diversity among early catarrhines: an analysis of capitates from the early Miocene of Tinderet (Kenya)
Source: Sci Rep. 2019 Mar 6;9:3728. doi: 10.1038/s41598-019-39800-3 (PMC6403298; doi:10.1038/s41598-019-39800-3)
Supplement: Supplementary file 1 — Supplementary info [file 41598_2019_39800_MOESM1_ESM.pdf]

Supplementary information – Wrist morphology reveals substantial locomotor diversity among early catarrhines: an analysis of capitates from the early Miocene of Tinderet (Kenya)

Craig Wuthrich[1,2*], Laura M. MacLatchy[1], and Isaiah O. Nengo[3,4]

[1] Department of Anthropology, University of Michigan, Ann Arbor, MI 48109
[2] Department of Evolutionary Anthropology, Duke University, Durham, NC 27708
[3] Turkana Basin Institute, Stony Brook University, Stony Brook, NY 11794
[4] Turkana University College, P.O. Box 69-30500, Lodwar, Kenya
[*] Corresponding author: craig.wuthrich@duke.edu

Discovery and context

Five of the Tinderet capitates were recovered at Songhor (35° 12.6' E, 00° 02' S), a site discovered in 1932 by L.S.B Leakey and Donald MacInnes[1]. This locality's fossiliferous sediments, accumulated by sub-aerial deposition, consist of a sequence of tuffs divided into four main units, from oldest to youngest – the Calcified Tuff Member, the Red Bed Member, the Grey Tuff Member, and the Tuff and Agglomerate Member[2]. A sample from a biotite collected at the base of the calcified Tuff Member, the oldest of the four, yielded a K/Ar age estimate of between 19.2 and 20.5 Ma[3]; adjustment for new radiometric constants produce a marginally earlier range of 19.7-21 Ma[4]. Efforts to re-date this and many other East African Miocene sites are currently underway[5]. KNM-SO 1000, 1001, and 1002 were recovered during the Leakey and MacInnes excavation of 1966. KNM-SO 31245 and 31246 were recovered in 1996 after renewed excavation and sieving of Red Bed Member sediments by Nengo and colleagues[6] in collecting area 5 of Pickford and Andrews[2].

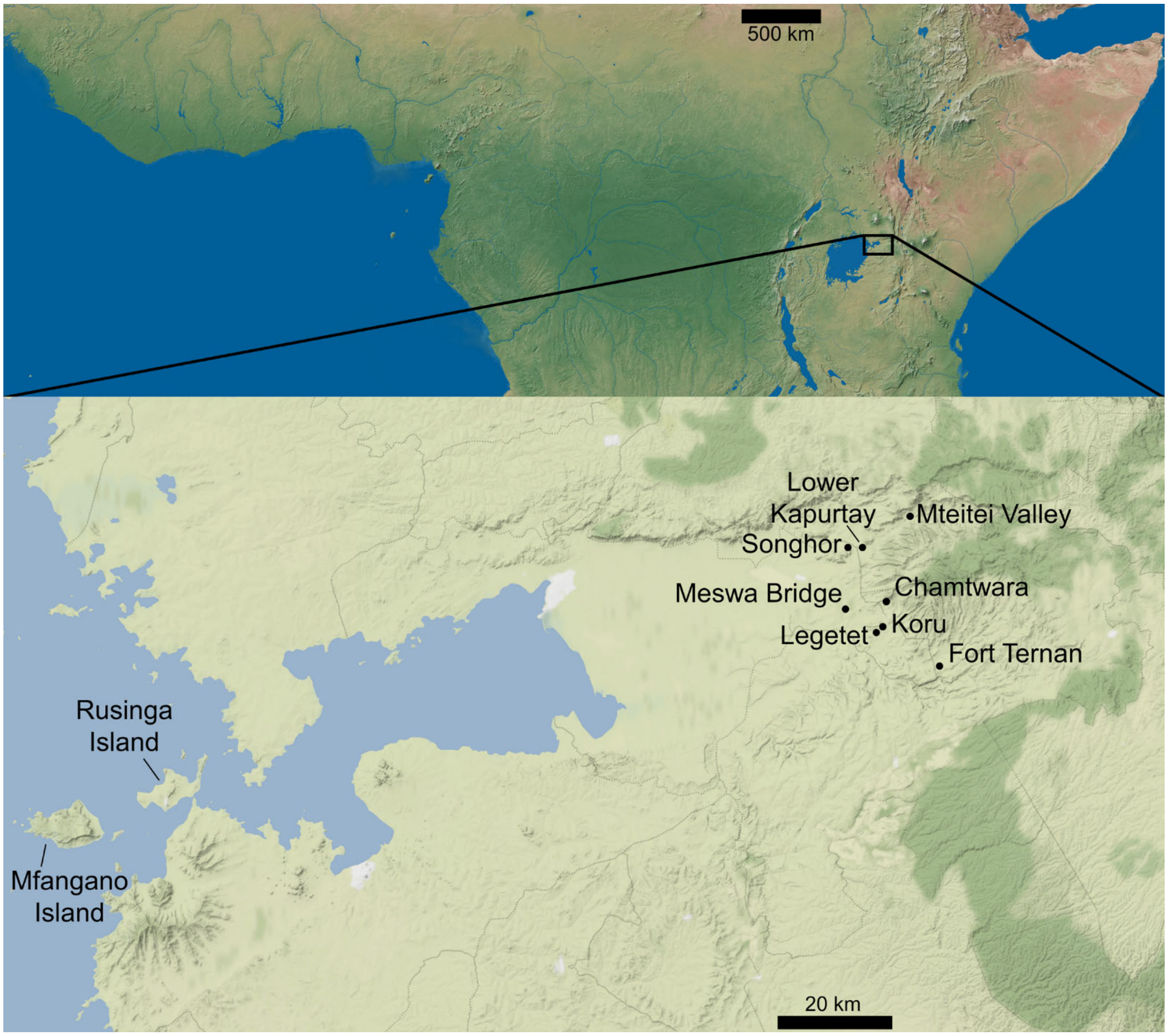

Fig. S1. Sites preserving Miocene catarrhine specimens, including those analysed here. Maps courtesy *Natural Earth III* (shadedrelief.com), Stamen Design under CC BY 3.0, © openstreetmap.org contributors under ODbL, cartography licensed as CC BY-SA. GPS coordinates from refs [7-11] and K. McNulty (pers. comm.).

The two remaining specimens were discovered at nearby Chamtwara (35° 15' 57.6" E, 0° 07' 32.6" S) and Mteitei Valley (35° 18' 30.75" E, 0° 00' 40.6" N), apparently by Leakey and MacInnes' 1930s expeditions (based on field numbers). Despite their relative temporal and spatial proximity (Fig. S1), these three Tinderet sites have distinct fossil assemblages (see Table 2): *P. africanus*, *P. major*, and *K. songhorensis* are common to all, but *D. macinnesi* has been identified only at Songhor and Chamtwara of the Tinderet sites[12]. *L. legetet* and *M. clarki* are common at Chamtwara but not known at Songhor or Mteitei Valley, which instead yield *L. evansi*[13,14]. *R. gordoni* may have been a temporally and geographically restricted taxon[7], perhaps associated with a specialization for folivory[15, but see 16]). It is known almost exclusively from Songhor, save for a mandible[7] and partial cranium[17] from Lower Kapurtay (35° 13.4' E, 00° 02' S), an adjacent site discovered in 1996 by Nengo and Malit[8], and a lower third molar from Moroto II, Uganda provisionally attributed to this genus[18]. A ninth taxon, *Xenopithecus koruensis,* is known only from a fragmentary maxilla from the Tinderet site of Koru, and is not known from Songhor, Chamtwara or Mteitei Valley[12]. For this reason, it is not viewed as a candidate taxon for allocation purposes, although that could change with future discoveries.

SI Methods

Sample and data collection

The extant sample comprises 343 specimens from 28 extant taxa (Table S1a and Table S15), curated by the American Museum of Natural History (AMNH), the Smithsonian Institution's National Museum of Natural History (NMNH), Harvard University's Museum of Comparative Zoology (MCZ), the Cleveland Museum of Natural History (CMNH), and the University of Michigan Museum of Zoology (UMMZ). Extant taxa were assigned to positional classes (*KW*, *S*, *PG*, or *DG*) using published observations (Table S1). While categorical schemes are frequently used in analyses of primate behavioural variation[19-28], it is recognized that these are crude characterizations of primates' diverse behavioural repertoires[29,30]. Knuckle-walkers, for example, are also capable suspensors, but employ this behaviour infrequently in adulthood relative to those assigned to the *S* class. Following observations that digitigrade cercopithecines utilize more palmigrade-like hand postures at higher speeds[31,32], some researchers have taken to considering the two postures to be functionally indistinct. On the contrary, Patel and colleagues conclude that digitigrady is an adaptation to efficient long-distance walking[33]. It also remains plausible that this compliance at the wrist between touchdown and midstance, not available in palmigrade taxa, has an important functional role. A distinction between cercopithecine hand postures is therefore retained in this study to ensure that morphology associated with digitigrady[34,35] does not contribute to training the positional classifiers to recognize palmigrady.

The quantitative locomotor proportions used in this study attempt to more fully characterize the locomotor behaviour of the sampled taxa, but these data also have important limitations. Published observations are based on widely varying sample sizes, behavioural definitions and methods of quantification differ between researchers, and the proportional metrics attempt to characterize only adult locomotion, whereas non-locomotor postures such as arm-hanging while feeding have been proposed to play

important adaptive roles[36], and behaviours disproportionately preferred by sub-adults may strongly influence adult morphology[37]. Various averages and estimates were necessary to produce a useful data set (see Table S1 footnotes). For example, lowland gorillas are thought to be more arboreal than mountain gorillas[38-41], but this difference has never been quantified. It was therefore conservatively estimated as being nominal.

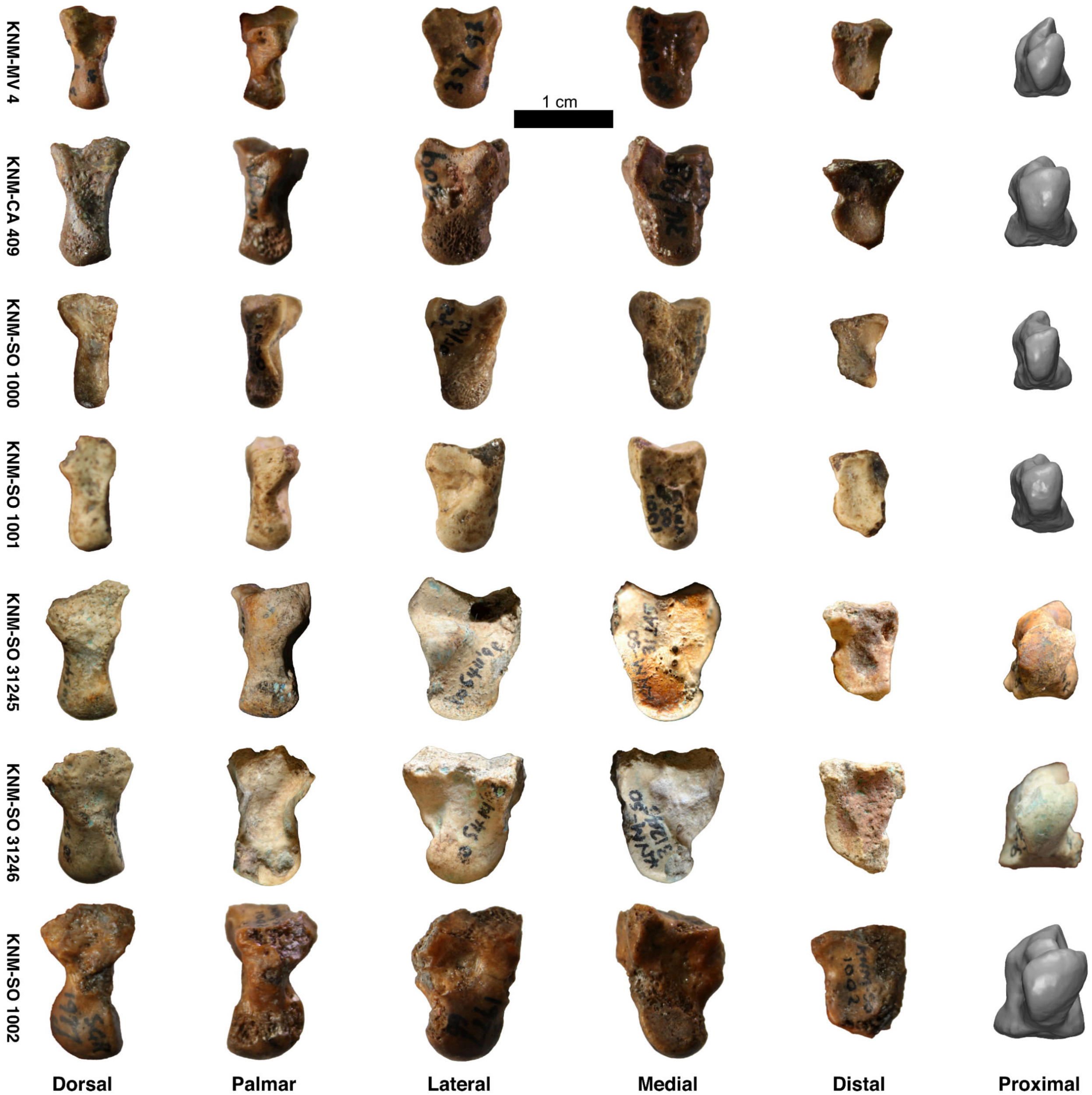

Fig. S2. Tinderet fossil capitates in standard anatomical views, roughly to scale. KNM-SO 1000 was mirrored for ease of comparison. Photos by C.W. and I.O.N. Views for which photographs were unavailable are visualized with 3D models.

Most of the extant sample (n = 291) were scanned with a GE Healthcare Pxs5-928EA μCT scanner. An isosurface was derived from each scan and exported as a triangular mesh. NMNH and CMNH specimens (n = 52) were scanned with a

NextEngine 3D Scanner HD, creating a triangular mesh comparable to those produced by the µCT workflow. The efficacy of pooling these two types of scan data has been demonstrated[42-44]. Qualitative observations were made on the original specimens at the National Museums of Kenya (NMK). Most fossils were sampled by µCT scanning casts ordered and shipped from NMK; KNM-SO 1002 was sampled via laser scanning. Measurements taken using digital calipers on the original specimens were compared to those taken from the Tinderet 3D models to assess their fidelity. Error in each case was less than 3%. The proximodistal length of KNM-SO 1002, for example, was measured at 13.2mm on the original specimen, 13.4mm on the model derived from laser scans (a difference of 1.5%), and 13.5mm on the model derived from µCT scans of a cast (differences of <1% and 2.2%, respectively). Some skepticism regarding the use of casts, even high-resolution, research-quality casts like those used here, is warranted. The use of 3D models derived from casts remains common, however (see refs [45-50] for recent examples).

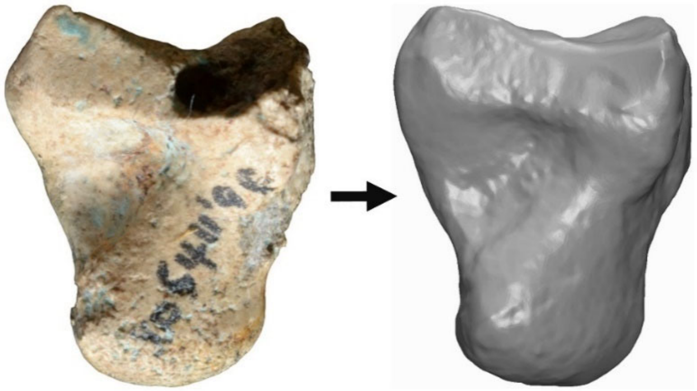

Fig. S3. Example of virtual reconstruction of missing morphology (KNM-SO 31245).

Extant models were processed and smoothed to correct imperfections stemming from the scanning process, osteochondral defects, or other issues of pathology or preservation to ensure comparability across the sample. These issues were minor; extant specimens with substantial defects were not sampled. Taphonomic damage to fossil specimens was virtually repaired, guided by the existing contour of the fossil and with reference to contemporaneous specimens (Fig. S3; see Figs. S2, S5, S6, and S7 for additional views of reconstructed 3D models). Morphometrics dependent on missing anatomy (7 values from 4 specimens) were imputed via a bootstrap-aggregated decision tree algorithm trained with the combined set of extant and fossil shape data, which fits a model for each variable as a function of all the others[51]. The 3D models were digitally segmented along articular margins and metrics characterizing articular size, angles, and other shape metrics were extracted (see Table 1). Dihedral angles were calculated between least-squares planes fitted to regions of interest. Metrics subject to isometry were normalized to render them scale-free[52] (see Table 1).

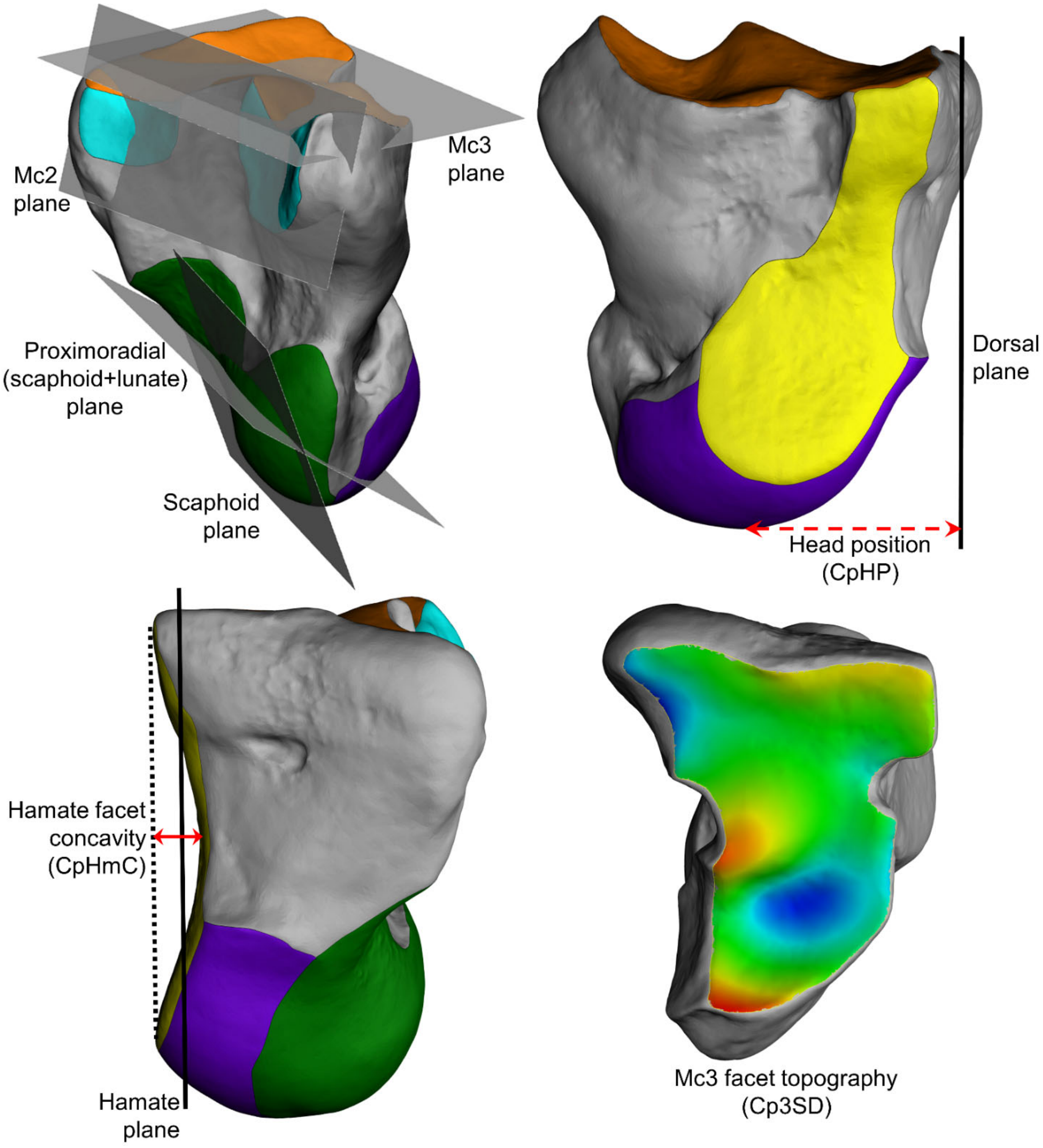

Fig. S4. Visualization of morphometrics and components thereof. Least-squares planes used for angular metrics are labelled. Articular facets are coloured as follows: hamate, yellow; lunate, purple; scaphoid/centrale, green; Mc3, orange; Mc2, light blue (Dorsal nonarticular surface visible in lower left; Mc4 not shown). See Table 1 for metric descriptions.

Table S1. Sampled extant (a) and fossil (b) capitates.

a

Taxon	n	♂	♀	Total proportions[a]					Arboreal proportions				Class	Refs
				Quad	*Susp*	*Climb*	*Leap*	*Arb*[b]	*QuadA*	*SuspA*	*ClimbA*	*LeapA*		
Pan troglodytes verus	13	7	6	0.86	0.01	0.11	0	0.16	0.21	0.06	0.68	0.01	*KW*	51,52
Pan t. ellioti	5	2	3										*KW*	
Pan t. schweinfurthii	10	7	3	0.93	0.01	0.06	0	0.10	0.31	0.08	0.59	0.02	*KW*	52,53
Pan t. troglodytes	12	6	6										*KW*	
Pan paniscus	4	2	2	0.87	0.01	0.09	0	0.17	0.35	0.09	0.51	0.04	*KW*	53c
Gorilla gorilla	26	15	11	0.92	0.01	0.06	0	0.10	0.19	0.13	0.62	0.02	*KW*	54d
Gorilla beringei	12	9	3	0.96	0.01	0.04	0	0.09	0.53	0.06	0.40	0.01	*KW*	55,56
Pongo pygmaeus	19	9	10	0.12	0.43	0.37	0.01	0.95	0.12	0.43	0.37	0.01	*S*	57e
Pongo abelii	15	5	10	0.18	0.38	0.35	0.01	0.95	0.18	0.38	0.35	0.01	*S*	58
Hoolock hoolock	7	3	4	0	0.55	0.20	0.22	0.99	0	0.55	0.20	0.22	*S*	59
Hylobates muelleri	4	2	2										*S*	
Hylobates lar	15	9	6	0	0.59	0.19	0.16	0.99	0	0.59	0.19	0.16	*S*	60
Symphal. syndactylus	3	1	2	0	0.59	0.32	0.02	0.99	0	0.59	0.32	0.02	*S*	61
Papio anubis	14	8	6	0.99	0	0.01	0.01	0.05	0.68	0	0.21	0.10	*DG*	36
Lophocebus albigena	6	5	1	0.42	0	0.36	0.21	0.95	0.42	0	0.36	0.21	*PG*	62,63f
Mandrillus sphinx	9	7	2										*DG*	32,64
Cercocebus agilis	2	2	0										*PG*	32,65
Macaca fascicularis	18	11	7	0.68	0	0.26	0.06	0.97	0.68	0	0.26	0.06	*PG*	66g
Erythrocebus patas	7	5	2	0.94	0	0.05	0.01	0.08	0.60	0	0.30	0.10	*DG*	67h
Cercopithecus mitis	11	7	4	0.54	0	0.35	0.11	0.95	0.54	0	0.35	0.11	*PG*	62
Colobus guereza	9	6	3	0.41	0.01	0.20	0.38	0.96	0.41	0.01	0.20	0.38	*PG*	62
Procolobus rufomitratus	13	7	6	0.35	0.01	0.29	0.35	0.95	0.35	0.01	0.29	0.35	*PG*	62,63f
Nasalis larvatus	17	9	8										*PG*	68-70i
Trachypithecus sp.	17	7	10	0.60	0	0.13	0.28	0.99	0.60	0	0.13	0.28	*PG*	71
Presbytis melalophos	2	1	1	0.28	0.02	0.19	0.50	0.99	0.28	0.02	0.19	0.50	*PG*	71
Alouatta sp.	32	13	19	0.61	0.02	0.33	0.05	0.95	0.61	0.02	0.33	0.05	*PG*	72j
Ateles geoffroyi	13	2	11	0.42	0.25	0.25	0.07	0.99	0.42	0.25	0.25	0.07	*S*	73-75f
Cebus apella	28	20	8	0.37	0	0.40	0.21	0.95	0.37	0	0.40	0.21	*PG*	76,77f
Total n	343	187	156											

b	Specimen	Discovery	Site
Undescribed	KNM-MV 4	1932	Mteitei Valley
	KNM-CA 409	1934	Chamtwara
	KNM-SO 1000	1966	Songhor
	KNM-SO 1001	1966	Songhor
	KNM-SO 31245	1996	Songhor
	KNM-SO 31246	1996	Songhor
	KNM-SO 1002	1966	Songhor
Ekembo heseloni	KNM KPS III C26[k]	1984	Rusinga
	KNM KPS III C28	1984	Rusinga
	KNM KPS VIII C27	1984	Rusinga
	KNM-RU 2036M	1951	Rusinga

[a] When combined locomotor proportions were not available, values for travel and feeding or for males and females were averaged, weighted by number of observations when available. Not adjusted to reflect minor differences in *Arb* between highly arboreal (*Arb* ≥ .95) species.
[b] Estimated at .99 in highly arboreal species for which terrestrial locomotion was not reported.
[c] *Arb* estimated based on ref. [78]; total proportions extrapolated.
[d] *Arb* estimated as slightly exceeding *G. beringei*; total proportions extrapolated.
[e] *Arb* calculated from ref. [79]; *Leap* estimated based on *P. abelii* and subtracted from horizontal clambering, classified here under suspension.
[f] Average of reported values.
[g] *Arb* from ref [80].
[h] *Arb* estimated, arboreal proportions extrapolated.
[i] *PG* inferred from general behavioural descriptions following refs [20,81].
[j] Average of values compiled for sampled species.
[k] KPS = Kaswanga Primate Site, Rusinga Island.

Body mass estimation

Body mass was estimated based on log-log OLS regression of sex-specific mean capitate volume against sex-specific mean body mass means. Body mass data for sampled individuals were compiled from museum records when available, supplemented by published data[87,88]. Body mass estimation studies frequently use measurements of articular surfaces as size proxies[82-86]; while certain postcranial articular surfaces have both theoretical and experimental foundations as body mass proxies (e.g., the opposing surfaces of the hip and tibiotalar joints), the proportional size of the capitate's various surfaces are variant within and between anthropoid species, whether in association with function, phylogeny, or idiosyncratically. Cross-validated prediction error (%SEE) calculated in the current extant sample for the surface areas of the Mc2, Mc3, hamate, and proximoradial facets in log-log OLS are 8.95, 6.32, 5.36, and 5.56, respectively, compared to 5.25 for capitate volume.

Male *Gorilla* was excluded from the model due to its extreme size relative to the fossil specimens. Smith[89,90] has recommended limiting the range of training set body masses to those similar to the experimental set; the remaining sex-specific great ape data were nevertheless included to account for possible scaling variation between anthropoid groups. "Classical calibration" was used to produce maximum likelihood body mass estimates for each fossil specimen based on regression of morphometric data against body mass[91,92]. The resulting equation has an R^2 value of 0.977 (intercept = -2.30, slope = 0.786). Although log transformation is common in scaling analyses to correct for heteroscedasticity and improve model fit[85], it has been suggested that log-log allometric regressions can be misleading[93,94]. We follow others[95] in judging the superior interpretability and transferability of this approach to outweigh any slight increase in

accuracy that may be afforded by suggested alternatives. Systematic underestimation resulting from detransformation of logged predictions into standard linear space was ameliorated by applying the quasi-maximum likelihood estimator correction factor[96]. Different correction factors tend to converge when representing less than about 10% of the detransformed values[97]; the one used here represented 1.1%.

Feature selection

Because the inclusion of redundant or irrelevant variables can cause important problems in multivariate analyses[98-100], models were built using only the shape variables found to covary in the extant sample with the relevant behavioural or taxonomic variable. Univariate analyses were performed with the size surrogate as a covariate to partially account for the influence of allometry. Covariance with locomotor proportions was tested using phylogenetic generalised least squares regression (PGLS)[101]. Covariance with positional classes was assessed using phylogenetic generalised linear mixed modelling (PGLMM)[102-105], with separate models fitted for the taxon means and individual observations of each shape variable. All PGLS and PGLMM analyses were repeated with hylobatids excluded to allow the influence of this group's high-leverage data to be considered when evaluating functional relationships. The utility of each morphometric in distinguishing among anthropoid superfamilies was assessed via multinomial logistic regression.

Shape variables not significantly distinguishing suspensory, knuckle-walking, or digitigrade anthropoids from the palmigrade reference class in any of the four PGLMM analyses (taxon means and individual observations, with and without hylobatids; Table S4) were excluded from positional classifiers (See Table S7b for list of included variables). Those distinguishing both hominoid and ceboid observations from cercopithecoids (Table S3a) were validated for inclusion in the taxonomic classifiers (See Table S9b for list of included variables). A metric's utility in reconstructing phylogenetic relationships is adversely affected by high allometric signal and benefited by high phylogenetic signal[106]. Therefore metrics included in hierarchical clustering models were limited to those with significant (non-zero) values of both λ[107] and K[108] (Table S3b), and that were not significantly allometric. The latter was measured via Spearman correlations between phylogenetic independent contrasts (PICs)[109] of each metric and the size surrogate, chosen for its robusticity against the sample's heteroscedastic size distribution (following ref [106]), as well as ease of interpretation (Table S2). All variables were mean-centred and scaled to a common standard deviation of one to prevent bias due to unit heterogeneity. Fossil shape variables were then centred and scaled according to the mean and standard deviation of the extant sample to ensure comparability. Phylogenetic information was taken from version 3 of the 10ktrees project[110] (Fig. S9c; tree available as separate download).

Table S2. Evaluation of allometric scaling in shape variables, assessed relative to capitate volume using Spearman correlations of phylogenetic independent contrasts. See Table 1 for variable definitions.

	Taxon means		Males		Females	
	rho	p	rho	p	rho	p
CpPx	0.11	0.58	0.08	0.71	0.16	0.42
CpSc	-0.25	0.22	-0.21	0.30	-0.01	0.96
CpLu	0.15	0.47	0.31	0.12	0.24	0.24
CpDn	0.07	0.73	-0.12	0.56	0.05	0.80
Cp3	-0.04	0.85	-0.10	0.63	0.29	0.14
CpHm	-0.37	0.06	-0.21	0.30	-0.27	0.18
Cp2	-0.18	0.36	-0.21	0.28	-0.33	0.09
Cp4	-0.24	0.23	-0.29	0.14	-0.20	0.32
Cp23A	-0.21	0.30	-0.09	0.67	-0.27	0.17
Cp3HmA	-0.07	0.72	-0.05	0.81	0.04	0.83
CpPxA	0.36	0.07	0.36	0.07	0.19	0.34
CpScA	-0.13	0.53	-0.05	0.82	-0.18	0.38
Cp3SD	0.11	0.57	-0.04	0.85	0.13	0.52
CpHmC	0.09	0.64	0.22	0.27	0.06	0.77
CpHP	-0.31	0.12	0.03	0.88	-0.27	0.18

Table S3. (a) Covariance of shape variables with Hominoidea and Platyrrhini relative to Cercopithecoidea. Reported results are from univariate ordinary least squares (OLS) regression of individual observations except where noted. (b) Phylogenetic signal estimated with Pagel's lambda and Blomberg's K.

**a**		Hominoidea				Platyrrhini				**b** Phylogenetic signal			
	R^2	b	p	OR[a]	p[a]	b	p	OR[a]	p[a]	λ	p	K	p
CpPx	0.19	-0.70	**0.00**	-3.47	**0.00**	-1.00	**0.00**	-1.53	**0.00**	0.88	**0.00**	0.42	**0.01**
CpSc	0.23	-1.26	**0.00**	-1.76	**0.00**	-0.59	**0.00**	-0.51	**0.01**	0.86	**0.00**	0.36	**0.00**
CpLu	0.11	0.36	**0.00**	-1.65	**0.00**	-0.59	**0.00**	-1.09	**0.00**	0.97	**0.00**	0.65	**0.00**
CpDn	0.05	-0.43	**0.00**	-0.27	0.10	-0.59	**0.00**	-0.66	**0.00**	0.99	**0.00**	0.57	**0.00**
Cp3	0.24	0.44	**0.00**	0.01	0.96	1.24	**0.00**	1.69	**0.00**	0.91	**0.00**	0.52	**0.00**
CpHm	0.21	-0.29	**0.01**	0.34	0.12	1.09	**0.00**	1.51	**0.00**	0.71	**0.00**	0.48	**0.00**
Cp2	0.47	-1.57	**0.00**	-3.13	**0.00**	-0.69	**0.00**	-0.88	**0.00**	0.99	**0.00**	1.12	**0.00**
Cp4	0.35	-0.43	**0.00**	1.79	**0.00**	1.13	**0.00**	1.81	**0.00**	1.01	**0.00**	1.96	**0.00**
Cp23A	0.15	0.41	**0.00**	1.26	**0.00**	-0.80	**0.00**	-1.06	**0.00**	0.97	**0.00**	0.65	**0.00**
Cp3HmA	0.09	-0.67	**0.00**	-2.40	**0.00**	-0.47	**0.00**	-0.05	0.81	0.76	**0.02**	0.34	**0.02**
CpPxA	0.14	-0.79	**0.00**	-3.31	**0.00**	-0.51	**0.00**	-0.55	**0.01**	0.98	**0.00**	0.91	**0.00**
CpScA	0.39	-1.00	**0.00**	-2.98	**0.00**	0.94	**0.00**	2.47	**0.00**	1.00	**0.00**	2.09	**0.00**
Cp3SD	0.18	0.06	0.60	-0.96	**0.00**	-1.08	**0.00**	-2.00	**0.00**	0.82	**0.02**	0.52	**0.00**
CpHmC	0.30	-0.67	**0.00**	-6.53	**0.00**	-1.37	**0.00**	-3.23	**0.00**	1.01	**0.00**	1.32	**0.00**
CpHP	0.17	-0.89	**0.00**	-0.45	**0.02**	-0.88	**0.00**	-0.72	**0.00**	0.88	**0.00**	0.50	**0.00**

[a] Based on multinomial logistic regression with size (log-transformed sum carpal volume) as a covariate. OR, odds ratio (log scale).

Table S4. Covariance of shape variables with positional classes relative to palmigrade (*PG*) reference class. Reported results are from univariate phylogenetic generalised least squares (PGLS) regression of taxon means except where noted.

	R^2	λ	*DG*						*KW*						*S*					
			b	p	OR[a]	p[a]	OR[b]	p[b]	b	p	OR[a]	p[a]	OR[b]	p[b]	b	p	OR[a]	p[a]	OR[b]	p[b]
CpPx	0.18	0.82	0.12	0.77	1.44	0.25	1.17	0.21	0.63	0.39	-0.25	0.82	-0.24	0.80	-0.64	0.27	-2.45	**0.04**	-1.15	0.22
CpSc	0.20	0.71	0.06	0.88	1.82	0.17	0.90	0.27	-0.38	0.57	-0.24	0.83	0.87	0.31	-1.16	**0.04**	-3.43	**0.01**	-1.56	0.06
CpLu	0.06	0.96	0.16	0.72	0.61	0.65	0.45	0.63	1.10	0.28	-0.42	0.70	-0.73	0.44	0.20	0.79	-0.52	0.62	0.27	0.77
CpDn	0.03	0.99	0.32	0.50	1.62	0.14	0.89	0.25	-0.42	0.72	0.13	0.90	0.08	0.91	-0.37	0.67	-0.23	0.86	-0.56	0.47
Cp3	0.22	0.77	-0.53	0.26	-1.31	0.24	-1.13	0.24	1.21	0.13	0.86	0.47	1.26	0.21	-0.24	0.70	-0.89	0.37	-0.33	0.73
CpHm	0.05	0.62	-0.15	0.78	-0.36	0.76	-0.19	0.83	-0.70	0.36	-0.40	0.75	0.17	0.83	-0.04	0.95	0.09	0.93	0.08	0.89
Cp2	0.30	0.98	0.46	0.11	2.13	0.10	2.24	**0.02**	-1.66	**0.02**	-1.94	0.16	-1.25	0.21	-1.13	**0.03**	-3.16	**0.02**	-1.48	0.13
Cp4	0.16	1.00	-0.17	0.55	-0.23	0.88	-0.01	0.99	-1.43	0.06	-2.48	0.10	-2.55	**0.03**	-0.38	0.49	1.31	0.28	0.87	0.40
Cp23A	0.26	0.88	-0.83	0.06	-1.83	0.13	-1.07	0.23	-0.50	0.54	-1.09	0.39	-1.31	0.17	0.71	0.26	1.48	0.19	0.72	0.41
Cp3HmA	0.08	0.71	0.64	0.25	1.34	0.21	1.13	0.24	0.57	0.51	0.23	0.86	-0.11	0.90	-0.04	0.95	-0.71	0.51	0.11	0.89
CpPxA	0.67	0.81	-0.23	0.33	-0.74	0.55	0.22	0.83	0.28	0.51	1.39	0.34	1.15	0.27	-1.69	**0.00**	-4.39	**0.00**	-3.01	**0.00**
CpScA	0.25	1.00	-0.37	0.13	-1.25	0.24	-0.72	0.46	0.14	0.82	0.49	0.71	1.49	0.15	-0.82	0.08	-2.60	**0.02**	-1.39	0.16
Cp3SD	0.06	0.78	-0.14	0.79	0.76	0.49	0.56	0.56	0.89	0.34	0.40	0.76	-0.18	0.82	0.02	0.98	-0.65	0.53	-0.50	0.59
CpHmC	0.39	1.00	0.23	0.37	2.03	0.15	2.32	0.06	0.18	0.78	0.75	0.65	-0.39	0.73	-1.36	**0.01**	-3.50	**0.00**	-2.61	**0.02**
CpHP	0.50	0.00	0.73	0.15	1.38	0.22	0.96	0.29	-1.42	**0.00**	-2.27	0.08	-1.43	0.12	0.01	0.99	0.18	0.85	-0.20	0.82
Without hylobatids																				
CpPx	0.05	0.47	0.40	0.51	1.55	0.16	0.90	0.32	0.38	0.61	0.11	0.96	-0.12	0.90	-0.23	0.75	-1.01	0.35	-0.82	0.36
CpSc	0.17	0.73	0.05	0.91	1.85	0.16	0.63	0.44	-0.34	0.64	-0.23	0.85	0.82	0.32	-1.22	0.07	-2.96	**0.01**	-1.57	0.05
CpLu	0.16	0.77	0.15	0.75	0.07	0.94	0.13	0.89	1.01	0.21	0.16	0.91	-0.36	0.69	1.29	0.07	2.02	0.12	0.84	0.36
CpDn	0.10	0.97	0.37	0.38	2.33	**0.05**	1.27	0.11	-0.24	0.81	0.12	0.91	-0.51	0.53	-0.83	0.29	-2.25	0.08	-1.37	0.09
Cp3	0.20	0.60	-0.68	0.19	-1.82	0.11	-2.25	**0.03**	1.13	0.12	1.45	0.18	2.27	**0.03**	0.23	0.72	0.65	0.54	0.89	0.38
CpHm	0.03	0.68	-0.13	0.80	-0.52	0.64	-0.46	0.60	-0.60	0.45	-0.46	0.71	0.27	0.77	-0.13	0.86	0.52	0.63	0.31	0.75
Cp2	0.30	0.99	0.44	0.14	2.21	0.08	1.96	0.06	-1.57	**0.04**	-1.95	0.15	-1.29	0.23	-1.10	0.06	-2.50	0.06	-1.45	0.16
Cp4	0.29	1.00	-0.17	0.40	0.08	0.93	0.41	0.70	-1.35	**0.02**	-2.89	0.06	-2.71	**0.02**	-0.84	**0.05**	0.00	1.00	0.39	0.71
Cp23A	0.21	0.90	-1.02	0.05	-1.83	0.07	-0.70	0.43	-0.51	0.62	-1.28	0.24	-1.26	0.16	0.44	0.60	0.28	0.80	0.41	0.63
Cp3HmA	0.16	0.00	1.05	0.12	1.35	0.14	0.76	0.39	0.36	0.46	0.62	0.53	0.16	0.86	0.86	0.20	1.02	0.27	0.63	0.47
CpPxA	0.74	0.00	-0.65	0.08	-1.01	0.33	-0.05	0.95	0.30	0.27	1.15	0.37	0.79	0.41	-2.39	**0.00**	-3.32	**0.00**	-2.38	**0.02**
CpScA	0.19	1.00	-0.50	0.10	-1.86	0.09	-1.44	0.13	0.00	1.00	0.56	0.62	1.69	0.10	-0.64	0.28	-1.01	0.36	-0.51	0.59
Cp3SD	0.07	0.97	-0.38	0.43	0.78	0.44	0.51	0.60	0.95	0.41	0.48	0.70	-0.15	0.87	0.55	0.54	-0.31	0.79	-0.36	0.72
CpHmC	0.36	0.94	0.40	0.25	2.30	0.07	1.82	0.09	0.04	0.96	1.06	0.42	-0.18	0.87	-1.57	**0.02**	-2.36	**0.03**	-1.90	0.08
CpHP	0.60	0.00	0.75	0.10	1.65	0.14	1.17	0.19	-1.46	**0.00**	-2.79	**0.03**	-2.00	**0.03**	-0.68	0.14	-1.08	0.33	-0.83	0.35

[a] Based on PGLMM regression of taxon means with size as a covariate. OR, odds ratio (log scale).

[b] Based on PGLMM analysis of all observations with size as a covariate.

Table S5. Relationships between shape variables and selected locomotor proportions based on PGLS regression

	**a** *QuadA*						**b** *Quad*						**c** *SuspA*					
	R^2	λ	b	p	b^a	p^a	R^2	λ	b	p	b^a	p^a	R^2	λ	b	p	b^a	p^a
CpPx	0.26	0.56	0.49	**0.02**	0.78	**0.00**	0.53	0.00	0.72	**0.00**	0.67	**0.00**	0.14	1.00	-0.26	0.08	-0.27	0.10
CpSc	0.11	0.58	0.33	0.14	0.32	0.17	0.04	1.00	0.19	0.39	0.71	**0.00**	0.07	1.00	-0.19	0.24	-0.20	0.23
CpLu	0.10	0.79	0.30	0.16	0.46	0.10	0.02	1.00	0.14	0.48	-0.06	0.74	0.05	1.00	-0.14	0.34	-0.14	0.38
CpDn	0.10	0.88	0.27	0.15	0.28	0.14	0.06	1.00	0.20	0.26	0.21	0.16	0.00	1.00	-0.02	0.89	-0.02	0.88
Cp3	0.05	0.72	0.20	0.34	0.19	0.38	0.03	1.00	0.13	0.48	0.05	0.76	0.06	1.00	-0.14	0.29	-0.13	0.32
CpHm	0.11	0.86	-0.30	0.13	-0.30	0.16	0.01	1.00	-0.08	0.64	-0.02	0.91	0.08	1.00	0.14	0.21	0.14	0.23
Cp2	0.02	0.79	0.17	0.52	0.25	0.39	0.00	1.00	0.03	0.90	0.23	0.35	0.02	1.00	-0.13	0.51	-0.16	0.44
Cp4	0.02	0.80	-0.17	0.51	-0.19	0.59	0.05	1.00	-0.29	0.30	0.13	0.68	0.05	1.00	0.20	0.32	0.24	0.35
Cp23A	0.17	0.53	-0.40	0.05	-0.46	**0.03**	0.19	0.87	-0.46	**0.04**	-0.20	0.32	0.21	1.00	0.31	**0.03**	0.31	**0.04**
Cp3HmA	0.03	0.77	0.14	0.44	0.13	0.51	0.00	1.00	-0.01	0.96	-0.05	0.69	0.04	1.00	-0.09	0.40	-0.08	0.43
CpPxA	0.34	0.48	0.56	**0.00**	0.71	**0.00**	0.42	0.59	0.70	**0.00**	0.55	**0.01**	0.84	0.58	-0.86	**0.00**	-0.96	**0.00**
CpScA	0.19	0.57	0.49	**0.04**	0.49	0.05	0.02	1.00	0.21	0.53	0.29	0.32	0.33	1.00	-0.60	**0.01**	-0.62	**0.01**
Cp3SD	0.31	0.85	0.55	**0.01**	0.59	**0.01**	0.19	1.00	0.43	**0.04**	0.31	0.11	0.06	1.00	-0.18	0.25	-0.17	0.29
CpHmC	0.29	0.65	0.54	**0.01**	0.77	**0.00**	0.39	0.72	0.69	**0.00**	0.36	0.13	0.43	1.00	-0.54	**0.00**	-0.63	**0.00**
CpHP	0.00	0.82	0.06	0.76	0.08	0.71	0.00	1.00	0.04	0.80	0.05	0.72	0.01	1.00	0.06	0.64	0.06	0.65
Without hylobatids																		
CpPx	0.02	0.53	0.16	0.53	0.35	0.12	0.23	0.00	0.48	**0.04**	0.02	0.93	0.00	1.00	0.00	0.98	-0.02	0.90
CpSc	0.43	0.60	0.65	**0.00**	0.33	0.18	0.02	1.00	0.16	0.54	0.63	**0.01**	0.52	0.00	-0.72	**0.00**	-0.63	**0.01**
CpLu	0.06	0.72	-0.28	0.30	0.12	0.76	0.00	1.00	-0.06	0.80	-0.20	0.37	0.03	1.00	0.13	0.49	0.08	0.69
CpDn	0.23	0.00	0.47	**0.04**	0.60	**0.00**	0.27	1.00	0.48	**0.02**	0.48	**0.01**	0.15	1.00	-0.28	0.10	-0.29	0.09
Cp3	0.01	0.51	-0.07	0.77	-0.10	0.64	0.00	1.00	0.01	0.96	-0.03	0.88	0.00	1.00	0.00	0.99	-0.02	0.90
CpHm	0.05	0.52	-0.18	0.37	-0.28	0.31	0.00	1.00	-0.06	0.78	-0.02	0.90	0.08	1.00	0.19	0.24	0.21	0.19
Cp2	0.06	0.47	0.23	0.32	0.22	0.50	0.00	1.00	0.03	0.93	0.28	0.40	0.04	1.00	-0.21	0.43	-0.13	0.66
Cp4	0.01	0.48	0.14	0.62	0.07	0.86	0.00	1.00	-0.11	0.84	0.50	0.37	0.06	1.00	-0.39	0.33	-0.23	0.62
Cp23A	0.16	0.00	-0.40	0.09	-0.07	0.75	0.00	1.00	-0.05	0.82	-0.03	0.89	0.06	1.00	0.16	0.33	0.16	0.30
Cp3HmA	0.19	0.77	0.49	0.06	-0.14	0.51	0.03	1.00	-0.10	0.45	-0.09	0.46	0.01	1.00	0.03	0.74	0.04	0.71
CpPxA	0.07	0.59	0.30	0.27	0.23	0.28	0.17	0.62	0.41	0.08	0.42	0.06	0.62	0.65	-0.70	**0.00**	-0.75	**0.00**
CpScA	0.30	0.00	0.55	**0.02**	-0.10	0.73	0.02	1.00	-0.22	0.57	0.06	0.87	0.10	1.00	-0.40	0.18	-0.33	0.32
Cp3SD	0.01	0.62	-0.13	0.65	0.48	**0.04**	0.11	1.00	0.36	0.16	0.29	0.23	0.00	1.00	0.01	0.98	-0.03	0.88
CpHmC	0.16	0.38	-0.37	0.09	0.38	0.08	0.20	0.71	0.55	0.05	0.28	0.31	0.15	1.00	-0.37	0.10	-0.48	**0.04**
CpHP	0.44	0.00	0.66	**0.00**	0.40	0.07	0.05	1.00	0.19	0.36	0.19	0.33	0.02	1.00	-0.09	0.57	-0.10	0.57

	**d** *Susp*						**e** *ClimbA*						**f** *Climb*					
	R^2	λ	b	p	b^a	p^a	R^2	λ	b	p	b^a	p^a	R^2	λ	b	p	b^a	p^a
CpPx	0.23	1.00	-0.34	**0.02**	-0.34	**0.03**	0.13	0.71	0.36	0.10	0.23	0.31	0.11	0.00	-0.33	0.14	-0.18	0.40
CpSc	0.17	1.00	-0.30	0.06	-0.32	**0.05**	0.05	0.80	0.24	0.30	0.30	0.14	0.04	0.34	-0.23	0.36	-0.40	0.06
CpLu	0.05	1.00	-0.14	0.32	-0.13	0.42	0.05	0.73	0.21	0.33	-0.04	0.88	0.07	0.00	-0.26	0.24	0.21	0.49

CpDn	0.00	1.00	0.01	0.91	0.01	0.93	0.01	0.81	-0.10	0.61	-0.02	0.92	0.00	0.30	-0.06	0.78	-0.14	0.49
Cp3	0.08	1.00	-0.16	0.21	-0.15	0.26	0.16	0.79	0.37	0.07	0.29	0.09	0.04	0.32	-0.20	0.40	-0.05	0.81
CpHm	0.02	1.00	0.08	0.49	0.07	0.55	0.00	0.79	-0.05	0.81	0.16	0.48	0.07	0.00	0.26	0.23	-0.07	0.78
Cp2	0.01	1.00	-0.09	0.66	-0.13	0.53	0.02	0.78	-0.15	0.56	-0.08	0.74	0.02	0.00	0.16	0.48	-0.32	0.24
Cp4	0.07	1.00	0.23	0.25	0.25	0.33	0.18	0.70	-0.48	**0.05**	-0.35	0.30	0.17	0.00	0.41	0.06	-0.79	0.05
Cp23A	0.19	1.00	0.30	**0.04**	0.29	0.06	0.19	0.78	-0.45	**0.04**	-0.34	**0.04**	0.04	0.42	0.21	0.39	0.13	0.53
Cp3HmA	0.03	1.00	-0.09	0.41	-0.08	0.46	0.04	0.75	0.16	0.39	0.10	0.59	0.01	0.00	-0.10	0.65	0.04	0.84
CpPxA	0.91	0.00	-0.95	**0.00**	-0.96	**0.00**	0.24	0.81	0.54	**0.02**	0.43	**0.04**	0.07	0.18	-0.28	0.23	-0.23	0.25
CpScA	0.37	1.00	-0.65	**0.00**	-0.67	**0.00**	0.01	0.80	0.14	0.63	0.16	0.40	0.01	0.42	-0.15	0.61	-0.17	0.42
Cp3SD	0.05	1.00	-0.15	0.32	-0.14	0.40	0.00	0.79	0.03	0.89	-0.02	0.92	0.16	0.00	-0.40	0.06	-0.21	0.37
CpHmC	0.46	1.00	-0.57	**0.00**	-0.59	**0.00**	0.16	0.69	0.41	0.07	0.29	0.19	0.11	0.00	-0.34	0.12	-0.19	0.38
CpHP	0.03	1.00	0.09	0.46	0.09	0.47	0.07	0.72	-0.24	0.23	-0.29	0.12	0.05	0.00	0.21	0.34	0.03	0.91
Without hylobatids																		
CpPx	0.03	1.00	-0.11	0.47	-0.12	0.43	0.01	0.75	0.10	0.64	0.06	0.80	0.21	0.00	-0.46	**0.05**	-0.32	0.16
CpSc	0.17	1.00	-0.36	0.07	-0.34	0.11	0.04	0.79	0.19	0.40	0.26	0.26	0.03	0.34	-0.18	0.49	-0.52	**0.03**
CpLu	0.03	1.00	0.13	0.48	0.10	0.63	0.01	0.79	-0.11	0.66	-0.52	0.12	0.09	0.00	-0.30	0.22	0.39	0.33
CpDn	0.09	1.00	-0.22	0.21	-0.22	0.21	0.00	0.78	-0.04	0.84	0.06	0.78	0.04	0.37	-0.21	0.39	-0.34	0.11
Cp3	0.00	1.00	-0.04	0.81	-0.05	0.75	0.10	0.81	0.26	0.18	0.23	0.27	0.02	0.33	-0.16	0.53	-0.05	0.84
CpHm	0.01	1.00	0.07	0.65	0.09	0.60	0.00	0.77	0.04	0.86	0.08	0.75	0.08	0.00	0.29	0.23	-0.10	0.65
Cp2	0.02	1.00	-0.14	0.60	-0.08	0.79	0.05	0.70	-0.25	0.34	-0.29	0.32	0.03	0.00	0.17	0.48	-0.47	0.15
Cp4	0.03	1.00	-0.30	0.46	-0.18	0.71	0.04	0.71	-0.26	0.42	-0.05	0.90	0.23	0.00	0.48	**0.04**	-0.45	0.43
Cp23A	0.04	1.00	0.13	0.41	0.14	0.39	0.14	0.80	-0.31	0.12	-0.38	**0.05**	0.03	0.44	0.16	0.51	0.19	0.40
Cp3HmA	0.01	1.00	0.04	0.69	0.04	0.67	0.00	0.77	-0.05	0.78	-0.05	0.79	0.01	0.00	-0.08	0.73	0.06	0.63
CpPxA	0.73	0.00	-0.85	**0.00**	-0.83	**0.00**	0.16	0.83	0.33	0.09	0.33	0.09	0.13	0.00	-0.36	0.13	-0.43	**0.04**
CpScA	0.15	1.00	-0.48	0.11	-0.46	0.17	0.02	0.72	-0.16	0.57	-0.06	0.83	0.02	0.00	0.15	0.54	-0.48	0.13
Cp3SD	0.00	1.00	0.06	0.78	0.03	0.88	0.04	0.83	-0.20	0.39	-0.19	0.43	0.18	0.00	-0.42	0.07	-0.23	0.35
CpHmC	0.19	1.00	-0.41	0.07	-0.44	0.05	0.06	0.71	0.25	0.31	0.21	0.38	0.18	0.00	-0.42	0.07	-0.28	0.22
CpHP	0.00	1.00	-0.03	0.85	-0.03	0.85	0.09	0.72	-0.27	0.20	-0.33	0.11	0.02	0.00	0.14	0.56	-0.20	0.34
**g**	*LeapA*						**h** *Leap*						**i** *Arb*					
	R^2	λ	b	p	b^a	p^a	R^2	λ	b	p	b^a	p^a	R^2	λ	b	p	b^a	p^a
CpPx	0.01	1.00	-0.09	0.65	0.01	0.95	0.02	1.00	-0.14	0.49	-0.01	0.95	0.20	0.52	-0.48	**0.04**	-0.30	0.09
CpSc	0.06	1.00	0.22	0.27	0.17	0.38	0.03	1.00	0.16	0.46	0.09	0.64	0.01	1.00	-0.12	0.65	-0.48	**0.00**
CpLu	0.08	1.00	-0.23	0.19	-0.12	0.53	0.08	1.00	-0.24	0.19	-0.08	0.66	0.01	1.00	-0.09	0.68	0.22	0.24
CpDn	0.12	1.00	-0.24	0.11	-0.25	0.08	0.14	1.00	-0.28	0.08	-0.30	**0.04**	0.04	1.00	-0.18	0.35	-0.21	0.17
Cp3	0.01	1.00	-0.06	0.72	0.00	0.98	0.00	1.00	0.02	0.90	0.10	0.53	0.00	1.00	-0.03	0.88	0.09	0.58
CpHm	0.09	1.00	0.19	0.18	0.15	0.26	0.05	1.00	0.15	0.32	0.10	0.46	0.00	1.00	-0.01	0.97	-0.10	0.50
Cp2	0.02	1.00	0.15	0.53	0.03	0.89	0.00	1.00	0.01	0.96	-0.16	0.49	0.01	1.00	-0.13	0.68	-0.42	0.08
Cp4	0.13	1.00	0.40	0.10	0.22	0.46	0.10	1.00	0.38	0.15	0.06	0.83	0.29	0.00	0.54	**0.01**	-0.48	0.10
Cp23A	0.00	1.00	0.00	0.99	-0.07	0.71	0.00	1.00	0.02	0.92	-0.07	0.70	0.21	0.74	0.50	**0.03**	0.39	**0.01**
Cp3HmA	0.00	1.00	0.00	0.99	0.03	0.80	0.00	1.00	-0.03	0.84	0.01	0.93	0.00	1.00	0.01	0.96	0.07	0.57

CpPxA	0.00	1.00	0.00	0.99	0.07	0.75	0.00	1.00	0.07	0.76	0.17	0.43	0.12	0.69	-0.39	0.12	-0.32	0.05
CpScA	0.18	1.00	0.56	**0.05**	0.51	0.06	0.17	1.00	0.57	0.06	0.51	0.06	0.00	1.00	0.07	0.85	-0.04	0.90
Cp3SD	0.15	1.00	-0.33	0.08	-0.25	0.17	0.15	1.00	-0.36	0.07	-0.25	0.18	0.17	0.57	-0.43	0.05	-0.12	0.56
CpHmC	0.00	1.00	-0.02	0.94	0.12	0.59	0.01	1.00	-0.11	0.66	0.07	0.77	0.28	0.56	-0.58	**0.01**	-0.37	**0.03**
CpHP	0.06	1.00	-0.16	0.27	-0.17	0.22	0.06	1.00	-0.17	0.28	-0.18	0.20	0.01	1.00	-0.07	0.70	-0.09	0.55
Without hylobatids																		
CpPx	0.00	1.00	0.00	0.99	0.02	0.89	0.01	1.00	-0.04	0.76	-0.02	0.90	0.15	0.37	-0.39	0.11	-0.27	0.18
CpSc	0.05	1.00	0.17	0.35	0.11	0.53	0.02	1.00	0.12	0.56	0.03	0.88	0.01	1.00	-0.08	0.75	-0.50	**0.01**
CpLu	0.04	1.00	-0.13	0.42	-0.06	0.70	0.04	1.00	-0.14	0.41	-0.05	0.79	0.00	1.00	0.01	0.96	0.21	0.26
CpDn	0.17	1.00	-0.26	0.08	-0.26	0.06	0.22	1.00	-0.32	**0.04**	-0.32	**0.02**	0.12	1.00	-0.32	0.14	-0.42	**0.01**
Cp3	0.00	1.00	-0.03	0.80	-0.01	0.92	0.01	1.00	0.05	0.73	0.08	0.54	0.00	1.00	0.04	0.85	0.09	0.55
CpHm	0.08	1.00	0.17	0.24	0.15	0.28	0.04	1.00	0.13	0.41	0.10	0.49	0.00	1.00	-0.03	0.89	-0.08	0.62
Cp2	0.07	1.00	0.24	0.28	0.14	0.56	0.01	1.00	0.10	0.70	-0.09	0.71	0.01	1.00	-0.13	0.71	-0.51	0.05
Cp4	0.02	1.00	0.18	0.61	-0.13	0.74	0.01	1.00	0.15	0.71	-0.33	0.41	0.25	0.00	0.50	**0.03**	-0.72	0.11
Cp23A	0.00	1.00	0.02	0.87	0.01	0.93	0.00	1.00	0.03	0.85	0.01	0.93	0.15	0.74	0.36	0.11	0.44	**0.01**
Cp3HmA	0.01	1.00	0.03	0.71	0.03	0.75	0.00	1.00	0.01	0.91	0.00	0.97	0.01	1.00	0.05	0.70	0.04	0.72
CpPxA	0.01	1.00	0.06	0.68	0.04	0.76	0.04	1.00	0.14	0.40	0.11	0.45	0.00	1.00	0.01	0.97	-0.32	0.07
CpScA	0.36	1.00	0.65	**0.01**	0.59	**0.02**	0.34	1.00	0.69	**0.01**	0.55	**0.04**	0.05	1.00	0.35	0.37	-0.07	0.84
Cp3SD	0.16	1.00	-0.30	0.09	-0.26	0.13	0.16	1.00	-0.32	0.09	-0.27	0.13	0.11	0.51	-0.35	0.16	-0.13	0.54
CpHmC	0.00	1.00	-0.04	0.84	-0.01	0.97	0.02	1.00	-0.14	0.53	-0.09	0.65	0.26	0.45	-0.57	**0.02**	-0.38	**0.05**
CpHP	0.01	1.00	-0.06	0.69	-0.06	0.68	0.02	1.00	-0.08	0.62	-0.08	0.58	0.03	1.00	-0.15	0.47	-0.15	0.36

[a] Based on PGLS model with size as a covariate.

Systematic analyses

The procedures used for the positional classifiers were also used to build models to classify the fossils into one of the three anthropoid superfamilies (Fig. S8). The taxonomic DFA model distinguishes hominoids from ceboids and, to a lesser extent, cercopithecoids along the first axis, which is dominated by the orientation of the scaphoid/central facet (CpScA) and the relative size of the Mc4 facet (Cp4; Table S9b). The second axis separates ceboids from most cercopithecoids, while also contributing to distinguishing hominoids from cercopithecoids. Values along the second axis are most strongly associated with a larger and more proximally oriented proximoradial surface (CpPx, CpPxA) and a larger and more distally oriented Mc2 surface (Cp2, Cp23A). In the *glmnet* model, platyrrhines are most strongly distinguished from other anthropoids by their large Mc4 facets (Cp4) and dorsally-oriented centrale facets (CpScA), and cercopithecoids by their large proximoradial facets (CpPx) and hamate facet concavity (CpHmC). Hominoids are meanwhile best distinguished by small Mc2 and Mc4 facets (Cp2, Cp4) and radially-oriented scaphoid/centrale facets (CpScA). Both models were effective in classifying the extant specimens, with balanced accuracy exceeding 95% (Table S9a).

BioNJ[111] was used to further explore the phylogenetic affinities of the fossil specimens. This and other neighbour-joining algorithms[112,113] improve on standard agglomerative methods (e.g., UPGMA) by joining the pair of tips that minimizes the branch lengths of the entire tree at each stage of clustering. BioNJ adds an additional approximation of distance variance and covariance, accounting somewhat for non-independence due to shared evolutionary history and reducing statistical noise[111,114,115]. Trees were outgroup-rooted using *Cebus*, providing a basis for approximating trait polarity and yielding trees that depict estimated phylogenetic, rather than only phenetic, relationships. Individual hierarchical analyses were carried out for each fossil specimen; their relationships to extant specimens are presented here in two dendrograms best preserving the phylogenetic placement of the fossils in individual analyses (Fig. S9).

Computational details

All analyses were done in R v3.4[116]. PGLS was done with *caper*[117]; PGLMMs were fitted with *MCMCglmm*[103]; two-block PLS was done using code from *Morpho*[118] and *geomorph*[119]; DFA was done with *MASS*[120]. Cross-validation of DFA and GLM models incorporated code taken from *ipred*[121]; AICc model selection was done with *MuMIn*[122]; *glmnet*[123] was used via wrapper functions in *caret*[124]; the latter package was also used for missing data imputation (function “bagImpute”). %SEE was calculated with *MASSTIMATE*[125]; Spearman correlations were done with *Hmisc*[126]; Tukey tests were done with *multcomp*[127]. *ape*[128] was used for hierarchical clustering, calculating PICs, and customizing and visualizing phylogenetic trees, the latter with the help of *phyclust*[129]. DFA and PLS visualizations were done in *ggplot2*[130]

SI results

Morphological descriptions

The Tinderet specimens are described in comparison with the Rusinga specimens, and with emphasis on functionally or taxonomically informative traits. These include those

identified to covary with locomotor proportions, positional groups, or extant superfamilies (Tables S3a, S4, and S5), as well as qualitative traits.

KNM-MV 4 This right capitate is the smallest of the fossil sample (see Table 3), similar in size to the average of sampled blue monkeys (*C. mitis*). It is well preserved and complete save for abrasion of the lateral approximately two-thirds of the dorsodistal margin. The capitate head is narrow both dorsopalmarly and mediolaterally, condyloid, and pronated substantially relative to the body, the latter resulting in the proximal region of the hamate facet facing somewhat dorsally. Distally, ligament notches are absent laterally and medially, allowing the Mc2 and Mc4 facets to run uninterrupted along the dorsopalmar entirety of these margins, a condition unique among the fossil sample with the possible exception of KNM-SO 31246 (see below). The palmar portion of the Mc4 facet widens and tilts medially, increasing in degree as it approaches its palmar extent. The Mc2 and Mc4 facets are separated by a wide and topographically mild articulation for the Mc3. In these features, this specimen is most reminiscent of *Cebus* of the extant sample.

In other features, KNM-MV 4 more closely resembles its sampled Miocene contemporaries. It lacks the lateral expansion of the distal portion of the body that characterizes the platyrrhines in the sample, and the distal portion of the hamate facet is oriented palmarly relative to the proximal portion. The topography of the Mc3 articular surface follows the basic pattern of the other fossils of the sample: the distal-most extent of the dorsal Mc3 margin occurs a short distance from its lateral extent, and the dorsal portion of the surface lateral to this point withdraws proximally before abutting the Mc2 facet. The remainder of the Mc3 surface is gently concave dorsopalmarly, and the proximal half slopes distally toward the raised margin it shares with the palmar portion of the Mc2 facet, just distal to a rugose, palmarly projecting ridge for attachment of palmar intercarpal ligaments. However, this topography is generally less pronounced, resulting in a surface deviating only slightly from planar.

KNM-CA 409 The Chamtwara specimen is a nearly complete, right capitate, similar in size to the largest patas and colobus monkeys of the sample. The centrale facet suffers from minor weathering, and small portions are abraded along the bone's dorsodistal margin and along the distal extent of its proximal surface both palmarly and dorsally.

This specimen resembles those from Rusinga in much of its morphology. The midline of the Mc2 facet is constricted proximally, likely due to encroachment by the capitotrapezoid interosseous ligament. The dorsal extent of this articular surface is expanded distally where it abuts the dorsodistal margin of the bone, and its wide palmar portion projects distally where it meets the reciprocally angled Mc3 facet to form a raised ridge along their shared palmar border. The palmar portion of the Mc3 facet is mildly concave both proximodistally and mediolaterally, while the dorsolateral portion is moderately withdrawn proximally. A lateral projection along the dorsal margin of the distal hamate facet is also evident, which serves to orient the distal portion of the hamate facet somewhat palmarly. This palmar orientation is common in extant apes, but the angulation that clearly delineates this portion of the hamate facet from the medially-oriented remainder has previously been identified exclusively in *E. heseloni*, in which this portion rides along the dorsum of the hamate[131]. This angulation is not quite as

pronounced in KNM-CA 409 (Fig. S5), but it is nevertheless clearly distinguished from the remainder of the hamate facet.

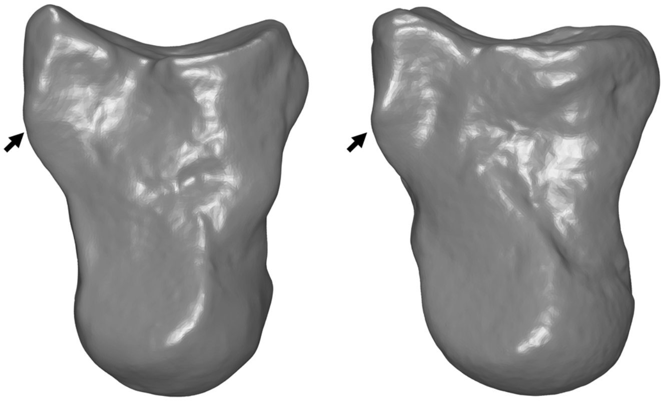

Fig. S5. Comparison of dorsodistal lip morphology in fossil specimens.

More proximally, the morphology of this specimen departs somewhat from that of the Rusinga specimens. While its most proximal point occurs palmarly relative to the other Tinderet specimens, it is positioned dorsally relative to each of the Rusinga specimens. This point is also lateral to the midline, and the most proximal surface is moderately angular in palmar or dorsal view rather than smoothly curved, resulting in a clear delineation between the lunate and centrale facets. The capitate head is not visibly pronated relative to the body, and resembles a quarter sphere in proximal view, as the lunate facet faces dorsally rather than dorsolaterally as in most others of the fossil sample (Fig. S2, proximal view). The palmar extent of the centrale facet roughly matches that of the contralateral hamate facet, whereas in each of the Rusinga specimens the centrale articulation extends palmarly to a substantial degree, even accounting for the head’s pronated position. The lunate facet terminates more proximally on the dorsum of the bone relative to all others of the fossil sample. These features indicate a potential difference in midcarpal mobility, with KNM-CA 409 perhaps having facilitated a relatively limited range of extension and supination.

KNM-SO 1000 This left capitate is slightly larger than KNM-MV 4, of a size with sampled lutungs or smaller spider monkeys. It suffers from weathering over most of its surface, and a small chip is missing from the proximopalmar corner of the hamate facet, but it shows no sign of plastic deformation. It is extremely gracile, with an apparent fineness ratio (i.e., proximodistal length relative to mediolateral or dorsopalmar width) exceeding that of most hylobatids. The hamate surface is very long and dorsopalmarly narrow, with only mild concavity. The head is narrow both dorsopalmarly and especially mediolaterally and is not pronated relative to the body of the capitate, orienting its hamate facet directly medially. The proximal facet remains parallel with the dorsal nonarticular region for some distance near its medial margin with the hamate facet before angling sharply toward its palmar margin, rather than curving toward it immediately. The centrale facet is therefore more radially oriented, and is also palmarly

expanded relative to the hamate facet, which, given cooperative soft tissue, would allow the centrale to translate farther palmarly, enhancing the range of mid-carpal supination[132]. The centrale facet remains convex at its distal extent, terminating in the neck region proximal to where the body angles laterally in the region of the trapezoid articulation. This condition occurs often in *Pongo* and *Gorilla*, but only rarely in hylobatids and *Pan* (Fig. S7b).

Distally, the Mc3 surface is mediolaterally narrow, and winnows progressively before coming to a palmolateral point. The entire facet is uniformly concave along its medial border; laterally, it shares the withdrawn dorsal portion and distally projecting palmar portion with its contemporaries, although the latter feature may be most pronounced in this specimen. The latter condition displaces the palmar Mc2 articulation more distally, resembling an inchoate form of the hook-like process common in *Pongo* specimens[133]. A distal notch is present laterally, resulting in a discontinuous Mc2 facet and adding to the great ape resemblance of the anterior Mc2 morphology. A distal notch is not in evidence medially, but the slight projection of the distal portion of the hamate facet may have allowed a carpometacarpal ligament to pass, a possibility supported by the specimen's discontinuous Mc4 facet. The body flares laterally to a small degree in the region of the dorsal Mc2 and trapezoid facets, perhaps increasing its embrasure with the trapezoid to some degree. The hamate facet is also discontinuous, with the most distal portion separated from the remainder by a small area of increased rugosity. This allies it somewhat with the brachiators of the extant sample (Fig. S6), although the small size of the intervening region is more comparable with that variably present in *Pongo* and *Nasalis*, and particularly the former, as it lacks the latter's especially deep excavation of the medial surface and distomedial margin for attachment and passage of capitohamate and carpometacarpal ligaments.

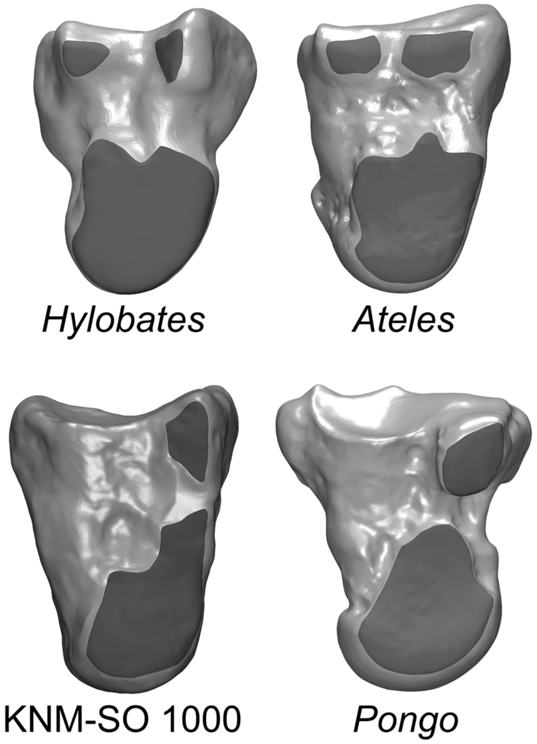

Fig. S6. Visualization of hamate facets (shaded regions) in capitates displaying articular discontinuity. See Table S6b for variation among sampled taxa.

KNM-SO 1001 This right capitate is similar in size to KNM-SO 1000 as measured by volume and surface area, but is shorter and stouter, more similar in its dimensions to the Rusinga specimens and KNM-CA 409. It is well preserved except for small deletions along the distal margins palmarly and dorsomedially, and a larger one dorsolaterally. Despite its small size, this specimen resembles those from Rusinga in many ways. A distal notch is present medially but not laterally, and it has large, continuous facets for both the Mc2 and hamate, both in accord with most of the fossil sample but unlike KNM-SO 1000. The Mc3 surface is also more similar to others of the fossil sample than to KNM-SO 1000. Its mediolateral width remains relatively uniform from dorsal to palmar, and its lateral and palmar borders are less acutely angled than in the other Songhor specimen of similar size. Mc3 topography is less pronounced, with relatively continuous dorsopalmar concavity akin to that of the Rusinga specimens, lacking any hook-like projection palmarly. The facet's distal extent occurs along the Mc2 margin but does not result in notable distal displacement of the palmar Mc2 articulation.

Like KNM-SO 1000 and KNM-CA 409, the head of KNM-SO 1001 lacks pronation, and its distinct lunate facet maintains a directly dorsal orientation across its wide mediolateral extent before forming an angular border with the centrale facet. Unlike others of the fossil sample, the lunate and centrale facets are nearly orthogonal to each other, with the latter having a markedly lateral orientation. The hamate facet of this specimen is also the least concave of the fossil sample, falling just outside the upper range of *Ateles*.

Table S6. Condition of selected articulations in extant sample. Tot, total number of specimens; Cont, facet is continuous dorsopalmarly; Plm, palmar; Dor&plm, distinct facets both dorsally and palmarly; prox, proximal; dist, distal; Plm acc, palmar accessory facet.

	**a**	Mc2			**b** Hamate		
	Tot	Cont	Plm only	Dor& Plm	Prox & dist	Dist Dor&Plm	Plm acc
Pan	44	1	3	40	0	0	2
Gorilla	38	0	25	13	0	0	0
Pongo	34	0	1	33	8	0	0
Hylobatids	29	0	0	29	26	12	1
Papio	14	10	0	4	0	0	0
Lophocebus	6	6	0	0	0	0	0
Mandrillus	9	7	0	2	0	0	0
Cercocebus	2	1	0	1	0	0	0
Macaca	18	18	0	0	0	0	1
Erythrocebus	7	7	0	0	0	0	0
Cercopithecus	11	8	0	3	0	0	0
Colobus	9	1	0	8	0	0	0
Procolobus	13	10	0	3	0	0	0
Nasalis	17	13	0	4	4	3	11
Trachypithecus	17	14	0	3	0	0	0
Presbytis	2	2	0	0	0	0	0
Alouatta	32	28	0	4	0	0	0
Ateles	13	12	0	1	12	1	0
Cebus	28	17	0	11	0	0	0

KNM-SO 31245 This right capitate is larger than those described above, just larger than the KPS III individual of *E. heseloni* and most similar in size to female proboscis monkeys of the sample. It suffers from abrasion along the lunate-centrale and lunate-hamate facet margins palmarly, and along the dorsodistal margin of the hamate facet. More severe damage is present distally, where the dorsal margin of the Mc3 facet has been eroded, progressing in severity toward the lateral extent where the deletion includes the dorsal portion of the Mc2 surface. Despite this damage, the dorsopalmar continuity of the Mc2 surface is evident, with the facet maintaining uniform width at the midline, as opposed to the proximal impingement adjacent to the lateral ligament concavity seen in the other fossil specimens.

This specimen is relatively stout, with the head, neck, and body all mediolaterally expanded slightly. The distal facet is relatively uniform in width, neither narrowing palmarly nor coming to a point palmolaterally, although the prominent ligament attachment site just proximal to the Mc3 facet projects palmarly beyond the MC3 articular surface, as commonly occurs in apes and in each of the fossil specimens save two of the Rusinga specimens. The distal portion of the hamate facet may have a slight palmar orientation, but the abrasion of the margin renders the morphology unclear. The head is pronated, and the centrale facet seems to be somewhat palmarly expanded, though the erosion in this area hinders comparison.

KNM-SO 31246 Also from the right side, this specimen resembles KNM-SO 31245 in many ways while being more pronounced in many of their distinctive shared features. It is also larger, most similar in size to a large proboscis monkey or a small mandrill. While weathering is minor, the specimen suffers from severe erosion along the palmar margin of the lunate facet, growing in severity medially in the region formerly abutting the hamate facet. Damage is most extensive distally, where the Mc3 facet is properly represented in only a small palmomedial region. The unaffected area demonstrates the absence of a distal notch medially, contrary to the condition of KNM-SO 31245. The Mc2 facet has also been almost entirely deleted, although an attachment site for a lateral ligament is preserved. The distal position of this excavation suggests that encroachment on the Mc2 articulation would have been significant, but the presence of a distolateral notch cannot be determined.

Overall mediolateral expansion is more pronounced than in KNM-SO 31245, and the hamate facet's proximodistal concavity is high relative to most of the fossil sample. It also has a weakly expressed dorsal ridge, akin to those sometimes present in great apes. A dorsal portion of the centrale facet is distally expanded, its distal extent positioned palmar to this slightly raised and angular portion of the body (Fig. S7). The distal portion of the hamate facet of KNM-SO 31246 also has a distinct palmar orientation, comparable to the condition of extant apes and lacking the unique morphology of *E. heseloni*. Head pronation is also exaggerated relative to KNM-SO 31245, approaching the degree found in KNM-MV 4 (Fig. S2, proximal view). Its centrale facet appears to have been palmarly expanded in life, but the palmar-most extent of this articulation is now absent. In overall morphology, this specimen bears some resemblance to the less morphologically elaborated *Gorilla* specimens of the sample.

KNM-SO 1002 This right capitate is the largest of the fossil sample, similar in size to the smallest of sampled *Papio* specimens. It suffers from erosion like that of KNM-SO 31246, albeit not as severe, with most of the Mc3 facet and a central portion of the Mc2 facet having been preserved. Palmar portions of the lunate and centrale facets also suffer from erosion, which extends distally and medially to transect the hamate facet, terminating near the dorsodistal border of the lunate surface.

The head of this specimen is large, globular, and greatly expanded laterally, while being somewhat pronated relative to the body. The body is expanded both medially and laterally, and the neck is highly waisted. A dorsal portion of the centrale facet is distally expanded, positioning its distal extent palmar to the laterally-expanded body (Fig. S7). The erosion of the dorsal margin separating the body and head prevents examination of morphology in this region, but the surface just proximal to this area is well preserved and markedly concave, with the lunate surface angling dorsally where it may have contributed to a raised ridge. While a raised ridge in this area is fairly common among catarrhines, the preserved anatomy suggests it may have been quite robust in this specimen, perhaps resembling that of *Nacholapithecus*[134]. The distal portion of the hamate facet maintains a palmar orientation, and is the most concave of the fossil sample, largely owing to the medial expansion of the body. Just palmar to this facet lies a deep excavation for the capitohamate ligament, the most pronounced of the fossil sample. KNM-SO 1002 also shares with many extant apes a distinct palmar indentation just distal to the lunate facet for displacement of the palmar horn of the lunate, which may reflect load-bearing during midcarpal flexion.

The preserved portion of the Mc2 surface is sufficient to demonstrate its dorsopalmar continuity, aligning KNM-SO 1002 with its contemporaries other than KNM-SO 1000 and distinguishing it from the typical extant ape condition (Table S6a). The Mc2 facet is also oriented somewhat distally, a feature most pronounced in Asian apes and *Nasalis* among non-hominin anthropoids. KNM-SO 1002 also lacks a distal notch medially, although the medial projection of the distal portion of the hamate facet leaves a possible passage for a carpometacarpal ligament as in KNM-SO 1000. The preserved portion of the distal facet displays topography like that of contemporaneous catarrhines. It lacks the complex Mc3 facet most pronounced in African apes, *Pongo*, and *Ateles* of the extant sample, and likely the palmar beak often found in great apes as well. The morphology of the head is intermediate between *Pan* and cercopithecines; it appears to lack the palmar extension of the lunate and centrale facets generally seen in *Pan*, but its proximal outline is roughly a quarter sphere, as is commonly true of *Pan*.

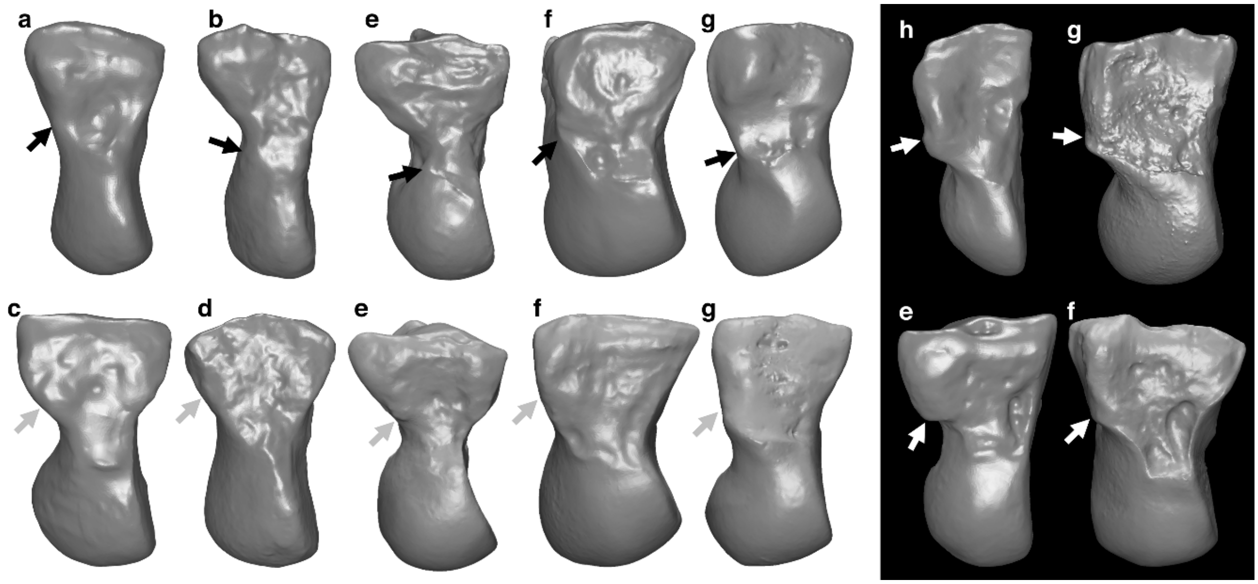

Fig. S7. Variation in scaphoid/centrale facet dorsal margin morphology. Right capitates in dorsal view, with arrows pointing to the facet's dorsodistal extent (black arrows = distal extent visible in dorsal view; light arrows = distal extent occurs palmar to a laterally-projecting portion of the body). Inset: elaborated condition described in the text. (a) KNM-MV 4, (b) KNM-SO 1000, (c) KNM-SO 1002, (d) KNM-SO 31246, (e) *Pongo*, (f) *Gorilla*, (g) *Pan*, (h) *Hoolock*.

Positional classification

Nine shape variables (listed in Table S7b) were found to distinguish between extant positional classes by the criteria described above (Table S4) and were included in the positional classifiers (visualized in Fig. 2). Most suspensors are distinguished from pronograde monkeys along the first axis, with their less curved hamate facets (CpHmC), smaller Mc2 and Mc4 facets (Cp2, Cp4), and more acute proximal angles (CpPxA) wielding the largest influence. Knuckle-walkers overlap with both groups on this axis but are largely distinguished from the other positional classes along the second, on the strength of a less acute proximal angle (CpPxA), a more dorsally positioned head (CpHP), and smaller Mc2 and Mc4 facets (Cp2, Cp4). The cercopithecine hand postures are poorly distinguished by the first two axes, with somewhat better separation occurring along the third axis, led by hamate facet curvature (CpHmC), proximal angle (CpPxA), and size of the dorsal nonarticular region (CpDn). Difficulty in separating digitigrade and palmigrade cercopithecines is the most prominent source of misclassification error for both positional classifiers (morphometrics derived from four elements of the carpus including the capitate are effective in distinguishing palmigrady and digitigrady[34], but the capitate by itself does not sufficiently covary with cercopithecine wrist postures to reliably distinguish these groups). Overall, the cross-validated balanced accuracy of both DFA and *glmnet* classifiers exceeds 90% (Table S7a; see Table S8 for additional details and Table S15a for predictions and posterior probabilities for each extant specimen). Fossil classifications are entirely congruent between the two positional classifiers (Table S7c). Although fossil posterior probabilities are generally high, non-palmigrade fossil specimens are positioned near the decision boundaries, reflecting the subtle, perhaps incipient nature of the positional adaptations of these specimens.

Table S7. Positional classification results

a Extant classification accuracy[a]

Model	Total	*DG*	*KW*	*PG*	*S*	Bal[b]
DFA	0.881	0.633	0.957	0.895	0.870	0.901
glmnet	0.906	0.621	0.976	0.914	0.927	0.919

**b**	Discriminant functions			*glmnet* variable importance[c]			
	DF1	DF2	DF3	*DG*	*KW*	*PG*	*S*
CpSc	0.40	-0.40	-0.35	0.43	1.87	0.21	2.09
CpDn	0.41	-0.19	-0.44	1.44	0.00	0.53	2.01
Cp3	-0.02	-0.31	0.20	1.35	1.78	0.00	0.00
Cp2	0.72	0.58	-0.13	2.50	2.38	0.87	0.99
Cp4	0.76	0.46	0.33	1.67	7.47	2.44	0.00
CpPxA	0.69	-0.93	0.52	0.00	3.24	0.66	4.51
CpScA	0.49	0.36	0.37	0.60	0.40	1.21	1.41
CpHmC	0.76	-0.15	-0.53	2.39	0.58	0.00	3.88
CpHP	0.29	0.49	-0.07	1.77	1.67	1.44	1.54

c Fossil classification results

DFA	*DG*	*KW*	*PG*	*S*	Max	Class
KNM-MV 4	0.04	0.01	0.94	0.00	0.94	*PG*
KNM-CA 409	0.14	0.04	0.80	0.03	0.80	*PG*
KNM-SO 1000	0.00	0.10	0.00	0.90	0.90	*S*
KNM-SO 1001	0.00	0.04	0.01	0.95	0.95	*S*
KNM-SO 31245	0.01	0.01	0.95	0.04	0.95	*PG*
KNM-SO 31246	0.02	0.00	0.96	0.02	0.96	*PG*
KNM-SO 1002	0.00	0.89	0.11	0.00	0.89	*KW*
KPS III(L) C26	0.35	0.00	0.65	0.00	0.65	*PG*
KPS III(R) C28	0.24	0.00	0.76	0.00	0.76	*PG*
KPS VIII C27	0.06	0.00	0.94	0.00	0.94	*PG*
KNM-RU 2036M	0.15	0.00	0.67	0.19	0.67	*PG*
glmnet	*DG*	*KW*	*PG*	*S*	Max	Class
KNM-MV 4	0.00	0.00	1.00	0.00	1.00	*PG*
KNM-CA 409	0.01	0.00	0.99	0.00	0.99	*PG*
KNM-SO 1000	0.00	0.00	0.00	1.00	1.00	*S*
KNM-SO 1001	0.00	0.00	0.00	1.00	1.00	*S*
KNM-SO 31245	0.00	0.00	0.90	0.10	0.90	*PG*
KNM-SO 31246	0.00	0.00	0.96	0.04	0.96	*PG*
KNM-SO 1002	0.00	0.83	0.17	0.00	0.83	*KW*
KPS III(L) C26	0.06	0.00	0.94	0.00	0.94	*PG*
KPS III(R) C28	0.03	0.00	0.97	0.00	0.97	*PG*
KPS VIII C27	0.01	0.00	0.99	0.00	0.99	*PG*
KNM-RU 2036M	0.05	0.00	0.92	0.03	0.92	*PG*

[a] *glmnet* parameters were tuned with 20 repetitions of 10-fold CV; both DFA and *glmnet* model accuracy was calculated after 100 repetitions of 10-fold CV.
[b] Balanced accuracy, an average of sensitivity and specificity (true positive rate and true negative rate).
[c] Absolute value of tuned model coefficients.

Table S8. Additional extant positional classification results

**a**	Cross-validation trials								
	DFA				*glmnet*				
	DG	*KW*	*PG*	*S*	*DG*	*KW*	*PG*	*S*	Total
DG	1900	0	1100	0	1868	0	1132	0	3000
KW	0	7846	354	0	0	7989	46	165	8200
PG	1205	417	13868	10	825	203	14150	322	15500
S	0	500	487	6613	0	189	352	7059	7600
**b**	Additional per-class accuracy metrics								
	DFA				*glmnet*				
Bal[a]	0.788	0.941	0.894	0.981	0.829	0.973	0.915	0.958	
Sen[b]	0.612	0.895	0.877	0.998	0.694	0.953	0.902	0.935	
Spec[c]	0.965	0.986	0.912	0.964	0.964	0.992	0.927	0.980	
PPV[d]	0.633	0.957	0.895	0.870	0.623	0.974	0.913	0.929	
NPV[e]	0.962	0.965	0.897	1.000	0.974	0.985	0.919	0.982	
**c**	Mean prediction posterior probabilities				Mean probabilities by *a priori* class				
DFA	0.756	0.946	0.908	0.969	0.610	0.939	0.865	0.861	
glmnet	0.801	0.959	0.931	0.958	0.589	0.949	0.884	0.915	
**d**	*glmnet* tuned parameters								
alpha	0.9632653								
lambda	0.0005459								

[a] Balanced accuracy – average of sensitivity and specificity
[b] Sensitivity – correct predictions relative to the number of *a priori* cases of that class in the sample. Also known as recall or true positive rate.
[c] Specificity – rate at which observations not assigned to a class actually do not belong to that class, also known as the true negative rate.
[d] Positive prediction value – probability that an observation predicted to belong to a class actually belongs to that class.
[e] Negative prediction value – probability that an observation not predicted to belong to a class actually does not belong to that class.

Superfamily classification

Nine shape variables (listed in Table S9b) met the criteria for inclusion in the taxonomic classifiers (Table S3). In the DFA model (Fig. S8), hominoids are distinguished from ceboids and, to a lesser extent, from cercopithecoids by the first discriminant function. This axis is dominated by the orientation of the scaphoid/central facet (CpScA) and the relative size of the Mc4 facet (Cp4), which tend to be low among hominoids and high among ceboids. The second discriminant function separates most cercopithecoids and ceboids, while also contributing to the hominoid-cercopithecoid distinction. Values along this axis increase most strongly in association with a larger and more distally oriented Mc2 surface (Cp2, Cp23A), and a larger and more proximally oriented proximoradial surface (CpPx, CpPxA). In the *glmnet* model, platyrrhines are best distinguished from other anthropoids by their large Mc4 facets (Cp4) and dorsally-oriented centrale facets (CpScA), and cercopithecoids by their large proximoradial facets (CpPx) and hamate facet concavity (CpHmC). Hominoids are meanwhile best distinguished by small Mc2 and Mc4 facets (Cp2, Cp4) and radially-oriented scaphoid/centrale facets (CpScA; Table S9b).

The DFA and *glmnet* models were both effective in classifying the extant specimens, with balanced accuracy exceeding 95% (Table S9a; see Table S10 for additional details and Table S15b for individual predictions and posterior probabilities). Fossil predictions are congruent between classifiers apart from KNM-MV 4, which the

DFA classifies as a ceboid and *glmnet* classifies as a cercopithecoid with similar confidence (Table S9c). KNM-SO 1000, KNM-SO 1001, and KNM-SO 1002 are classified as hominoids, with the Rusinga sample and remaining Tinderet specimens classified among the cercopithecoids. Posterior probabilities of the *glmnet* predictions are again high, while the DFA model lacks confidence about KNM-SO 1001 and KNM-CA 409, as well as one of the Rusinga specimens. This uncertainty is reflected in the DFA plot, in which the fossil specimens again tend to plot near the decision boundaries as they did in the positional DFA, potentially reflecting the relative temporal proximity of these specimens to their last common ancestor.

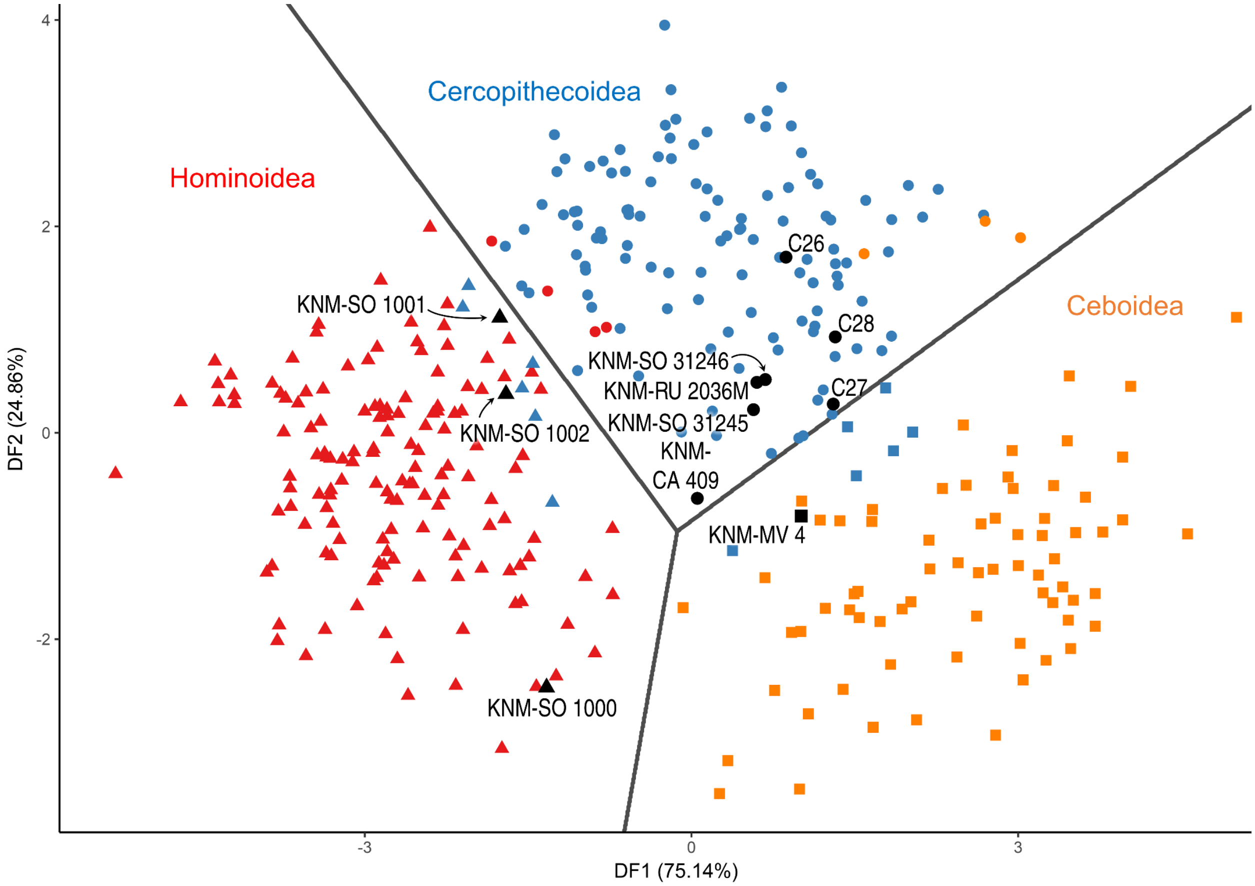

Fig. S8. Discriminant scores based on nine shape variables best distinguishing extant superfamilies. Points are coloured according to *a priori* class and shaped according to predicted class: triangles = Hominoidea, circles = Cercopithecoidea, squares = Ceboidea. Grey lines represent decision boundaries. See Table S9 for discriminant functions, classification accuracy, and posterior probabilities.

Table S9. Taxonomic classification results. Hom, Hominoidea; Cerc, Cercopithecoidea; Plat, Platyrrhini. See Table S7 and text for details.

**a** Extant classification accuracy					
Model	Total	Cerc	Hom	Plat	Bal
DFA	0.943	0.907	0.971	0.947	0.955
glmnet	0.947	0.930	0.961	0.945	0.960

**b**	Discriminant functions			*glmnet* variable importance		
	DF1	DF2		Cerc	Hom	Plat
CpPx	0.08	0.62		1.05	0.00	1.34
Cp2	0.55	0.96		0.00	3.24	0.04
Cp4	0.99	-0.01		0.00	3.17	3.31
Cp23A	-0.41	0.49		0.00	0.16	1.45
CpPxA	-0.09	0.43		0.26	0.00	1.63
CpScA	1.22	-0.37		0.00	3.00	5.10
Cp3SD	-0.19	0.32		0.00	0.02	1.56
CpHmC	0.08	0.14		0.81	0.39	0.00
CpHP	0.31	0.37		0.03	1.69	0.00

**c** Fossil classification results					
DFA	Cerc	Hom	Plat	Max	Class
KNM-MV 4	0.11	0.00	0.89	0.89	Plat
KNM-CA 409	0.52	0.17	0.32	0.52	Cerc
KNM-SO 1000	0.00	0.99	0.01	0.99	Hom
KNM-SO 1001	0.37	0.63	0.00	0.63	Hom
KNM-SO 31245	0.85	0.01	0.14	0.85	Cerc
KNM-SO 31246	0.91	0.01	0.08	0.91	Cerc
KNM-SO 1002	0.14	0.86	0.00	0.86	Hom
KPS III(L) C26	0.99	0.00	0.01	0.99	Cerc
KPS III(R) C28	0.89	0.00	0.11	0.89	Cerc
KPS VIII C27	0.58	0.00	0.42	0.58	Cerc
KNM-RU 2036M	0.92	0.01	0.08	0.92	Cerc
glmnet	Cerc	Hom	Plat	Max	Class
KNM-MV 4	0.90	0.02	0.08	0.90	Cerc
KNM-CA 409	0.80	0.19	0.00	0.80	Cerc
KNM-SO 1000	0.00	1.00	0.00	1.00	Hom
KNM-SO 1001	0.16	0.84	0.00	0.84	Hom
KNM-SO 31245	0.95	0.01	0.04	0.95	Cerc
KNM-SO 31246	0.98	0.00	0.02	0.98	Cerc
KNM-SO 1002	0.14	0.86	0.00	0.86	Hom
KPS III(L) C26	1.00	0.00	0.00	1.00	Cerc
KPS III(R) C28	0.99	0.00	0.01	0.99	Cerc
KPS VIII C27	0.97	0.00	0.03	0.97	Cerc
KNM-RU 2036M	0.99	0.00	0.01	0.99	Cerc

Table S10. Additional extant taxonomic classification results. See Tables S7 and S9 for abbreviations and definitions.

**a**	Cross-validation trials						
	DFA			*glmnet*			
	Cerc	Hom	Plat	Cerc	Hom	Plat	Total
Cerc	11336	632	532	11630	656	214	12500
Hom	417	14083	0	444	13940	116	14500
Plat	300	85	6915	309	92	6899	7300
**b**	Additional per-class accuracy metrics						
Bal	0.944	0.965	0.957	0.950	0.960	0.971	
Sen	0.941	0.952	0.929	0.940	0.949	0.957	
Spec	0.948	0.979	0.986	0.961	0.972	0.985	
PPV	0.907	0.971	0.947	0.931	0.962	0.945	
NPV	0.967	0.964	0.980	0.966	0.962	0.988	
**c**	Mean prediction posterior probabilities			Mean probabilities by *a priori* class			
DFA	0.932	0.966	0.968	0.869	0.953	0.935	
glmnet	0.906	0.938	0.925	0.949	0.960	0.954	
**d**	*glmnet* tuned parameters						
alpha	0.9921053						
lambda	0.0010833						

Hierarchical clustering

All shape variables were found to have significant phylogenetic signal as quantified by both Pagel's λ and Blomberg's K (Table S3b), and no significant allometric correlations were found (Table S2). All variables were therefore included in the hierarchical clustering analysis (apart from the collinear CpPx). The BioNJ algorithm effectively represents the underlying distance matrix, with an extant cophenetic correlation coefficient of 0.946. Relationships between the extant taxa and fossil specimens are represented in two separate dendrograms preserving the relationships found in individual analyses (Fig. S9a,b).

Capitate morphology again effectively distinguishes the anthropoid superfamilies, but relationships within these groups often vary from those determined from molecular data (Fig. S9c). *E. heseloni*, KNM-CA 409, and KNM-MV 4 are positioned as basal catarrhines, while KNM-SO 31245 and KNM-SO 31246 plot together as sister to a group comprising the hominoids, cercopithecines, and two of the three presbytins. KNM-SO 1000 and KNM-SO 1001 are grouped within an Asian ape clade, with the former more similar to hylobatids and the latter more similar to the *Pongo* species. KNM-SO 1002 is positioned as a basal member of the African ape clade.

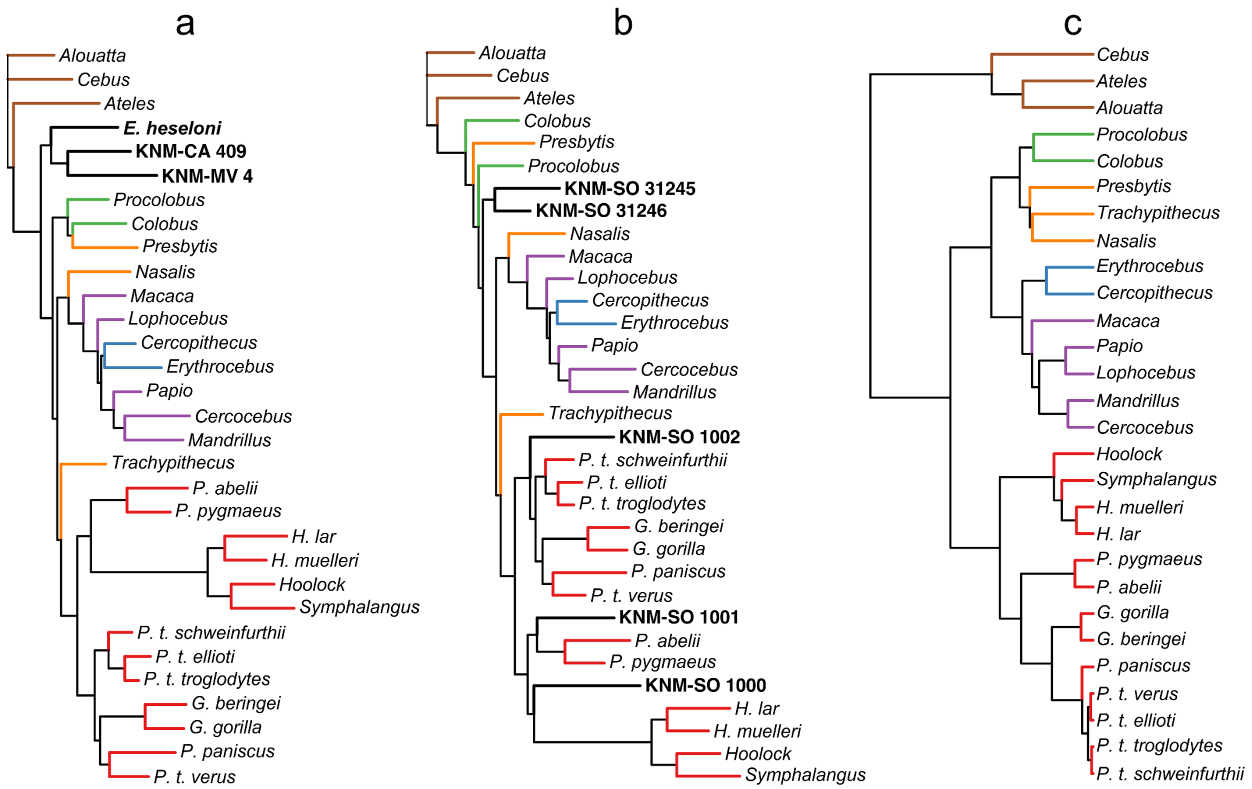

Fig. S9. Phenetic and phylogenetic relationships among extant and fossil specimens. (a and b) BioNJ dendrograms. Fossil specimens are split into two trees to best preserve their relationships to extant taxa when analysed individually, (c) molecular phylogeny utilized in phylogenetic comparative analyses. Branch tips coloured according to major anthropoid taxonomic divisions.

Locomotor proportion estimation

Predictive models for suspensory and quadrupedal proportions are detailed in Table S11. Accuracy was high in the suspensory models, while those for quadrupedalism struggled to distinguish terrestrial taxa from arboreal cercopithecines and colobus monkeys. The six extant taxa for which published observations were unavailable are assigned values generally in accord with expectation, other than *Cercocebus* and *Mandrillus* being assigned similar values of *Quad*.

Fossil estimates (Table S12) are largely consistent with the classification results, but the different locomotor estimates for each specimen do not necessarily tell a consistent story. KNM-SO 1000 and KNM-SO 1001 are assigned the highest values of *SuspA*, with the latter estimated to be the fossil individual most reliant on suspension during arboreal locomotion, with a frequency exceeding that of extant *Ateles*. This is reversed in the *Susp* estimates, of which KNM-SO 1000 is assigned the highest value, in accord with its greater qualitative resemblance to extant suspensors, while KNM-SO 1001 is estimated as only slightly more suspensory than most of its Miocene contemporaries. *QuadA* is estimated to be very low in KNM-SO 1001, but only slightly reduced in KNM-SO 1000 relative to most others of the Miocene sample. This trend is reversed in *Quad*, with KNM-SO 1000 assigned the lowest value of the fossil sample and KNM-SO 1001 representing the sample median. KNM-SO 1002 is predicted as the least suspensory of the fossil sample, with estimates corresponding to the baseline values assigned to non-suspensors of the extant sample (Table S11b). Its *Quad*

estimate is the highest of the fossil sample and exceeds the estimated (but not observed) values of most extant taxa as well, including most of the African apes. Its *QuadA* estimate, meanwhile, is similar to those assigned to many other extant and fossil specimens, which would be consistent with a substantial terrestrial component.

Locomotor estimates for the fossil specimens classified as palmigrade are fairly consistent with each other. The Rusinga specimens are all predicted very similarly save for a low *Quad* estimate for KNM-RU 2036, while KNM SO 31245 and KNM-SO 31246 are very similar to each other and, to a somewhat lesser degree, to the Rusinga specimens. KNM-SO 409 has a slightly elevated *QuadA* estimate and a substantially lower *Arb* estimate, while KNM-MV 4 has slightly lower estimates for *QuadA* and *Susp*. These specimens are otherwise estimated to have locomotion similar to that of the Rusinga specimens.

Table S11. Locomotor proportion models and extant estimates

a Predictive models

	PGLS			GLM					
	R^2	λ	p	Terms	Coef	SE	T	p	%SEE[a]
QuadA	0.901	0.000	**0.000**	(Intercept)	-0.55	0.04	-12.8	0.000	15.4
				Cp23A	-0.09	0.05	-1.9	0.061	
				Cp3SD	0.27	0.05	6.0	0.000	
				CpDn	0.26	0.05	5.6	0.000	
				CpHP	0.22	0.05	4.9	0.000	
				CpScA	0.70	0.05	13.0	0.000	
Quad	0.638	0.421	**0.000**	(Intercept)	0.15	0.06	2.3	0.020	20.5
				CpDn	0.36	0.07	5.3	0.000	
				CpPx	0.70	0.07	9.7	0.000	
				CpPxA	0.79	0.08	10.3	0.000	
SuspA	0.969	0.000	**0.000**	(Intercept)	-2.55	0.07	-34.6	0.000	8.5
				CpDn	-0.36	0.06	-6.3	0.000	
				CpHmC	-0.23	0.07	-3.24	0.001	
				CpPxA	-0.62	0.09	-6.8	0.000	
				CpSc	-0.48	0.06	-7.6	0.000	
				CpScA	-0.54	0.08	-6.8	0.000	
Susp	0.945	0.000	**0.000**	(Intercept)	-3.10	0.12	-26.2	0.000	9.6
				CpPxA	-1.34	0.08	-17.8	0.000	
				CpSc	-0.79	0.09	-9.0	0.000	

b Predicted locomotor proportions of training taxa[b].

	QuadA			*Quad*			*SuspA*			*Susp*		
	Obs	Pred	Δ	Obs	Pred	Δ	Obs	Pred	Δ	Obs	Pred	Δ
P. t. schwein.	0.31	0.30	0.01	0.93	0.61	0.32	0.08	0.06	0.02	0.01	0.03	0.02
P. t. verus	0.21	0.26	0.05	0.86	0.69	0.17	0.06	0.06	0.00	0.01	0.02	0.01
P. paniscus	0.35	0.28	0.07	0.87	0.70	0.17	0.09	0.06	0.03	0.01	0.03	0.02
G. gorilla	0.19	0.33	0.14	0.92	0.76	0.16	0.13	0.06	0.07	0.01	0.03	0.02
G. beringei	0.53	0.41	0.12	0.96	0.73	0.23	0.06	0.07	0.01	0.01	0.05	0.04
P. pygmaeus	0.12	0.20	0.08	0.12	0.33	0.21	0.43	0.34	0.09	0.43	0.25	0.18
P. abelii	0.18	0.23	0.05	0.18	0.33	0.15	0.38	0.37	0.01	0.38	0.36	0.02
Hoolock	0.00	0.17	0.17	0.00	0.10	0.10	0.55	0.44	0.11	0.55	0.51	0.04
H. lar	0.00	0.15	0.15	0.00	0.07	0.07	0.59	0.61	0.02	0.59	0.58	0.01
Symphalangus	0.00	0.19	0.19	0.00	0.12	0.12	0.59	0.56	0.03	0.59	0.59	0.00
Papio	0.68	0.45	0.23	0.99	0.77	0.22	0.00	0.03	0.03	0.00	0.02	0.02
Lophocebus	0.42	0.51	0.09	0.42	0.79	0.37	0.00	0.02	0.02	0.00	0.01	0.01
Macaca	0.68	0.41	0.27	0.68	0.74	0.06	0.00	0.04	0.04	0.00	0.02	0.02

Erythrocebus	0.60	0.57	0.03	0.94	0.68	0.26	0.00	0.04	0.04	0.00	0.04	0.04
Cercopithecus	0.54	0.49	0.05	0.54	0.73	0.19	0.00	0.04	0.04	0.00	0.02	0.02
Colobus	0.41	0.48	0.07	0.41	0.71	0.30	0.01	0.02	0.01	0.01	0.01	0.00
Procolobus	0.35	0.37	0.02	0.35	0.62	0.27	0.01	0.05	0.04	0.01	0.03	0.02
Trachypithecus	0.60	0.51	0.09	0.60	0.65	0.05	0.00	0.06	0.06	0.00	0.03	0.03
Presbytis	0.28	0.39	0.11	0.28	0.49	0.21	0.02	0.05	0.03	0.02	0.03	0.01
Alouatta	0.61	0.52	0.09	0.61	0.49	0.12	0.02	0.04	0.02	0.02	0.03	0.01
Ateles	0.42	0.46	0.04	0.42	0.28	0.14	0.25	0.13	0.12	0.25	0.17	0.08
Cebus	0.37	0.45	0.08	0.37	0.44	0.07	0.00	0.06	0.06	0.00	0.05	0.05

c Predicted locomotor proportions for other taxa

	QuadA	*Quad*	*SuspA*	*Susp*
P. t. troglodytes	0.30	0.62	0.06	0.02
P. t. ellioti	0.29	0.63	0.07	0.03
H. muelleri	0.19	0.08	0.55	0.44
Mandrillus	0.39	0.75	0.04	0.03
Cercocebus	0.58	0.77	0.03	0.02
Nasalis	0.41	0.65	0.05	0.02

[a] Percent standard error of the estimate based on repeated individual predictions generated during cross validation.
[b] Predictions calculated after 100 repetitions of 10-fold cross validation of quasibinomial logistic regression. Obs, observed proportions. Pred, predicted proportions. Δ, residual.

Table S12. Estimated locomotor proportions for fossil specimens

Specimen	*QuadA*	*Quad*	*SuspA*	*Susp*
KNM-MV 4	0.35	0.57	0.07	0.03
KNM-CA 409	0.61	0.54	0.06	0.08
KNM-SO 1000	0.37	0.24	0.19	0.19
KNM-SO 1001	0.16	0.50	0.32	0.10
KNM-SO 31245	0.40	0.33	0.11	0.06
KNM-SO 31246	0.41	0.32	0.09	0.06
KNM-SO 1002	0.42	0.76	0.04	0.01
KPS III(L) C26	0.51	0.51	0.10	0.08
KPS III(R) C28	0.46	0.51	0.09	0.09
KPS VIII C27	0.48	0.52	0.08	0.05
KNM-RU 2036M	0.52	0.27	0.11	0.10
E. heseloni mean	0.49	0.45	0.09	0.08

Analysis of locomotor diversity

The PLS shape scores of all extant centroids and fossil specimens are visualized in Fig. 3a. The *E. heseloni* specimens group together among the cercopithecoids, flanked by the ceboids and colobins. KNM-SO 31245 and 31246 are nearby, positioned near *Procolobus* between the Rusinga specimens and papionins. KNM-SO 1000 and 1002 plot among the great apes, while KNM-CA 409, KNM-MV 4, and KNM-SO 1001 are positioned between the great ape and cercopithecoid groups, with the latter separated somewhat from the other specimens in the general direction of the hylobatids. The Euclidean area of the Tinderet sample's convex hull exceeds that of the Old World monkey centroids, although the great ape sample occludes the greatest portion of shape-space (Table S13c).

Much of the separation between groups occurs along the second PLS axis, but the structure of the shape space is dominated by the extreme shape and locomotor variables of the hylobatids, resulting in scores along the first axis increasing with quadrupedalism and decreasing with suspension, with relatively little morphological covariation with climbing or leaping. With hylobatids excluded, morphological covariation with suspension still wields the strongest influence, but parity with other behaviours is greatly increased (Table S13a). This results in recognition of far greater functional diversity among the cercopithecoids; the Euclidean area of the convex hull formed by their centroids in the new PLS shape-space (Fig. 3b) exceeds that of the great apes (Table S13c). Diversity estimated for the Tinderet sample is larger still, with its convex hulls comprising 17.2% of the shape-space. The groups are largely distinguished along the first axis, with morphology associated with quadrupedalism toward the right and with climbing and suspension toward the left. Within the broad groupings, most of the variation occurs along the second axis, which is most strongly influenced by morphological covariates of suspension and arboreality. Morphology associated with greater proportions of *SuspA* and *Arb* is positioned lower on this axis, while more terrestrial taxa and those more reliant on quadrupedalism and climbing during arboreal locomotion are positioned toward the top.

Table S13. PLS results

**a** Locomotor PLS vectors				Without hylobatids		
	PLS1	PLS2	PLS3	PLS1	PLS2	PLS3
QuadA	0.54	0.28	-0.50	0.47	0.38	-0.55
SuspA	-0.71	-0.22	-0.24	-0.54	-0.51	-0.39
ClimbA	0.29	-0.56	0.60	-0.43	0.31	0.46
LeapA	0.03	0.58	0.51	0.45	-0.28	0.58
Arb	-0.36	0.47	0.26	0.31	-0.64	-0.04
**b** Shape PLS vectors						
CpSc	0.31	0.27	-0.08	0.34	0.27	0.13
CpLu	0.21	-0.32	-0.11	-0.37	0.05	-0.07
CpDn	-0.04	-0.11	-0.60	0.10	0.51	-0.48
Cp3	0.26	-0.10	0.14	-0.12	0.07	0.17
CpHm	-0.04	0.31	0.05	0.22	-0.17	0.00
Cp2	0.14	0.52	-0.16	0.44	-0.07	-0.03
Cp4	-0.15	0.40	-0.18	0.36	-0.13	-0.24
Cp23A	-0.37	0.00	0.07	-0.06	-0.30	-0.07
Cp3HmA	0.27	-0.05	0.04	-0.14	-0.09	0.09
CpPxA	0.44	0.09	0.13	0.24	0.40	0.52
CpScA	0.30	0.38	0.09	0.39	-0.14	0.18
Cp3SD	0.25	-0.27	-0.49	-0.18	0.32	-0.31
CpHmC	0.41	-0.10	-0.09	-0.04	0.47	0.17
CpHP	-0.14	0.21	-0.52	0.29	0.02	-0.47
**c** Convex hull Euclidean area[a]						
	OWM	Great apes	Tinderet	OWM	Great apes	Tinderet
	5.58	8.93	6.41	15.43	12.96	17.18

[a] Proportion of scaled PLS shape-space

The relative positions among the monkeys indicate that this analysis better characterizes extant locomotor repertoires with hylobatids excluded. In the full-sample analysis, they tend to plot near their closest relatives regardless of locomotor behaviour apart from the presbytins (orange points). With hylobatids excluded, *Ateles* plots far from the other ceboids and there is better differentiation between the terrestrial and arboreal papionins, although *Erythrocebus* does not group with the other terrestrial cercopithecines, reflecting the lack of terrestrial signal in the capitate relative to other carpals[35]. The projected positions of the extant taxa for which quantitative locomotor observations are unavailable accord largely with expectations. The hominoids plot near their closest relatives, and within the papionin cluster *Mandrillus* is nearest *Papio*, and *Cercocebus* is nearest *Lophocebus*. *Nasalis* is difficult to interpret due to how little is known of its locomotor behaviour. In the full-sample analysis it is pulled intriguingly toward the hylobatids, which, along with its occasional hamate facet discontinuity (Table S6), comport with previous observations suggesting a yet unwitnessed brachiation habit[135,136]. Its position with hylobatids excluded is somewhat less suggestive, but it remains adjacent to occasional brachiatiors.

As in the first PLS analysis, *E. heseloni* clusters among the cercopithecoids, with KNM-SO 31245 and 31246 again most adjacent of the Tinderet sample. KNM-SO 1000 is positioned among the great apes as before, while KNM-SO 1002 plots just outside the great ape hull nearest the *Pan* centroids. KNM-CA 409 and KNM-MV 4 plot nearest the papionins this time, while KNM-SO 1001 is again separated from the other fossils in accord with its resemblance to extant suspensors.

As noted in the text, comparison of PLS scores of individual fossils with PLS centroids of extant taxa is potentially problematic. A given fossil may not accurately represent its population, and the Tinderet locomotor diversity estimate will therefore be more sensitive to outlying morphology or measurement error, potentially inflating the diversity estimate of the fossil sample. On the other hand, the larger number of extant centroids (12 cercopithecoid and 9 great ape taxa vs. 7 Tinderet specimens) potentially biases the diversity estimates toward the extant groups.

Phylogenetic tree (Newick format)

(((((Cercopithecus:9.848126,Erythrocebus:9.848126):5.027053,(((Cercocebus:5.304298,Mandrillus:5.304297):6.046166,(Lophocebus:5.769163,Papio:5.769163):5.5813):1.502062,Macaca:12.852525):2.022653):6.535196,((Colobus:12.537452,Procolobus:12.537451):2.897705,((Nasalis:12.784647,Trachypithecus:12.784648):0.523455,Presbytis:13.308102):2.127056):5.975217):8.589626,((Hoolock:8.241196,((H._muelleri:3.528966,H._lar:3.528966):3.069395,Symphalangus:6.598362):1.642834):11.36475,(((G._beringei:2.558516,G._gorilla:2.558516):6.093717,(P._paniscus:2.333553,((P._t._schweinfurthii:0.344859,P._t._troglodytes:0.344859):0.736563,(P._t._ellioti:0.505401,P._t._verus:0.505401):0.576022):1.252131):6.318679):6.480222,(P._abelii:3.825854,P._pygmaeus:3.825854):11.306601):4.473491):10.394055):16.811821,((Alouatta:14.76024,Ateles:14.76024):6.56106,Cebus:21.321301):25.490521);

Table S14. Tinderet specimen morphometric values, with means (standard deviations) for *E. heseloni* and extant genera. See Table 1 and Fig. S4 for metric details.

	Relative surface areas (%)								Articular angles (°)				Other metrics		
	CpPx	CpSc	CpLu	CpDn	Cp3	CpHm	Cp2	Cp4	Cp23A	Cp3HmA	CpPxA	CpScA	Cp3SD	CpHmC	CpHP
KNM-MV 4	23.6	14.6	9.0	15.9	13.1	8.7	5.3	2.1	72.1	89.9	58.4	102.0	4.6	13.0	0.79
KNM-CA 409	23.3	13.5	9.8	17.6	12.0	10.9	4.7	0.7	81.5	87.0	52.3	108.9	5.7	12.6	0.91
KNM-SO 1000	20.9	12.2	8.8	15.6	12.1	9.2	3.9	0.7	76.6	93.8	47.5	101.5	5.8	10.6	0.75
KNM-SO 1001	26.1	12.7	13.3	13.7	12.7	11.1	5.6	0.9	88.6	82.1	53.0	88.1	5.0	7.4	0.85
KNM-SO 31245	22.1	13.0	9.1	13.6	13.6*	10.9	6.2	0.9	91.5	82.3	56.4*	111.8	5.1	10.8	0.82
KNM-SO 31246	24.4	15.5	8.9	12.8	13.2	10.1	5.7*	1.1	99.5*	87.3	50.3	114.9	5.3	15.2	0.83*
KNM-SO 1002	24.2	13.3	11.0	15.8	12.7	7.4	4.1*	0.4	105.6	87.8	69.6	108.5	5.3*	15.7	0.82
E. heseloni	25.3 (1.2)	15.2 (0.8)	10.1 (1.3)	14.9 (0.6)	12.3 (0.6)	12.3 (0.7)	5.8 (0.3)	1.1 (0.3)	86.5 (3.6)	89.4 (1)	48.5 (5.4)	107.4 (2.9)	5 (0.8)	14.1 (0.3)	1.01 (0.04)
Pan	25.4 (2.7)	15.4 (1.4)	10 (2.2)	15.2 (1.2)	13.1 (1)	9.8 (1.1)	3.9 (1)	0 (0.1)	100.1 (7.5)	88.4 (7.2)	60 (5.9)	102.5 (5)	5.2 (0.8)	16.4 (3.6)	0.72 (0.08)
Gorilla	28.4 (2.2)	15.2 (1.9)	13.3 (1.4)	14.6 (1.8)	14.4 (1.1)	9.7 (1)	2.5 (0.9)	0.2 (0.3)	96.7 (7.6)	84.5 (4.9)	59.3 (7.6)	102.6 (5.1)	6.1 (1.1)	21.2 (3.9)	0.8 (0.09)
Pongo	25.2 (2.4)	12.5 (1.7)	12.7 (2)	14.3 (1.4)	12 (1.4)	9.7 (1.3)	3.6 (0.6)	0.4 (0.4)	106.8 (7.5)	90.2 (7.4)	42 (8.9)	92.5 (6.2)	5.5 (0.9)	13.9 (4.9)	0.85 (0.1)
Hoolock	19.7 (2.1)	14.6 (2)	5 (1.3)	17.5 (1.8)	9.8 (0.9)	10.3 (1.3)	3.8 (0.3)	2.3 (0.6)	113.5 (7.5)	73.9 (1.1)	27.3 (5.1)	83.6 (3.9)	3.4 (0.7)	3.2 (2.9)	0.94 (0.08)
Hylobates	20.4 (1.8)	13.9 (1.5)	6.5 (1.5)	15.2 (1.6)	11.2 (0.8)	10.9 (1.3)	3.9 (0.8)	2.7 (0.7)	117.9 (8)	66 (4.1)	27.6 (5)	87.5 (5.7)	4.3 (0.9)	-4.7 (2)	0.89 (0.08)
Symphalangus	20.6 (0.5)	13.5 (2.1)	7.1 (1.6)	17.6 (0.6)	10.3 (1)	10.3 (1.9)	4.3 (0.7)	1.4 (0.3)	120.3 (1.3)	73.1 (5.3)	27.5 (8.2)	80.7 (1)	3.7 (0.3)	-3.3 (1.7)	1.1 (0.1)
Papio	28.3 (1.4)	17.9 (2.1)	10.3 (1.8)	17.1 (1.6)	11 (0.7)	10.4 (0.7)	5.4 (0.5)	1.3 (0.5)	100.1 (4.4)	88.3 (4.5)	53.6 (7.2)	102.1 (6.1)	5.5 (0.6)	20.4 (3.2)	0.92 (0.08)
Lophocebus	27.7 (2.7)	17.8 (2)	9.9 (1.8)	16.5 (1.1)	12 (0.7)	10.5 (0.8)	5 (0.6)	1.4 (0.7)	96.1 (6.6)	87.1 (5.9)	60.3 (5)	107.5 (1.9)	5.1 (0.6)	20 (1.6)	0.94 (0.15)
Mandrillus	28 (2.6)	18.4 (2.6)	9.7 (0.8)	18 (2.3)	10.6 (1.1)	9.6 (0.8)	5.6 (0.6)	0.7 (0.2)	93.9 (4.4)	101.2 (5)	50 (6.8)	96.2 (4.1)	4.7 (0.7)	18.2 (2.5)	0.95 (0.06)
Cercocebus	27.9 (0.5)	18.8 (2.1)	9 (1.7)	18.5 (0.2)	10.9 (0.2)	10 (0.9)	5.2 (0)	1.1 (0.2)	105.2 (3.4)	88.6 (0.2)	49.1 (3.3)	100 (6.9)	6.3 (0.1)	16.3 (2.8)	1.07 (0.01)
Macaca	27.4 (2.3)	18 (2)	9.4 (1.7)	15.7 (2)	12.3 (0.8)	9.9 (0.8)	4.9 (0.6)	1.4 (0.6)	107.6 (11.9)	83.1 (7.3)	57.8 (9.4)	103.9 (6)	5.9 (0.8)	18.3 (2.5)	0.88 (0.07)
Erythrocebus	26.3 (1.5)	17.2 (2.9)	9.1 (3)	16.2 (1)	12 (0.6)	10.8 (0.9)	6.3 (0.7)	1.7 (0.5)	86 (4)	87.5 (3.1)	55.3 (4.6)	105.4 (8.3)	5.8 (0.7)	22.1 (1.8)	1.06 (0.05)
Cercopithecus	28.1 (1.7)	18.7 (2.2)	9.4 (2.1)	15.9 (0.9)	11.9 (0.9)	10.8 (0.9)	5.8 (0.4)	1.5 (0.4)	94.9 (7.6)	92.2 (3.7)	53.8 (7)	105.7 (6.3)	5.7 (0.9)	19.7 (2.3)	0.94 (0.04)
Colobus	27 (0.8)	18.4 (1.2)	8.6 (1.5)	14 (1.5)	12.5 (0.7)	10.1 (1)	4.6 (0.4)	1.4 (0.7)	99.1 (7.2)	85.6 (6)	62.1 (7.1)	120.8 (4.3)	3.8 (0.6)	13 (2)	0.85 (0.05)
Procolobus	25.5 (1.3)	15.5 (1.7)	10 (1.3)	14.6 (0.9)	12.7 (0.8)	9.9 (1)	5.7 (1)	1.2 (0.6)	102 (7.6)	92.6 (5.2)	60.1 (8.1)	112.6 (7.3)	3.8 (1)	17.1 (3.8)	0.82 (0.09)
Nasalis	26.1 (1.2)	15.7 (1)	10.4 (0.8)	14.6 (0.9)	10.9 (0.9)	10.3 (0.7)	5.7 (0.7)	1.4 (0.5)	115.1 (4.1)	82.6 (3.6)	59.6 (3.3)	108.4 (5)	5 (0.6)	14.5 (2.9)	0.95 (0.05)
Trachypithecus	25 (2)	14.4 (1.7)	10.6 (2)	15.3 (1.2)	13.2 (0.8)	10.2 (0.7)	5.2 (0.5)	1.2 (0.4)	100.9 (6.3)	83 (3.9)	61.2 (6.2)	112.4 (9.5)	5.7 (0.8)	12.9 (2.3)	0.86 (0.07)
Presbytis	25.9 (4.9)	17.2 (2.8)	8.7 (2)	12.8 (2.6)	12.4 (0.5)	11.7 (2)	6.1 (0.1)	0.9 (0.2)	106.6 (11.5)	92.5 (4)	55.4 (5.2)	118 (1.8)	4.2 (0.2)	14.6 (0.1)	0.84 (0.09)
Alouatta	23.5 (1.6)	15.9 (1.8)	7.6 (1.4)	15.2 (1.6)	13.8 (1.8)	11.4 (1)	5.1 (1)	2.2 (0.8)	96.5 (8.2)	84.7 (3.3)	56.6 (5.4)	122 (6.3)	3.1 (0.7)	10.5 (2.4)	0.9 (0.07)
Ateles	24.8 (2.2)	16 (3.3)	8.8 (2)	14 (1.7)	14.3 (0.7)	12 (1)	4.2 (1.1)	2 (0.6)	93.9 (9.8)	92.5 (4.1)	41.4 (6)	121 (8.4)	4 (0.8)	-0.5 (5.2)	0.77 (0.07)
Cebus	23.4 (1.2)	15.8 (1.2)	7.6 (1.2)	15.1 (1)	13.4 (1.1)	11.6 (1)	4.5 (0.8)	3.4 (0.7)	84.8 (5)	76.8 (3.6)	53.7 (4.8)	113.7 (7)	4.3 (0.6)	10.4 (2)	0.82 (0.07)

* imputed values

Table S15. Extant classification with average posterior probabilities calculated after 100 cross-validation repeats. Misclassifications in bold.

		**a** Positional classification											**b** Taxonomic classification								
			DFA					*glmnet*						DFA				*glmnet*			
Specimen	Taxon	Class	*DG*	*KW*	*PG*	*S*	Pred	*DG*	*KW*	*PG*	*S*	Pred	Class	Cerc	Hom	Plat	Pred	Cerc	Hom	Plat	Pred
AMNH 51202	*P. t. schwein.*	*KW*	0.00	1.00	0.00	0.00	*KW*	0.00	1.00	0.00	0.00	*KW*	Hom	0.00	1.00	0.00	Hom	0.00	1.00	0.00	Hom
AMNH 51205	*P. t. schwein.*	*KW*	0.03	0.19	0.78	0.00	***PG***	0.01	0.69	0.30	0.00	*KW*	Hom	0.10	0.90	0.00	Hom	0.29	0.71	0.00	Hom
AMNH 51278	*P. t. schwein.*	*KW*	0.00	0.99	0.00	0.01	*KW*	0.00	0.97	0.00	0.02	*KW*	Hom	0.00	1.00	0.00	Hom	0.00	1.00	0.00	Hom
AMNH 51376	*P. t. schwein.*	*KW*	0.00	0.99	0.01	0.01	*KW*	0.00	1.00	0.00	0.00	*KW*	Hom	0.10	0.90	0.00	Hom	0.05	0.95	0.00	Hom
AMNH 51377	*P. t. schwein.*	*KW*	0.00	0.99	0.01	0.00	*KW*	0.00	1.00	0.00	0.00	*KW*	Hom	0.00	1.00	0.00	Hom	0.01	0.99	0.00	Hom
AMNH 51379	*P. t. schwein.*	*KW*	0.00	0.97	0.03	0.00	*KW*	0.00	1.00	0.00	0.00	*KW*	Hom	0.03	0.97	0.00	Hom	0.02	0.98	0.00	Hom
AMNH 51381	*P. t. schwein.*	*KW*	0.00	0.98	0.00	0.02	*KW*	0.00	0.79	0.00	0.21	*KW*	Hom	0.00	1.00	0.00	Hom	0.00	1.00	0.00	Hom
AMNH 51393	*P. t. schwein.*	*KW*	0.00	1.00	0.00	0.00	*KW*	0.00	1.00	0.00	0.00	*KW*	Hom	0.00	1.00	0.00	Hom	0.00	1.00	0.00	Hom
AMNH 201588	*P. t. schwein.*	*KW*	0.00	1.00	0.00	0.00	*KW*	0.00	1.00	0.00	0.00	*KW*	Hom	0.00	1.00	0.00	Hom	0.00	1.00	0.00	Hom
NMNH 236971	*P. t. schwein.*	*KW*	0.00	1.00	0.00	0.00	*KW*	0.00	1.00	0.00	0.00	*KW*	Hom	0.00	1.00	0.00	Hom	0.00	1.00	0.00	Hom
AMNH 54330	*P. t. trog.*	*KW*	0.00	1.00	0.00	0.00	*KW*	0.00	1.00	0.00	0.00	*KW*	Hom	0.00	1.00	0.00	Hom	0.00	1.00	0.00	Hom
AMNH 90189	*P. t. trog.*	*KW*	0.00	0.98	0.02	0.00	*KW*	0.00	0.74	0.25	0.01	*KW*	Hom	0.00	1.00	0.00	Hom	0.00	1.00	0.00	Hom
AMNH 90190	*P. t. trog.*	*KW*	0.04	0.47	0.49	0.00	***PG***	0.00	0.96	0.03	0.00	*KW*	Hom	0.17	0.83	0.00	Hom	0.16	0.84	0.00	Hom
AMNH 90191	*P. t. trog.*	*KW*	0.00	1.00	0.00	0.00	*KW*	0.00	1.00	0.00	0.00	*KW*	Hom	0.00	1.00	0.00	Hom	0.00	1.00	0.00	Hom
AMNH 90292	*P. t. trog.*	*KW*	0.00	0.95	0.04	0.00	*KW*	0.00	0.98	0.02	0.00	*KW*	Hom	0.01	0.99	0.00	Hom	0.01	0.99	0.00	Hom
AMNH 167342	*P. t. trog.*	*KW*	0.00	0.97	0.03	0.00	*KW*	0.00	1.00	0.00	0.00	*KW*	Hom	0.04	0.96	0.00	Hom	0.03	0.97	0.00	Hom
AMNH 167343	*P. t. trog.*	*KW*	0.00	1.00	0.00	0.00	*KW*	0.00	1.00	0.00	0.00	*KW*	Hom	0.00	1.00	0.00	Hom	0.00	1.00	0.00	Hom
AMNH 167344	*P. t. trog.*	*KW*	0.06	0.14	0.80	0.00	***PG***	0.05	0.57	0.38	0.00	*KW*	Hom	0.69	0.31	0.00	**Cerc**	0.86	0.14	0.00	**Cerc**
AMNH 167346	*P. t. trog.*	*KW*	0.00	0.99	0.00	0.01	*KW*	0.00	1.00	0.00	0.00	*KW*	Hom	0.00	1.00	0.00	Hom	0.00	1.00	0.00	Hom
AMNH 201469	*P. t. trog.*	*KW*	0.00	0.83	0.16	0.01	*KW*	0.00	0.90	0.07	0.03	*KW*	Hom	0.18	0.82	0.00	Hom	0.11	0.89	0.00	Hom
UMMZ 39507	*P. t. trog.*	*KW*	0.00	1.00	0.00	0.00	*KW*	0.00	1.00	0.00	0.00	*KW*	Hom	0.00	1.00	0.00	Hom	0.00	1.00	0.00	Hom
MCZ 15312	*P. t. trog.*	*KW*	0.00	0.98	0.02	0.00	*KW*	0.00	1.00	0.00	0.00	*KW*	Hom	0.01	0.99	0.00	Hom	0.01	0.99	0.00	Hom
AMNH 89351	*P. t. verus*	*KW*	0.00	1.00	0.00	0.00	*KW*	0.00	1.00	0.00	0.00	*KW*	Hom	0.00	1.00	0.00	Hom	0.00	1.00	0.00	Hom
AMNH 89353	*P. t. verus*	*KW*	0.00	1.00	0.00	0.00	*KW*	0.00	1.00	0.00	0.00	*KW*	Hom	0.00	1.00	0.00	Hom	0.00	1.00	0.00	Hom
AMNH 89354	*P. t. verus*	*KW*	0.00	1.00	0.00	0.00	*KW*	0.00	1.00	0.00	0.00	*KW*	Hom	0.00	1.00	0.00	Hom	0.00	1.00	0.00	Hom
AMNH 89355	*P. t. verus*	*KW*	0.00	1.00	0.00	0.00	*KW*	0.00	1.00	0.00	0.00	*KW*	Hom	0.00	1.00	0.00	Hom	0.00	1.00	0.00	Hom
AMNH 89406	*P. t. verus*	*KW*	0.00	1.00	0.00	0.00	*KW*	0.00	1.00	0.00	0.00	*KW*	Hom	0.00	1.00	0.00	Hom	0.00	1.00	0.00	Hom
AMNH 174860	*P. t. verus*	*KW*	0.00	1.00	0.00	0.00	*KW*	0.00	1.00	0.00	0.00	*KW*	Hom	0.02	0.98	0.00	Hom	0.01	0.99	0.00	Hom
AMNH 174861	*P. t. verus*	*KW*	0.01	0.37	0.62	0.00	***PG***	0.00	1.00	0.00	0.00	*KW*	Hom	0.86	0.14	0.00	**Cerc**	0.88	0.12	0.00	**Cerc**
NMNH 256973	*P. t. verus*	*KW*	0.00	0.90	0.10	0.00	*KW*	0.00	0.99	0.01	0.00	*KW*	Hom	0.39	0.61	0.00	Hom	0.18	0.82	0.00	Hom

		a	Positional classification										b	Taxonomic classification							
			DFA					*glmnet*						DFA				*glmnet*			
Specimen	Taxon	Class	*DG*	*KW*	*PG*	*S*	Pred	*DG*	*KW*	*PG*	*S*	Pred	Class	Cerc	Hom	Plat	Pred	Cerc	Hom	Plat	Pred
NMNH 477333	*P. t. verus*	*KW*	0.00	1.00	0.00	0.00	*KW*	0.00	1.00	0.00	0.00	*KW*	Hom	0.00	1.00	0.00	Hom	0.00	1.00	0.00	Hom
NMNH 481803	*P. t. verus*	*KW*	0.00	0.95	0.05	0.00	*KW*	0.00	0.99	0.01	0.00	*KW*	Hom	0.01	0.99	0.00	Hom	0.02	0.98	0.00	Hom
NMNH 481804	*P. t. verus*	*KW*	0.00	0.99	0.01	0.00	*KW*	0.00	1.00	0.00	0.00	*KW*	Hom	0.02	0.98	0.00	Hom	0.02	0.98	0.00	Hom
UMMZ 76276	*P. t. verus*	*KW*	0.00	1.00	0.00	0.00	*KW*	0.00	1.00	0.00	0.00	*KW*	Hom	0.00	1.00	0.00	Hom	0.00	1.00	0.00	Hom
UMMZ 76277	*P. t. verus*	*KW*	0.00	0.93	0.00	0.07	*KW*	0.00	0.56	0.00	0.44	*KW*	Hom	0.00	1.00	0.00	Hom	0.00	1.00	0.00	Hom
MCZ 20041	*P. t. ellioti*	*KW*	0.00	0.91	0.08	0.00	*KW*	0.00	1.00	0.00	0.00	*KW*	Hom	0.07	0.93	0.00	Hom	0.14	0.86	0.00	Hom
MCZ 23163	*P. t. ellioti*	*KW*	0.00	1.00	0.00	0.00	*KW*	0.00	1.00	0.00	0.00	*KW*	Hom	0.00	1.00	0.00	Hom	0.00	1.00	0.00	Hom
MCZ 23167	*P. t. ellioti*	*KW*	0.00	1.00	0.00	0.00	*KW*	0.00	0.97	0.00	0.03	*KW*	Hom	0.00	1.00	0.00	Hom	0.00	1.00	0.00	Hom
MCZ 26849	*P. t. ellioti*	*KW*	0.00	0.88	0.06	0.05	*KW*	0.00	0.48	0.25	0.27	*KW*	Hom	0.01	0.99	0.00	Hom	0.01	0.99	0.00	Hom
UMMZ 167199	*P. t. ellioti*	*KW*	0.00	1.00	0.00	0.00	*KW*	0.00	1.00	0.00	0.00	*KW*	Hom	0.00	1.00	0.00	Hom	0.00	1.00	0.00	Hom
AMNH 86857	*P. paniscus*	*KW*	0.01	0.84	0.09	0.06	*KW*	0.00	0.93	0.00	0.06	*KW*	Hom	0.40	0.60	0.00	Hom	0.39	0.61	0.00	Hom
MCZ 38018	*P. paniscus*	*KW*	0.00	0.94	0.06	0.00	*KW*	0.00	1.00	0.00	0.00	*KW*	Hom	0.05	0.95	0.00	Hom	0.03	0.97	0.00	Hom
MCZ 38019	*P. paniscus*	*KW*	0.00	0.98	0.02	0.00	*KW*	0.00	1.00	0.00	0.00	*KW*	Hom	0.02	0.98	0.00	Hom	0.02	0.98	0.00	Hom
MCZ 38020	*P. paniscus*	*KW*	0.00	0.99	0.01	0.00	*KW*	0.00	1.00	0.00	0.00	*KW*	Hom	0.00	1.00	0.00	Hom	0.00	1.00	0.00	Hom
AMNH 54355	*G. gorilla*	*KW*	0.00	1.00	0.00	0.00	*KW*	0.00	1.00	0.00	0.00	*KW*	Hom	0.02	0.98	0.00	Hom	0.02	0.98	0.00	Hom
AMNH 54356	*G. gorilla*	*KW*	0.00	0.94	0.01	0.05	*KW*	0.00	0.22	0.05	0.73	***S***	Hom	0.04	0.95	0.01	Hom	0.08	0.92	0.00	Hom
AMNH 69398	*G. gorilla*	*KW*	0.01	0.62	0.37	0.00	*KW*	0.00	0.99	0.01	0.00	*KW*	Hom	0.80	0.20	0.00	**Cerc**	0.96	0.04	0.00	**Cerc**
AMNH 81651	*G. gorilla*	*KW*	0.00	1.00	0.00	0.00	*KW*	0.00	1.00	0.00	0.00	*KW*	Hom	0.00	1.00	0.00	Hom	0.00	1.00	0.00	Hom
AMNH 81652	*G. gorilla*	*KW*	0.00	1.00	0.00	0.00	*KW*	0.00	1.00	0.00	0.00	*KW*	Hom	0.00	1.00	0.00	Hom	0.00	1.00	0.00	Hom
AMNH 90289	*G. gorilla*	*KW*	0.00	1.00	0.00	0.00	*KW*	0.00	1.00	0.00	0.00	*KW*	Hom	0.00	1.00	0.00	Hom	0.00	1.00	0.00	Hom
AMNH 167335	*G. gorilla*	*KW*	0.00	1.00	0.00	0.00	*KW*	0.00	1.00	0.00	0.00	*KW*	Hom	0.00	1.00	0.00	Hom	0.00	1.00	0.00	Hom
AMNH 167337	*G. gorilla*	*KW*	0.00	0.95	0.00	0.05	*KW*	0.00	0.68	0.02	0.30	*KW*	Hom	0.01	0.99	0.00	Hom	0.01	0.99	0.00	Hom
AMNH 167338	*G. gorilla*	*KW*	0.00	1.00	0.00	0.00	*KW*	0.00	1.00	0.00	0.00	*KW*	Hom	0.00	1.00	0.00	Hom	0.00	1.00	0.00	Hom
AMNH 167339	*G. gorilla*	*KW*	0.00	1.00	0.00	0.00	*KW*	0.00	1.00	0.00	0.00	*KW*	Hom	0.00	1.00	0.00	Hom	0.00	1.00	0.00	Hom
AMNH 167340	*G. gorilla*	*KW*	0.00	1.00	0.00	0.00	*KW*	0.00	1.00	0.00	0.00	*KW*	Hom	0.01	0.99	0.00	Hom	0.02	0.98	0.00	Hom
AMNH 201471	*G. gorilla*	*KW*	0.00	1.00	0.00	0.00	*KW*	0.00	1.00	0.00	0.00	*KW*	Hom	0.00	1.00	0.00	Hom	0.00	1.00	0.00	Hom
AMNH 214103	*G. gorilla*	*KW*	0.00	1.00	0.00	0.00	*KW*	0.00	1.00	0.00	0.00	*KW*	Hom	0.00	1.00	0.00	Hom	0.00	1.00	0.00	Hom
MCZ 17684	*G. gorilla*	*KW*	0.00	1.00	0.00	0.00	*KW*	0.00	1.00	0.00	0.00	*KW*	Hom	0.00	1.00	0.00	Hom	0.00	1.00	0.00	Hom
MCZ 20038	*G. gorilla*	*KW*	0.00	0.93	0.07	0.00	*KW*	0.00	1.00	0.00	0.00	*KW*	Hom	0.33	0.67	0.00	Hom	0.25	0.75	0.00	Hom
MCZ 20039	*G. gorilla*	*KW*	0.00	0.99	0.01	0.00	*KW*	0.00	0.96	0.04	0.00	*KW*	Hom	0.01	0.99	0.00	Hom	0.01	0.99	0.00	Hom
MCZ 20043	*G. gorilla*	*KW*	0.00	1.00	0.00	0.00	*KW*	0.00	1.00	0.00	0.00	*KW*	Hom	0.00	1.00	0.00	Hom	0.00	1.00	0.00	Hom
MCZ 23160	*G. gorilla*	*KW*	0.00	1.00	0.00	0.00	*KW*	0.00	1.00	0.00	0.00	*KW*	Hom	0.00	1.00	0.00	Hom	0.00	1.00	0.00	Hom

		**a**	Positional classification											**b**	Taxonomic classification							
			DFA					*glmnet*						DFA				*glmnet*			
Specimen	Taxon	Class	*DG*	*KW*	*PG*	*S*	Pred	*DG*	*KW*	*PG*	*S*	Pred	Class	Cerc	Hom	Plat	Pred	Cerc	Hom	Plat	Pred
MCZ 23162	*G. gorilla*	*KW*	0.00	1.00	0.00	0.00	*KW*	0.00	1.00	0.00	0.00	*KW*	Hom	0.07	0.93	0.00	Hom	0.02	0.98	0.00	Hom
MCZ 26850	*G. gorilla*	*KW*	0.00	0.97	0.00	0.03	*KW*	0.00	0.95	0.01	0.03	*KW*	Hom	0.02	0.98	0.00	Hom	0.02	0.98	0.00	Hom
MCZ 29047	*G. gorilla*	*KW*	0.00	1.00	0.00	0.00	*KW*	0.00	1.00	0.00	0.00	*KW*	Hom	0.00	1.00	0.00	Hom	0.00	1.00	0.00	Hom
MCZ 29049	*G. gorilla*	*KW*	0.00	0.99	0.01	0.00	*KW*	0.00	1.00	0.00	0.00	*KW*	Hom	0.16	0.84	0.00	Hom	0.18	0.82	0.00	Hom
MCZ 37264	*G. gorilla*	*KW*	0.00	1.00	0.00	0.00	*KW*	0.00	1.00	0.00	0.00	*KW*	Hom	0.00	1.00	0.00	Hom	0.00	1.00	0.00	Hom
MCZ 38326	*G. gorilla*	*KW*	0.00	1.00	0.00	0.00	*KW*	0.00	1.00	0.00	0.00	*KW*	Hom	0.00	1.00	0.00	Hom	0.00	1.00	0.00	Hom
MCZ 57482	*G. gorilla*	*KW*	0.00	1.00	0.00	0.00	*KW*	0.00	0.87	0.00	0.13	*KW*	Hom	0.00	1.00	0.00	Hom	0.00	1.00	0.00	Hom
UMMZ 17886	*G. gorilla*	*KW*	0.00	1.00	0.00	0.00	*KW*	0.00	1.00	0.00	0.00	*KW*	Hom	0.00	1.00	0.00	Hom	0.00	1.00	0.00	Hom
AMNH 54089	*G. beringei*	*KW*	0.00	1.00	0.00	0.00	*KW*	0.00	1.00	0.00	0.00	*KW*	Hom	0.00	1.00	0.00	Hom	0.00	1.00	0.00	Hom
AMNH 54090	*G. beringei*	*KW*	0.00	0.84	0.16	0.00	*KW*	0.00	0.91	0.09	0.00	*KW*	Hom	0.05	0.95	0.00	Hom	0.17	0.83	0.00	Hom
AMNH 54091	*G. beringei*	*KW*	0.02	0.83	0.15	0.00	*KW*	0.01	0.85	0.14	0.00	*KW*	Hom	0.09	0.91	0.00	Hom	0.19	0.81	0.00	Hom
AMNH 115609	*G. beringei*	*KW*	0.00	1.00	0.00	0.00	*KW*	0.00	1.00	0.00	0.00	*KW*	Hom	0.00	1.00	0.00	Hom	0.00	1.00	0.00	Hom
NMNH 395636	*G. beringei*	*KW*	0.00	1.00	0.00	0.00	*KW*	0.00	1.00	0.00	0.00	*KW*	Hom	0.00	1.00	0.00	Hom	0.01	0.99	0.00	Hom
NMNH 396934	*G. beringei*	*KW*	0.00	0.94	0.04	0.02	*KW*	0.00	0.98	0.02	0.00	*KW*	Hom	0.05	0.95	0.00	Hom	0.05	0.95	0.00	Hom
NMNH 396935	*G. beringei*	*KW*	0.00	0.97	0.01	0.03	*KW*	0.00	0.95	0.03	0.03	*KW*	Hom	0.00	1.00	0.00	Hom	0.01	0.99	0.00	Hom
NMNH 396937	*G. beringei*	*KW*	0.00	1.00	0.00	0.00	*KW*	0.00	1.00	0.00	0.00	*KW*	Hom	0.00	1.00	0.00	Hom	0.00	1.00	0.00	Hom
NMNH 397351	*G. beringei*	*KW*	0.00	1.00	0.00	0.00	*KW*	0.00	1.00	0.00	0.00	*KW*	Hom	0.00	1.00	0.00	Hom	0.00	1.00	0.00	Hom
NMNH 545041	*G. beringei*	*KW*	0.00	0.99	0.01	0.00	*KW*	0.00	1.00	0.00	0.00	*KW*	Hom	0.00	1.00	0.00	Hom	0.01	0.99	0.00	Hom
MCZ 23182	*G. beringei*	*KW*	0.00	1.00	0.00	0.00	*KW*	0.00	1.00	0.00	0.00	*KW*	Hom	0.00	1.00	0.00	Hom	0.00	1.00	0.00	Hom
MCZ 38017	*G. beringei*	*KW*	0.00	1.00	0.00	0.00	*KW*	0.00	1.00	0.00	0.00	*KW*	Hom	0.00	1.00	0.00	Hom	0.00	1.00	0.00	Hom
AMNH 28252	*P. pygmaeus*	*S*	0.00	0.96	0.03	0.01	***KW***	0.00	0.48	0.14	0.39	***KW***	Hom	0.00	1.00	0.00	Hom	0.01	0.99	0.00	Hom
AMNH 28253	*P. pygmaeus*	*S*	0.00	0.97	0.01	0.02	***KW***	0.00	0.38	0.01	0.61	*S*	Hom	0.00	1.00	0.00	Hom	0.01	0.99	0.00	Hom
NMNH 145301	*P. pygmaeus*	*S*	0.00	0.90	0.00	0.10	***KW***	0.00	0.26	0.00	0.74	*S*	Hom	0.00	1.00	0.00	Hom	0.00	1.00	0.00	Hom
NMNH 145302	*P. pygmaeus*	*S*	0.00	0.63	0.00	0.37	***KW***	0.00	0.00	0.00	1.00	*S*	Hom	0.00	1.00	0.00	Hom	0.00	1.00	0.00	Hom
NMNH 145304	*P. pygmaeus*	*S*	0.00	0.08	0.00	0.92	*S*	0.00	0.00	0.00	1.00	*S*	Hom	0.00	1.00	0.00	Hom	0.00	1.00	0.00	Hom
NMNH 145305	*P. pygmaeus*	*S*	0.00	0.00	0.00	1.00	*S*	0.00	0.00	0.00	1.00	*S*	Hom	0.00	1.00	0.00	Hom	0.00	1.00	0.00	Hom
NMNH 145308	*P. pygmaeus*	*S*	0.00	0.92	0.00	0.08	***KW***	0.00	0.95	0.00	0.05	***KW***	Hom	0.00	1.00	0.00	Hom	0.00	1.00	0.00	Hom
NMNH 145309	*P. pygmaeus*	*S*	0.00	0.15	0.00	0.85	*S*	0.00	0.00	0.00	1.00	*S*	Hom	0.00	1.00	0.00	Hom	0.00	1.00	0.00	Hom
NMNH 145310	*P. pygmaeus*	*S*	0.00	0.01	0.00	0.99	*S*	0.00	0.00	0.00	1.00	*S*	Hom	0.00	1.00	0.00	Hom	0.00	1.00	0.00	Hom
MCZ 37362	*P. pygmaeus*	*S*	0.00	0.22	0.20	0.58	*S*	0.00	0.00	0.27	0.73	*S*	Hom	0.01	0.99	0.00	Hom	0.02	0.98	0.00	Hom
MCZ 37363	*P. pygmaeus*	*S*	0.00	0.00	0.00	1.00	*S*	0.00	0.00	0.00	1.00	*S*	Hom	0.00	1.00	0.00	Hom	0.00	1.00	0.00	Hom
MCZ 37364	*P. pygmaeus*	*S*	0.00	0.00	0.00	1.00	*S*	0.00	0.00	0.00	1.00	*S*	Hom	0.00	1.00	0.00	Hom	0.00	1.00	0.00	Hom

		**a** Positional classification												**b** Taxonomic classification								
			DFA					*glmnet*						DFA				*glmnet*			
Specimen	Taxon	Class	*DG*	*KW*	*PG*	*S*	Pred	*DG*	*KW*	*PG*	*S*	Pred	Class	Cerc	Hom	Plat	Pred	Cerc	Hom	Plat	Pred
MCZ 37365	*P. pygmaeus*	*S*	0.01	0.01	0.59	0.39	***PG***	0.01	0.00	0.27	0.71	*S*	Hom	0.91	0.09	0.00	**Cerc**	0.99	0.01	0.00	**Cerc**
NMNH 142170	*P. pygmaeus*	*S*	0.00	0.00	0.00	1.00	*S*	0.00	0.00	0.00	1.00	*S*	Hom	0.00	1.00	0.00	Hom	0.01	0.99	0.00	Hom
NMNH 153805	*P. pygmaeus*	*S*	0.00	0.23	0.00	0.77	*S*	0.00	0.04	0.01	0.94	*S*	Hom	0.00	1.00	0.00	Hom	0.00	1.00	0.00	Hom
NMNH 153823	*P. pygmaeus*	*S*	0.00	0.01	0.00	0.99	*S*	0.00	0.00	0.00	1.00	*S*	Hom	0.00	1.00	0.00	Hom	0.00	1.00	0.00	Hom
AMNH 61586	*P. pygmaeus*	*S*	0.00	0.06	0.00	0.94	*S*	0.00	0.00	0.00	1.00	*S*	Hom	0.00	1.00	0.00	Hom	0.00	1.00	0.00	Hom
AMNH 202511	*P. pygmaeus*	*S*	0.00	0.04	0.00	0.96	*S*	0.00	0.00	0.00	1.00	*S*	Hom	0.00	1.00	0.00	Hom	0.00	1.00	0.00	Hom
AMNH 239847	*P. pygmaeus*	*S*	0.00	0.01	0.00	0.99	*S*	0.00	0.00	0.00	1.00	*S*	Hom	0.00	1.00	0.00	Hom	0.00	1.00	0.00	Hom
MCZ 50960	*P. abelii*	*S*	0.00	0.04	0.00	0.96	*S*	0.00	0.00	0.00	1.00	*S*	Hom	0.00	1.00	0.00	Hom	0.00	1.00	0.00	Hom
CMNH HTB1030	*P. abelii*	*S*	0.00	0.00	0.00	1.00	*S*	0.00	0.00	0.00	1.00	*S*	Hom	0.00	1.00	0.00	Hom	0.00	1.00	0.00	Hom
CMNH HTB1055	*P. abelii*	*S*	0.00	0.00	0.00	1.00	*S*	0.00	0.00	0.00	1.00	*S*	Hom	0.00	1.00	0.00	Hom	0.00	1.00	0.00	Hom
CMNH HTB1168	*P. abelii*	*S*	0.00	0.00	0.00	1.00	*S*	0.00	0.00	0.00	1.00	*S*	Hom	0.00	1.00	0.00	Hom	0.00	1.00	0.00	Hom
CMNH HTB1444	*P. abelii*	*S*	0.00	0.00	0.00	1.00	*S*	0.00	0.00	0.00	1.00	*S*	Hom	0.00	1.00	0.00	Hom	0.00	1.00	0.00	Hom
NMNH 143590	*P. abelii*	*S*	0.00	0.01	0.00	0.99	*S*	0.00	0.00	0.00	1.00	*S*	Hom	0.00	1.00	0.00	Hom	0.00	1.00	0.00	Hom
NMNH 143593	*P. abelii*	*S*	0.00	0.23	0.08	0.68	*S*	0.03	0.04	0.73	0.20	***PG***	Hom	0.01	0.99	0.00	Hom	0.01	0.99	0.00	Hom
NMNH 143594	*P. abelii*	*S*	0.00	0.00	0.00	1.00	*S*	0.00	0.00	0.00	1.00	*S*	Hom	0.00	1.00	0.00	Hom	0.00	1.00	0.00	Hom
NMNH 143596	*P. abelii*	*S*	0.00	0.01	0.00	0.99	*S*	0.00	0.00	0.00	1.00	*S*	Hom	0.00	1.00	0.00	Hom	0.00	1.00	0.00	Hom
NMNH 143597	*P. abelii*	*S*	0.00	0.02	0.00	0.98	*S*	0.00	0.00	0.00	1.00	*S*	Hom	0.00	1.00	0.00	Hom	0.00	1.00	0.00	Hom
NMNH 143598	*P. abelii*	*S*	0.00	0.00	0.00	1.00	*S*	0.00	0.00	0.00	1.00	*S*	Hom	0.00	1.00	0.00	Hom	0.00	1.00	0.00	Hom
NMNH 143600	*P. abelii*	*S*	0.00	0.00	0.00	1.00	*S*	0.00	0.00	0.00	1.00	*S*	Hom	0.00	1.00	0.00	Hom	0.00	1.00	0.00	Hom
NMNH 143601	*P. abelii*	*S*	0.00	0.00	0.00	1.00	*S*	0.00	0.00	0.00	1.00	*S*	Hom	0.30	0.70	0.00	Hom	0.44	0.56	0.00	Hom
NMNH 143602	*P. abelii*	*S*	0.00	0.00	0.00	1.00	*S*	0.00	0.00	0.00	1.00	*S*	Hom	0.00	1.00	0.00	Hom	0.00	1.00	0.00	Hom
NMNH 270807	*P. abelii*	*S*	0.00	0.00	0.00	1.00	*S*	0.00	0.00	0.00	1.00	*S*	Hom	0.04	0.96	0.00	Hom	0.09	0.91	0.00	Hom
AMNH 80068	*Hoolock*	*S*	0.01	0.00	0.03	0.96	*S*	0.03	0.00	0.48	0.49	*S*	Hom	0.00	1.00	0.00	Hom	0.02	0.97	0.00	Hom
AMNH 83418	*Hoolock*	*S*	0.00	0.00	0.00	1.00	*S*	0.00	0.00	0.00	1.00	*S*	Hom	0.00	1.00	0.00	Hom	0.01	0.99	0.00	Hom
AMNH 83420	*Hoolock*	*S*	0.00	0.00	0.00	1.00	*S*	0.00	0.00	0.00	1.00	*S*	Hom	0.00	1.00	0.00	Hom	0.00	1.00	0.00	Hom
AMNH 83423	*Hoolock*	*S*	0.00	0.00	0.00	1.00	*S*	0.00	0.00	0.00	1.00	*S*	Hom	0.00	1.00	0.00	Hom	0.00	1.00	0.00	Hom
AMNH 83425	*Hoolock*	*S*	0.00	0.00	0.00	1.00	*S*	0.00	0.00	0.00	1.00	*S*	Hom	0.01	0.99	0.00	Hom	0.09	0.90	0.02	Hom
AMNH 112676	*Hoolock*	*S*	0.00	0.00	0.00	1.00	*S*	0.00	0.00	0.00	1.00	*S*	Hom	0.00	1.00	0.00	Hom	0.00	1.00	0.00	Hom
AMNH 112720	*Hoolock*	*S*	0.00	0.00	0.00	1.00	*S*	0.00	0.00	0.00	1.00	*S*	Hom	0.00	1.00	0.00	Hom	0.00	1.00	0.00	Hom
MCZ 37378	*H. muelleri*	*S*	0.00	0.00	0.00	1.00	*S*	0.00	0.00	0.00	1.00	*S*	Hom	0.00	1.00	0.00	Hom	0.00	1.00	0.00	Hom
MCZ 37380	*H. muelleri*	*S*	0.00	0.00	0.00	1.00	*S*	0.00	0.00	0.00	1.00	*S*	Hom	0.00	1.00	0.00	Hom	0.00	1.00	0.00	Hom
MCZ 37381	*H. muelleri*	*S*	0.00	0.00	0.00	1.00	*S*	0.00	0.00	0.00	1.00	*S*	Hom	0.05	0.95	0.00	Hom	0.05	0.95	0.00	Hom

		a	Positional classification										b	Taxonomic classification							
			DFA					*glmnet*						DFA				*glmnet*			
Specimen	Taxon	Class	*DG*	*KW*	*PG*	*S*	Pred	*DG*	*KW*	*PG*	*S*	Pred	Class	Cerc	Hom	Plat	Pred	Cerc	Hom	Plat	Pred
MCZ 37383	*H. muelleri*	*S*	0.00	0.00	0.00	1.00	*S*	0.00	0.00	0.00	1.00	*S*	Hom	0.00	1.00	0.00	Hom	0.00	1.00	0.00	Hom
MCZ 41417	*H. lar*	*S*	0.00	0.00	0.00	1.00	*S*	0.00	0.00	0.00	1.00	*S*	Hom	0.00	1.00	0.00	Hom	0.01	0.97	0.02	Hom
MCZ 41524	*H. lar*	*S*	0.00	0.00	0.00	1.00	*S*	0.00	0.00	0.00	1.00	*S*	Hom	0.00	1.00	0.00	Hom	0.00	1.00	0.00	Hom
MCZ 41525	*H. lar*	*S*	0.00	0.00	0.00	1.00	*S*	0.00	0.00	0.00	1.00	*S*	Hom	0.01	0.94	0.05	Hom	0.01	0.21	0.78	**Plat**
MCZ 41526	*H. lar*	*S*	0.00	0.00	0.00	1.00	*S*	0.00	0.00	0.00	1.00	*S*	Hom	0.00	1.00	0.00	Hom	0.00	1.00	0.00	Hom
MCZ 41527	*H. lar*	*S*	0.00	0.00	0.00	1.00	*S*	0.00	0.00	0.00	1.00	*S*	Hom	0.01	0.98	0.01	Hom	0.03	0.84	0.13	Hom
MCZ 41529	*H. lar*	*S*	0.00	0.00	0.00	1.00	*S*	0.00	0.00	0.00	1.00	*S*	Hom	0.00	1.00	0.00	Hom	0.00	1.00	0.00	Hom
MCZ 41530	*H. lar*	*S*	0.00	0.00	0.00	1.00	*S*	0.00	0.00	0.00	1.00	*S*	Hom	0.00	0.99	0.01	Hom	0.00	0.90	0.10	Hom
MCZ 41531	*H. lar*	*S*	0.00	0.00	0.00	1.00	*S*	0.00	0.00	0.00	1.00	*S*	Hom	0.01	0.99	0.00	Hom	0.01	0.99	0.00	Hom
MCZ 41532	*H. lar*	*S*	0.00	0.00	0.00	1.00	*S*	0.00	0.00	0.00	1.00	*S*	Hom	0.00	1.00	0.00	Hom	0.00	0.99	0.00	Hom
MCZ 41534	*H. lar*	*S*	0.00	0.00	0.00	1.00	*S*	0.00	0.00	0.00	1.00	*S*	Hom	0.17	0.80	0.03	Hom	0.28	0.47	0.25	Hom
MCZ 41536	*H. lar*	*S*	0.00	0.00	0.00	1.00	*S*	0.00	0.00	0.00	1.00	*S*	Hom	0.01	0.99	0.00	Hom	0.01	0.99	0.00	Hom
AMNH 43063	*H. lar*	*S*	0.00	0.00	0.00	1.00	*S*	0.00	0.00	0.00	1.00	*S*	Hom	0.03	0.97	0.00	Hom	0.04	0.96	0.00	Hom
MCZ 41565	*H. lar*	*S*	0.00	0.00	0.00	1.00	*S*	0.00	0.00	0.00	1.00	*S*	Hom	0.05	0.89	0.06	Hom	0.09	0.78	0.13	Hom
UMMZ 160908	*H. lar*	*S*	0.00	0.00	0.00	1.00	*S*	0.00	0.00	0.00	1.00	*S*	Hom	0.01	0.99	0.00	Hom	0.01	0.99	0.00	Hom
UMMZ 160909	*H. lar*	*S*	0.00	0.00	0.00	1.00	*S*	0.00	0.00	0.00	1.00	*S*	Hom	0.01	0.99	0.00	Hom	0.01	0.99	0.00	Hom
AMNH 106581	*Symphalangus*	*S*	0.00	0.00	0.00	1.00	*S*	0.00	0.00	0.00	1.00	*S*	Hom	0.01	0.99	0.00	Hom	0.01	0.99	0.00	Hom
AMNH 106583	*Symphalangus*	*S*	0.00	0.00	0.00	1.00	*S*	0.00	0.00	0.00	1.00	*S*	Hom	0.00	1.00	0.00	Hom	0.00	1.00	0.00	Hom
MCZ 27867	*Symphalangus*	*S*	0.00	0.00	0.00	1.00	*S*	0.00	0.00	0.00	1.00	*S*	Hom	0.00	1.00	0.00	Hom	0.01	0.99	0.00	Hom
AMNH 51380	*Papio*	*DG*	0.98	0.00	0.02	0.00	*DG*	0.97	0.00	0.03	0.00	*DG*	Cerc	0.15	0.85	0.00	**Hom**	0.20	0.80	0.00	**Hom**
AMNH 52668	*Papio*	*DG*	0.56	0.00	0.44	0.00	*DG*	0.19	0.00	0.81	0.00	***PG***	Cerc	1.00	0.00	0.00	Cerc	1.00	0.00	0.00	Cerc
AMNH 52676	*Papio*	*DG*	0.93	0.00	0.07	0.00	*DG*	0.96	0.00	0.04	0.00	*DG*	Cerc	1.00	0.00	0.00	Cerc	1.00	0.00	0.00	Cerc
AMNH 82097	*Papio*	*DG*	0.33	0.00	0.67	0.00	***PG***	0.22	0.00	0.78	0.00	***PG***	Cerc	1.00	0.00	0.00	Cerc	1.00	0.00	0.00	Cerc
AMNH 187369	*Papio*	*DG*	0.50	0.00	0.50	0.00	***PG***	0.07	0.00	0.93	0.00	***PG***	Cerc	0.99	0.00	0.01	Cerc	1.00	0.00	0.00	Cerc
MCZ 15378	*Papio*	*DG*	0.33	0.00	0.67	0.00	***PG***	0.59	0.00	0.41	0.00	*DG*	Cerc	1.00	0.00	0.00	Cerc	1.00	0.00	0.00	Cerc
NMNH 236976	*Papio*	*DG*	0.53	0.00	0.47	0.00	*DG*	0.69	0.00	0.31	0.00	*DG*	Cerc	1.00	0.00	0.00	Cerc	1.00	0.00	0.00	Cerc
NMNH 239743	*Papio*	*DG*	0.25	0.00	0.75	0.00	***PG***	0.29	0.00	0.71	0.00	***PG***	Cerc	0.97	0.03	0.00	Cerc	0.98	0.02	0.00	Cerc
NMNH 384223	*Papio*	*DG*	0.95	0.00	0.05	0.00	*DG*	0.99	0.00	0.01	0.00	*DG*	Cerc	1.00	0.00	0.00	Cerc	1.00	0.00	0.00	Cerc
NMNH 384227	*Papio*	*DG*	0.82	0.00	0.18	0.00	*DG*	0.75	0.00	0.25	0.00	*DG*	Cerc	0.98	0.02	0.00	Cerc	0.99	0.01	0.00	Cerc
NMNH 384228	*Papio*	*DG*	0.61	0.00	0.39	0.00	*DG*	0.71	0.00	0.29	0.00	*DG*	Cerc	0.97	0.03	0.00	Cerc	0.98	0.02	0.00	Cerc
NMNH 384229	*Papio*	*DG*	0.71	0.00	0.29	0.00	*DG*	0.43	0.00	0.57	0.00	***PG***	Cerc	1.00	0.00	0.00	Cerc	1.00	0.00	0.00	Cerc
NMNH 384234	*Papio*	*DG*	0.11	0.01	0.88	0.00	***PG***	0.07	0.01	0.91	0.00	***PG***	Cerc	0.99	0.01	0.00	Cerc	0.99	0.01	0.00	Cerc

		**a** Positional classification												**b** Taxonomic classification								
			DFA					*glmnet*						DFA				*glmnet*			
Specimen	Taxon	Class	*DG*	*KW*	*PG*	*S*	Pred	*DG*	*KW*	*PG*	*S*	Pred	Class	Cerc	Hom	Plat	Pred	Cerc	Hom	Plat	Pred
NMNH 384235	*Papio*	*DG*	0.25	0.00	0.75	0.00	***PG***	0.10	0.00	0.90	0.00	***PG***	Cerc	0.98	0.00	0.02	Cerc	1.00	0.00	0.00	Cerc
AMNH 52596	*Lophocebus*	*PG*	0.43	0.00	0.57	0.00	*PG*	0.20	0.00	0.80	0.00	*PG*	Cerc	1.00	0.00	0.00	Cerc	1.00	0.00	0.00	Cerc
AMNH 52609	*Lophocebus*	*PG*	0.24	0.02	0.74	0.00	*PG*	0.25	0.00	0.75	0.00	*PG*	Cerc	0.55	0.45	0.00	Cerc	0.72	0.28	0.00	Cerc
AMNH 52627	*Lophocebus*	*PG*	0.29	0.00	0.71	0.00	*PG*	0.11	0.00	0.89	0.00	*PG*	Cerc	0.79	0.00	0.21	Cerc	1.00	0.00	0.00	Cerc
MCZ 37928	*Lophocebus*	*PG*	0.75	0.00	0.25	0.00	***DG***	0.66	0.01	0.32	0.00	***DG***	Cerc	1.00	0.00	0.00	Cerc	1.00	0.00	0.00	Cerc
AMNH 167678	*Lophocebus*	*PG*	0.10	0.00	0.90	0.00	*PG*	0.06	0.00	0.94	0.00	*PG*	Cerc	1.00	0.00	0.00	Cerc	1.00	0.00	0.00	Cerc
NMNH 578579	*Lophocebus*	*PG*	0.19	0.00	0.81	0.00	*PG*	0.06	0.00	0.94	0.00	*PG*	Cerc	0.95	0.00	0.05	Cerc	1.00	0.00	0.00	Cerc
AMNH 89361	*Mandrillus*	*DG*	0.21	0.00	0.75	0.04	***PG***	0.24	0.00	0.73	0.03	***PG***	Cerc	1.00	0.00	0.00	Cerc	1.00	0.00	0.00	Cerc
AMNH 89362	*Mandrillus*	*DG*	0.87	0.00	0.13	0.00	*DG*	0.90	0.00	0.10	0.00	*DG*	Cerc	0.93	0.07	0.00	Cerc	0.92	0.08	0.00	Cerc
AMNH 89364	*Mandrillus*	*DG*	0.97	0.00	0.03	0.00	*DG*	0.96	0.00	0.04	0.00	*DG*	Cerc	1.00	0.00	0.00	Cerc	1.00	0.00	0.00	Cerc
AMNH 89367	*Mandrillus*	*DG*	0.98	0.00	0.02	0.00	*DG*	1.00	0.00	0.00	0.00	*DG*	Cerc	0.98	0.02	0.00	Cerc	0.99	0.01	0.00	Cerc
AMNH 170364	*Mandrillus*	*DG*	0.71	0.00	0.29	0.00	*DG*	0.77	0.00	0.23	0.00	*DG*	Cerc	0.89	0.11	0.00	Cerc	0.93	0.07	0.00	Cerc
AMNH 170366	*Mandrillus*	*DG*	0.98	0.00	0.02	0.00	*DG*	0.99	0.00	0.01	0.00	*DG*	Cerc	0.66	0.34	0.00	Cerc	0.76	0.24	0.00	Cerc
MCZ 34090	*Mandrillus*	*DG*	0.86	0.00	0.14	0.00	*DG*	0.91	0.00	0.09	0.00	*DG*	Cerc	0.15	0.85	0.00	**Hom**	0.17	0.83	0.00	**Hom**
MCZ 34137	*Mandrillus*	*DG*	0.31	0.04	0.65	0.00	***PG***	0.16	0.01	0.84	0.00	***PG***	Cerc	0.94	0.06	0.00	Cerc	0.89	0.11	0.00	Cerc
MCZ 34177	*Mandrillus*	*DG*	0.79	0.00	0.21	0.00	*DG*	0.65	0.01	0.34	0.00	*DG*	Cerc	1.00	0.00	0.00	Cerc	1.00	0.00	0.00	Cerc
AMNH 52634	*Cercocebus*	*PG*	0.98	0.00	0.02	0.00	***DG***	0.98	0.00	0.02	0.00	***DG***	Cerc	0.99	0.01	0.00	Cerc	0.99	0.01	0.00	Cerc
AMNH 81250	*Cercocebus*	*PG*	0.65	0.00	0.35	0.00	***DG***	0.47	0.00	0.53	0.00	*PG*	Cerc	1.00	0.00	0.00	Cerc	1.00	0.00	0.00	Cerc
AMNH 103654	*Macaca*	*PG*	0.06	0.66	0.29	0.00	***KW***	0.01	0.26	0.73	0.00	*PG*	Cerc	0.31	0.69	0.00	**Hom**	0.22	0.78	0.00	**Hom**
AMNH 103659	*Macaca*	*PG*	0.13	0.00	0.86	0.00	*PG*	0.09	0.00	0.91	0.00	*PG*	Cerc	0.99	0.01	0.00	Cerc	0.99	0.01	0.00	Cerc
AMNH 175460	*Macaca*	*PG*	0.01	0.00	0.99	0.00	*PG*	0.01	0.00	0.99	0.00	*PG*	Cerc	1.00	0.00	0.00	Cerc	1.00	0.00	0.00	Cerc
MCZ 35626	*Macaca*	*PG*	0.08	0.02	0.90	0.00	*PG*	0.02	0.00	0.98	0.00	*PG*	Cerc	0.92	0.08	0.00	Cerc	0.96	0.04	0.00	Cerc
MCZ 35629	*Macaca*	*PG*	0.13	0.00	0.87	0.00	*PG*	0.05	0.00	0.95	0.00	*PG*	Cerc	1.00	0.00	0.00	Cerc	1.00	0.00	0.00	Cerc
MCZ 35652	*Macaca*	*PG*	0.36	0.42	0.22	0.00	***KW***	0.01	0.97	0.02	0.00	***KW***	Cerc	0.59	0.41	0.00	Cerc	0.53	0.47	0.00	Cerc
MCZ 35658	*Macaca*	*PG*	0.22	0.02	0.76	0.00	*PG*	0.06	0.00	0.94	0.00	*PG*	Cerc	0.97	0.03	0.00	Cerc	0.98	0.02	0.00	Cerc
MCZ 35677	*Macaca*	*PG*	0.54	0.01	0.45	0.00	***DG***	0.14	0.01	0.85	0.00	*PG*	Cerc	0.93	0.07	0.00	Cerc	0.94	0.06	0.00	Cerc
MCZ 35681	*Macaca*	*PG*	0.15	0.01	0.83	0.00	*PG*	0.12	0.00	0.86	0.02	*PG*	Cerc	0.96	0.04	0.00	Cerc	0.97	0.03	0.00	Cerc
MCZ 35693	*Macaca*	*PG*	0.17	0.00	0.83	0.00	*PG*	0.04	0.00	0.96	0.00	*PG*	Cerc	0.99	0.00	0.01	Cerc	1.00	0.00	0.00	Cerc
MCZ 35694	*Macaca*	*PG*	0.80	0.03	0.17	0.00	***DG***	0.60	0.00	0.39	0.00	***DG***	Cerc	0.14	0.86	0.00	**Hom**	0.18	0.82	0.00	**Hom**
MCZ 35700	*Macaca*	*PG*	0.50	0.00	0.50	0.00	*PG*	0.20	0.00	0.80	0.00	*PG*	Cerc	0.97	0.03	0.00	Cerc	0.99	0.01	0.00	Cerc
MCZ 35701	*Macaca*	*PG*	0.00	0.11	0.89	0.00	*PG*	0.00	0.00	1.00	0.00	*PG*	Cerc	0.85	0.15	0.00	Cerc	0.89	0.11	0.00	Cerc
MCZ 35729	*Macaca*	*PG*	0.13	0.00	0.87	0.00	*PG*	0.04	0.00	0.96	0.00	*PG*	Cerc	1.00	0.00	0.00	Cerc	1.00	0.00	0.00	Cerc

		**a**	Positional classification										**b**	Taxonomic classification							
			DFA					*glmnet*						DFA				*glmnet*			
Specimen	Taxon	Class	*DG*	*KW*	*PG*	*S*	Pred	*DG*	*KW*	*PG*	*S*	Pred	Class	Cerc	Hom	Plat	Pred	Cerc	Hom	Plat	Pred
MCZ 35736	*Macaca*	*PG*	0.22	0.00	0.78	0.00	*PG*	0.12	0.00	0.88	0.00	*PG*	Cerc	1.00	0.00	0.00	Cerc	1.00	0.00	0.00	Cerc
UMMZ 130418	*Macaca*	*PG*	0.02	0.06	0.92	0.00	*PG*	0.00	0.00	1.00	0.00	*PG*	Cerc	0.79	0.21	0.00	Cerc	0.88	0.12	0.00	Cerc
UMMZ 161308	*Macaca*	*PG*	0.52	0.00	0.48	0.00	***DG***	0.24	0.00	0.76	0.00	*PG*	Cerc	0.98	0.02	0.00	Cerc	0.99	0.01	0.00	Cerc
UMMZ 56349	*Macaca*	*PG*	0.01	0.02	0.76	0.21	*PG*	0.01	0.00	0.20	0.79	***S***	Cerc	0.99	0.01	0.00	Cerc	0.99	0.01	0.00	Cerc
AMNH 34709	*Erythrocebus*	*DG*	0.47	0.00	0.53	0.00	***PG***	0.59	0.00	0.41	0.00	*DG*	Cerc	0.99	0.00	0.01	Cerc	1.00	0.00	0.00	Cerc
AMNH 34712	*Erythrocebus*	*DG*	0.28	0.00	0.72	0.00	***PG***	0.53	0.00	0.47	0.00	*DG*	Cerc	0.99	0.00	0.01	Cerc	1.00	0.00	0.00	Cerc
AMNH 34713	*Erythrocebus*	*DG*	0.80	0.00	0.20	0.00	*DG*	0.82	0.00	0.18	0.00	*DG*	Cerc	0.98	0.00	0.02	Cerc	1.00	0.00	0.00	Cerc
AMNH 34714	*Erythrocebus*	*DG*	0.63	0.00	0.37	0.00	*DG*	0.32	0.00	0.68	0.00	***PG***	Cerc	0.93	0.00	0.07	Cerc	0.99	0.00	0.01	Cerc
NMNH 257013	*Erythrocebus*	*DG*	0.68	0.00	0.32	0.00	*DG*	0.78	0.00	0.22	0.00	*DG*	Cerc	1.00	0.00	0.00	Cerc	1.00	0.00	0.00	Cerc
NMNH 399317	*Erythrocebus*	*DG*	0.40	0.00	0.60	0.00	***PG***	0.17	0.00	0.83	0.00	***PG***	Cerc	0.99	0.00	0.01	Cerc	1.00	0.00	0.00	Cerc
NMNH 538311	*Erythrocebus*	*DG*	0.52	0.00	0.48	0.00	*DG*	0.87	0.00	0.13	0.00	*DG*	Cerc	1.00	0.00	0.00	Cerc	1.00	0.00	0.00	Cerc
AMNH 52368	*Cercopithecus*	*PG*	0.87	0.00	0.13	0.00	***DG***	0.75	0.00	0.25	0.00	***DG***	Cerc	0.99	0.00	0.01	Cerc	1.00	0.00	0.00	Cerc
AMNH 52398	*Cercopithecus*	*PG*	0.78	0.00	0.22	0.00	***DG***	0.80	0.00	0.20	0.00	***DG***	Cerc	1.00	0.00	0.00	Cerc	1.00	0.00	0.00	Cerc
AMNH 52401	*Cercopithecus*	*PG*	0.31	0.00	0.69	0.00	*PG*	0.20	0.00	0.80	0.00	*PG*	Cerc	0.99	0.00	0.01	Cerc	1.00	0.00	0.00	Cerc
AMNH 52410	*Cercopithecus*	*PG*	0.51	0.00	0.49	0.00	***DG***	0.29	0.00	0.71	0.00	*PG*	Cerc	1.00	0.00	0.00	Cerc	1.00	0.00	0.00	Cerc
AMNH 82411	*Cercopithecus*	*PG*	0.14	0.00	0.86	0.00	*PG*	0.07	0.00	0.93	0.00	*PG*	Cerc	0.98	0.00	0.02	Cerc	1.00	0.00	0.00	Cerc
AMNH 82412	*Cercopithecus*	*PG*	0.44	0.00	0.56	0.00	*PG*	0.29	0.00	0.71	0.00	*PG*	Cerc	1.00	0.00	0.00	Cerc	1.00	0.00	0.00	Cerc
AMNH 82415	*Cercopithecus*	*PG*	0.20	0.00	0.80	0.00	*PG*	0.66	0.00	0.34	0.00	***DG***	Cerc	0.99	0.01	0.00	Cerc	0.99	0.01	0.00	Cerc
MCZ 37930	*Cercopithecus*	*PG*	0.71	0.00	0.29	0.00	***DG***	0.39	0.00	0.61	0.00	*PG*	Cerc	1.00	0.00	0.00	Cerc	1.00	0.00	0.00	Cerc
MCZ 37934	*Cercopithecus*	*PG*	0.15	0.00	0.85	0.00	*PG*	0.12	0.00	0.88	0.00	*PG*	Cerc	1.00	0.00	0.00	Cerc	1.00	0.00	0.00	Cerc
MCZ 38079	*Cercopithecus*	*PG*	0.84	0.00	0.16	0.00	***DG***	0.91	0.00	0.09	0.00	***DG***	Cerc	1.00	0.00	0.00	Cerc	1.00	0.00	0.00	Cerc
UMMZ 39508	*Cercopithecus*	*PG*	0.65	0.00	0.35	0.00	***DG***	0.72	0.00	0.28	0.00	***DG***	Cerc	0.97	0.03	0.00	Cerc	0.98	0.02	0.00	Cerc
AMNH 27711	*Colobus*	*PG*	0.00	0.02	0.98	0.00	*PG*	0.00	0.02	0.98	0.00	*PG*	Cerc	0.97	0.00	0.03	Cerc	1.00	0.00	0.00	Cerc
AMNH 99468	*Colobus*	*PG*	0.01	0.26	0.59	0.14	*PG*	0.00	0.02	0.61	0.36	*PG*	Cerc	0.16	0.09	0.75	**Plat**	0.50	0.37	0.13	Cerc
NMNH 452621	*Colobus*	*PG*	0.01	0.00	0.99	0.00	*PG*	0.00	0.00	1.00	0.00	*PG*	Cerc	0.14	0.00	0.86	**Plat**	0.65	0.00	0.35	Cerc
AMNH 52223	*Colobus*	*PG*	0.00	0.00	1.00	0.00	*PG*	0.00	0.00	1.00	0.00	*PG*	Cerc	0.79	0.00	0.21	Cerc	0.61	0.00	0.39	Cerc
AMNH 52229	*Colobus*	*PG*	0.00	0.01	0.99	0.00	*PG*	0.00	0.00	1.00	0.00	*PG*	Cerc	0.61	0.00	0.39	Cerc	0.95	0.00	0.05	Cerc
AMNH 52240	*Colobus*	*PG*	0.01	0.00	0.99	0.00	*PG*	0.00	0.00	1.00	0.00	*PG*	Cerc	0.45	0.00	0.55	**Plat**	0.87	0.00	0.13	Cerc
AMNH 52241	*Colobus*	*PG*	0.00	0.01	0.99	0.00	*PG*	0.00	0.00	1.00	0.00	*PG*	Cerc	0.76	0.00	0.24	Cerc	0.97	0.00	0.03	Cerc
AMNH 52248	*Colobus*	*PG*	0.02	0.00	0.98	0.00	*PG*	0.00	0.00	1.00	0.00	*PG*	Cerc	0.13	0.00	0.87	**Plat**	0.47	0.00	0.53	**Plat**
AMNH 187392	*Colobus*	*PG*	0.00	0.02	0.98	0.00	*PG*	0.00	0.01	0.99	0.00	*PG*	Cerc	0.13	0.00	0.87	**Plat**	0.70	0.00	0.30	Cerc
AMNH 52278	*Procolobus*	*PG*	0.02	0.00	0.98	0.00	*PG*	0.01	0.00	0.99	0.00	*PG*	Cerc	0.97	0.00	0.03	Cerc	0.97	0.00	0.03	Cerc

		**a** Positional classification											**b** Taxonomic classification								
			DFA					*glmnet*						DFA				*glmnet*			
Specimen	Taxon	Class	*DG*	*KW*	*PG*	*S*	Pred	*DG*	*KW*	*PG*	*S*	Pred	Class	Cerc	Hom	Plat	Pred	Cerc	Hom	Plat	Pred
AMNH 52287	*Procolobus*	*PG*	0.02	0.02	0.95	0.00	*PG*	0.04	0.00	0.96	0.01	*PG*	Cerc	0.80	0.16	0.04	Cerc	0.94	0.06	0.00	Cerc
AMNH 52298	*Procolobus*	*PG*	0.08	0.00	0.92	0.00	*PG*	0.16	0.00	0.84	0.00	*PG*	Cerc	0.77	0.00	0.23	Cerc	0.93	0.00	0.07	Cerc
AMNH 52303	*Procolobus*	*PG*	0.02	0.00	0.98	0.00	*PG*	0.03	0.00	0.97	0.00	*PG*	Cerc	0.99	0.00	0.01	Cerc	1.00	0.00	0.00	Cerc
AMNH 52334	*Procolobus*	*PG*	0.01	0.11	0.54	0.34	*PG*	0.01	0.00	0.76	0.23	*PG*	Cerc	0.24	0.76	0.00	**Hom**	0.32	0.68	0.00	**Hom**
MCZ 37943	*Procolobus*	*PG*	0.00	0.81	0.19	0.00	***KW***	0.00	0.35	0.65	0.00	*PG*	Cerc	0.03	0.96	0.00	**Hom**	0.02	0.98	0.00	**Hom**
AMNH 54279	*Procolobus*	*PG*	0.01	0.02	0.97	0.00	*PG*	0.00	0.00	1.00	0.00	*PG*	Cerc	0.82	0.07	0.11	Cerc	0.95	0.03	0.02	Cerc
AMNH 86709	*Procolobus*	*PG*	0.01	0.00	0.99	0.00	*PG*	0.01	0.10	0.89	0.00	*PG*	Cerc	0.99	0.00	0.01	Cerc	0.99	0.00	0.01	Cerc
MCZ 37931	*Procolobus*	*PG*	0.00	0.00	1.00	0.00	*PG*	0.00	0.00	1.00	0.00	*PG*	Cerc	0.53	0.00	0.47	Cerc	0.15	0.00	0.85	**Plat**
MCZ 37932	*Procolobus*	*PG*	0.13	0.00	0.86	0.00	*PG*	0.20	0.00	0.80	0.00	*PG*	Cerc	0.98	0.01	0.01	Cerc	0.99	0.01	0.00	Cerc
MCZ 37933	*Procolobus*	*PG*	0.00	0.00	1.00	0.00	*PG*	0.00	0.00	0.99	0.00	*PG*	Cerc	0.98	0.00	0.02	Cerc	0.99	0.00	0.01	Cerc
MCZ 37935	*Procolobus*	*PG*	0.01	0.00	0.99	0.00	*PG*	0.01	0.00	0.99	0.00	*PG*	Cerc	0.88	0.00	0.12	Cerc	0.93	0.00	0.07	Cerc
MCZ 37936	*Procolobus*	*PG*	0.05	0.00	0.95	0.00	*PG*	0.11	0.00	0.89	0.00	*PG*	Cerc	1.00	0.00	0.00	Cerc	1.00	0.00	0.00	Cerc
AMNH 28255	*Nasalis*	*PG*	0.01	0.00	0.99	0.00	*PG*	0.01	0.00	0.99	0.00	*PG*	Cerc	1.00	0.00	0.00	Cerc	1.00	0.00	0.00	Cerc
AMNH 103668	*Nasalis*	*PG*	0.04	0.00	0.96	0.00	*PG*	0.02	0.00	0.98	0.00	*PG*	Cerc	1.00	0.00	0.00	Cerc	1.00	0.00	0.00	Cerc
AMNH 103669	*Nasalis*	*PG*	0.16	0.00	0.84	0.00	*PG*	0.34	0.00	0.66	0.00	*PG*	Cerc	1.00	0.00	0.00	Cerc	1.00	0.00	0.00	Cerc
AMNH 103670	*Nasalis*	*PG*	0.01	0.00	0.99	0.00	*PG*	0.01	0.00	0.99	0.00	*PG*	Cerc	1.00	0.00	0.00	Cerc	0.99	0.00	0.01	Cerc
AMNH 103671	*Nasalis*	*PG*	0.04	0.00	0.96	0.00	*PG*	0.02	0.00	0.98	0.00	*PG*	Cerc	1.00	0.00	0.00	Cerc	1.00	0.00	0.00	Cerc
AMNH 106272	*Nasalis*	*PG*	0.04	0.00	0.96	0.00	*PG*	0.15	0.00	0.85	0.00	*PG*	Cerc	0.99	0.01	0.00	Cerc	1.00	0.00	0.00	Cerc
AMNH 106273	*Nasalis*	*PG*	0.08	0.00	0.92	0.00	*PG*	0.14	0.00	0.86	0.00	*PG*	Cerc	0.99	0.01	0.00	Cerc	0.99	0.01	0.00	Cerc
AMNH 106274	*Nasalis*	*PG*	0.01	0.00	0.99	0.00	*PG*	0.06	0.00	0.94	0.00	*PG*	Cerc	1.00	0.00	0.00	Cerc	1.00	0.00	0.00	Cerc
AMNH 106275	*Nasalis*	*PG*	0.02	0.00	0.98	0.00	*PG*	0.02	0.00	0.98	0.00	*PG*	Cerc	0.98	0.02	0.00	Cerc	0.99	0.01	0.00	Cerc
MCZ 7099	*Nasalis*	*PG*	0.02	0.00	0.97	0.00	*PG*	0.01	0.00	0.99	0.00	*PG*	Cerc	0.99	0.01	0.00	Cerc	0.99	0.01	0.00	Cerc
MCZ 37325	*Nasalis*	*PG*	0.04	0.00	0.96	0.00	*PG*	0.12	0.00	0.88	0.00	*PG*	Cerc	1.00	0.00	0.00	Cerc	1.00	0.00	0.00	Cerc
MCZ 37329	*Nasalis*	*PG*	0.03	0.00	0.96	0.00	*PG*	0.04	0.00	0.96	0.00	*PG*	Cerc	0.99	0.01	0.00	Cerc	1.00	0.00	0.00	Cerc
MCZ 37342	*Nasalis*	*PG*	0.03	0.00	0.97	0.00	*PG*	0.04	0.00	0.96	0.00	*PG*	Cerc	1.00	0.00	0.00	Cerc	1.00	0.00	0.00	Cerc
MCZ 41554	*Nasalis*	*PG*	0.03	0.00	0.97	0.00	*PG*	0.09	0.00	0.91	0.00	*PG*	Cerc	1.00	0.00	0.00	Cerc	1.00	0.00	0.00	Cerc
MCZ 41555	*Nasalis*	*PG*	0.00	0.00	0.99	0.00	*PG*	0.00	0.00	1.00	0.00	*PG*	Cerc	1.00	0.00	0.00	Cerc	1.00	0.00	0.00	Cerc
MCZ 41556	*Nasalis*	*PG*	0.03	0.00	0.97	0.00	*PG*	0.06	0.00	0.94	0.00	*PG*	Cerc	1.00	0.00	0.00	Cerc	1.00	0.00	0.00	Cerc
MCZ 41560	*Nasalis*	*PG*	0.02	0.00	0.98	0.00	*PG*	0.04	0.00	0.96	0.00	*PG*	Cerc	1.00	0.00	0.00	Cerc	1.00	0.00	0.00	Cerc
AMNH 101504	*Trachypithecus*	*PG*	0.01	0.01	0.87	0.11	*PG*	0.01	0.00	0.97	0.02	*PG*	Cerc	0.57	0.43	0.00	Cerc	0.71	0.29	0.00	Cerc
AMNH 102461	*Trachypithecus*	*PG*	0.01	0.00	0.99	0.00	*PG*	0.00	0.00	1.00	0.00	*PG*	Cerc	0.61	0.01	0.38	Cerc	0.83	0.00	0.17	Cerc
AMNH 106598	*Trachypithecus*	*PG*	0.00	0.03	0.80	0.18	*PG*	0.00	0.00	0.34	0.66	***S***	Cerc	0.99	0.01	0.00	Cerc	0.99	0.01	0.00	Cerc

		**a** Positional classification											**b** Taxonomic classification								
			DFA					*glmnet*						DFA				*glmnet*			
Specimen	Taxon	Class	*DG*	*KW*	*PG*	*S*	Pred	*DG*	*KW*	*PG*	*S*	Pred	Class	Cerc	Hom	Plat	Pred	Cerc	Hom	Plat	Pred
MCZ 35636	*Trachypithecus*	*PG*	0.01	0.00	0.98	0.00	*PG*	0.00	0.00	1.00	0.00	*PG*	Cerc	0.60	0.00	0.40	Cerc	0.50	0.00	0.50	**Plat**
MCZ 35640	*Trachypithecus*	*PG*	0.00	0.02	0.72	0.26	*PG*	0.00	0.00	0.43	0.57	***S***	Cerc	0.63	0.02	0.35	Cerc	0.91	0.02	0.07	Cerc
MCZ 35675	*Trachypithecus*	*PG*	0.00	0.00	1.00	0.00	*PG*	0.00	0.00	1.00	0.00	*PG*	Cerc	0.96	0.00	0.04	Cerc	0.98	0.00	0.02	Cerc
MCZ 35682	*Trachypithecus*	*PG*	0.06	0.00	0.94	0.00	*PG*	0.04	0.00	0.96	0.00	*PG*	Cerc	1.00	0.00	0.00	Cerc	1.00	0.00	0.00	Cerc
MCZ 35685	*Trachypithecus*	*PG*	0.00	0.00	1.00	0.00	*PG*	0.00	0.00	1.00	0.00	*PG*	Cerc	0.97	0.01	0.02	Cerc	0.98	0.01	0.01	Cerc
MCZ 37387	*Trachypithecus*	*PG*	0.01	0.07	0.92	0.00	*PG*	0.00	0.00	1.00	0.00	*PG*	Cerc	0.86	0.14	0.00	Cerc	0.88	0.12	0.00	Cerc
MCZ 37391	*Trachypithecus*	*PG*	0.01	0.06	0.93	0.00	*PG*	0.00	0.01	0.99	0.00	*PG*	Cerc	0.99	0.01	0.00	Cerc	0.98	0.02	0.00	Cerc
MCZ 37394	*Trachypithecus*	*PG*	0.01	0.00	0.99	0.00	*PG*	0.00	0.00	1.00	0.00	*PG*	Cerc	0.86	0.00	0.14	Cerc	0.98	0.00	0.02	Cerc
MCZ 37396	*Trachypithecus*	*PG*	0.00	0.03	0.96	0.00	*PG*	0.00	0.00	1.00	0.00	*PG*	Cerc	0.91	0.04	0.04	Cerc	0.95	0.03	0.02	Cerc
MCZ 37399	*Trachypithecus*	*PG*	0.00	0.02	0.98	0.00	*PG*	0.00	0.00	1.00	0.00	*PG*	Cerc	0.99	0.01	0.01	Cerc	0.99	0.01	0.00	Cerc
MCZ 37665	*Trachypithecus*	*PG*	0.01	0.00	0.99	0.00	*PG*	0.00	0.00	1.00	0.00	*PG*	Cerc	0.97	0.00	0.03	Cerc	0.98	0.00	0.02	Cerc
MCZ 37671	*Trachypithecus*	*PG*	0.02	0.02	0.96	0.00	*PG*	0.01	0.00	0.99	0.00	*PG*	Cerc	0.95	0.05	0.00	Cerc	0.95	0.05	0.00	Cerc
AMNH 112976	*Trachypithecus*	*PG*	0.01	0.04	0.95	0.00	*PG*	0.01	0.00	0.99	0.00	*PG*	Cerc	0.91	0.09	0.00	Cerc	0.93	0.07	0.00	Cerc
AMNH 112977	*Trachypithecus*	*PG*	0.00	0.02	0.97	0.00	*PG*	0.00	0.00	0.97	0.03	*PG*	Cerc	0.89	0.11	0.00	Cerc	0.93	0.07	0.00	Cerc
AMNH 106599	*Presbytis*	*PG*	0.00	0.00	0.99	0.00	*PG*	0.00	0.01	0.99	0.00	*PG*	Cerc	0.98	0.00	0.02	Cerc	1.00	0.00	0.00	Cerc
AMNH 106606	*Presbytis*	*PG*	0.03	0.00	0.97	0.00	*PG*	0.04	0.00	0.96	0.00	*PG*	Cerc	0.99	0.00	0.01	Cerc	0.97	0.00	0.03	Cerc
AMNH 211527	*Alouatta*	*PG*	0.02	0.00	0.98	0.00	*PG*	0.01	0.00	0.99	0.00	*PG*	Plat	0.97	0.00	0.03	**Cerc**	0.45	0.00	0.55	Plat
AMNH 211528	*Alouatta*	*PG*	0.13	0.00	0.87	0.00	*PG*	0.04	0.00	0.96	0.00	*PG*	Plat	0.89	0.00	0.11	**Cerc**	0.39	0.00	0.61	Plat
AMNH 211531	*Alouatta*	*PG*	0.06	0.00	0.94	0.00	*PG*	0.11	0.00	0.89	0.00	*PG*	Plat	0.99	0.00	0.01	**Cerc**	0.92	0.00	0.08	**Cerc**
AMNH 211532	*Alouatta*	*PG*	0.00	0.00	1.00	0.00	*PG*	0.00	0.00	1.00	0.00	*PG*	Plat	0.00	0.00	1.00	Plat	0.00	0.00	1.00	Plat
AMNH 211535	*Alouatta*	*PG*	0.00	0.00	1.00	0.00	*PG*	0.00	0.00	1.00	0.00	*PG*	Plat	0.05	0.00	0.95	Plat	0.00	0.00	1.00	Plat
AMNH 211542	*Alouatta*	*PG*	0.00	0.00	1.00	0.00	*PG*	0.00	0.00	1.00	0.00	*PG*	Plat	0.01	0.00	0.99	Plat	0.00	0.00	1.00	Plat
AMNH 211543	*Alouatta*	*PG*	0.00	0.00	1.00	0.00	*PG*	0.00	0.00	1.00	0.00	*PG*	Plat	0.10	0.00	0.90	Plat	0.01	0.00	0.99	Plat
AMNH 211544	*Alouatta*	*PG*	0.00	0.00	1.00	0.00	*PG*	0.00	0.00	1.00	0.00	*PG*	Plat	0.00	0.00	1.00	Plat	0.00	0.00	1.00	Plat
AMNH 23333	*Alouatta*	*PG*	0.03	0.00	0.97	0.00	*PG*	0.00	0.00	1.00	0.00	*PG*	Plat	0.01	0.00	0.99	Plat	0.00	0.00	1.00	Plat
AMNH 23342	*Alouatta*	*PG*	0.01	0.00	0.99	0.00	*PG*	0.00	0.00	1.00	0.00	*PG*	Plat	0.00	0.00	1.00	Plat	0.00	0.00	1.00	Plat
AMNH 187999	*Alouatta*	*PG*	0.01	0.00	0.99	0.00	*PG*	0.00	0.00	1.00	0.00	*PG*	Plat	0.00	0.00	1.00	Plat	0.00	0.00	1.00	Plat
AMNH 188006	*Alouatta*	*PG*	0.01	0.00	0.99	0.00	*PG*	0.00	0.00	1.00	0.00	*PG*	Plat	0.00	0.00	1.00	Plat	0.00	0.00	1.00	Plat
AMNH 30193	*Alouatta*	*PG*	0.00	0.00	1.00	0.00	*PG*	0.00	0.00	1.00	0.00	*PG*	Plat	0.01	0.00	0.99	Plat	0.00	0.00	1.00	Plat
AMNH 42313	*Alouatta*	*PG*	0.00	0.00	1.00	0.00	*PG*	0.00	0.00	1.00	0.00	*PG*	Plat	0.00	0.00	1.00	Plat	0.00	0.00	1.00	Plat
AMNH 42316	*Alouatta*	*PG*	0.00	0.00	1.00	0.00	*PG*	0.00	0.00	1.00	0.00	*PG*	Plat	0.00	0.00	1.00	Plat	0.00	0.00	1.00	Plat
AMNH 132790	*Alouatta*	*PG*	0.00	0.00	1.00	0.00	*PG*	0.00	0.00	1.00	0.00	*PG*	Plat	0.00	0.00	1.00	Plat	0.00	0.00	1.00	Plat

		a Positional classification												b Taxonomic classification									
			DFA					*glmnet*						DFA				*glmnet*			
Specimen	Taxon	Class	*DG*	*KW*	*PG*	*S*	Pred	*DG*	*KW*	*PG*	*S*	Pred	Class	Cerc	Hom	Plat	Pred	Cerc	Hom	Plat	Pred
MCZ 30436	*Alouatta*	*PG*	0.00	0.04	0.96	0.00	*PG*	0.00	0.00	1.00	0.00	*PG*	Plat	0.01	0.00	0.99	Plat	0.09	0.00	0.91	Plat
MCZ 30437	*Alouatta*	*PG*	0.00	0.00	1.00	0.00	*PG*	0.00	0.00	1.00	0.00	*PG*	Plat	0.00	0.00	1.00	Plat	0.00	0.00	1.00	Plat
MCZ 31694	*Alouatta*	*PG*	0.00	0.03	0.97	0.00	*PG*	0.00	0.00	1.00	0.00	*PG*	Plat	0.28	0.01	0.71	Plat	0.33	0.00	0.67	Plat
MCZ 31695	*Alouatta*	*PG*	0.00	0.48	0.47	0.06	***KW***	0.00	0.03	0.89	0.07	*PG*	Plat	0.00	0.00	1.00	Plat	0.00	0.00	0.99	Plat
MCZ 32160	*Alouatta*	*PG*	0.00	0.69	0.31	0.00	***KW***	0.00	0.62	0.38	0.00	***KW***	Plat	0.00	0.01	0.99	Plat	0.04	0.03	0.94	Plat
MCZ 28735	*Alouatta*	*PG*	0.00	0.00	0.99	0.01	*PG*	0.00	0.00	0.88	0.12	*PG*	Plat	0.00	0.00	1.00	Plat	0.00	0.00	1.00	Plat
UMMZ 116300	*Alouatta*	*PG*	0.00	0.00	1.00	0.00	*PG*	0.00	0.00	1.00	0.00	*PG*	Plat	0.01	0.00	0.99	Plat	0.00	0.00	1.00	Plat
UMMZ 116301	*Alouatta*	*PG*	0.00	0.01	0.99	0.00	*PG*	0.00	0.00	1.00	0.00	*PG*	Plat	0.09	0.00	0.91	Plat	0.08	0.00	0.92	Plat
UMMZ 77301	*Alouatta*	*PG*	0.02	0.00	0.98	0.00	*PG*	0.00	0.00	1.00	0.00	*PG*	Plat	0.00	0.00	1.00	Plat	0.01	0.00	0.99	Plat
UMMZ 124689	*Alouatta*	*PG*	0.00	0.00	1.00	0.00	*PG*	0.00	0.00	1.00	0.00	*PG*	Plat	0.00	0.00	1.00	Plat	0.00	0.00	1.00	Plat
UMMZ 124690	*Alouatta*	*PG*	0.00	0.00	1.00	0.00	*PG*	0.00	0.00	1.00	0.00	*PG*	Plat	0.00	0.00	1.00	Plat	0.00	0.00	1.00	Plat
UMMZ 146506	*Alouatta*	*PG*	0.01	0.00	0.99	0.00	*PG*	0.00	0.00	1.00	0.00	*PG*	Plat	0.00	0.00	1.00	Plat	0.00	0.00	1.00	Plat
UMMZ 63503	*Alouatta*	*PG*	0.04	0.00	0.96	0.00	*PG*	0.01	0.00	0.99	0.00	*PG*	Plat	0.01	0.00	0.99	Plat	0.00	0.00	1.00	Plat
UMMZ 63504	*Alouatta*	*PG*	0.02	0.00	0.97	0.00	*PG*	0.00	0.00	1.00	0.00	*PG*	Plat	0.00	0.00	1.00	Plat	0.01	0.00	0.99	Plat
UMMZ 63511	*Alouatta*	*PG*	0.00	0.00	0.99	0.00	*PG*	0.00	0.00	0.97	0.03	*PG*	Plat	0.04	0.00	0.96	Plat	0.07	0.00	0.93	Plat
UMMZ 63512	*Alouatta*	*PG*	0.00	0.00	1.00	0.00	*PG*	0.00	0.00	1.00	0.00	*PG*	Plat	0.01	0.00	0.99	Plat	0.00	0.00	1.00	Plat
AMNH 28418	*Ateles*	*S*	0.00	0.00	0.99	0.00	***PG***	0.00	0.00	1.00	0.00	***PG***	Plat	0.00	0.00	1.00	Plat	0.00	0.00	1.00	Plat
AMNH 28420	*Ateles*	*S*	0.00	0.00	0.72	0.28	***PG***	0.00	0.00	0.05	0.95	*S*	Plat	0.00	0.00	1.00	Plat	0.00	0.00	1.00	Plat
MCZ 34320	*Ateles*	*S*	0.00	0.00	0.00	1.00	*S*	0.00	0.00	0.00	1.00	*S*	Plat	0.00	0.00	1.00	Plat	0.00	0.00	1.00	Plat
MCZ 34322	*Ateles*	*S*	0.00	0.01	0.23	0.76	*S*	0.00	0.00	0.04	0.96	*S*	Plat	0.01	0.00	0.99	Plat	0.01	0.00	0.99	Plat
MCZ 47269	*Ateles*	*S*	0.00	0.00	0.00	1.00	*S*	0.00	0.00	0.00	1.00	*S*	Plat	0.10	0.53	0.37	**Hom**	0.09	0.61	0.30	**Hom**
UMMZ 116302	*Ateles*	*S*	0.00	0.00	0.01	0.99	*S*	0.00	0.00	0.00	1.00	*S*	Plat	0.00	0.00	1.00	Plat	0.00	0.00	1.00	Plat
NMNH 276631	*Ateles*	*S*	0.01	0.00	0.99	0.00	***PG***	0.00	0.00	0.98	0.02	***PG***	Plat	0.02	0.00	0.98	Plat	0.01	0.00	0.99	Plat
NMNH 276657	*Ateles*	*S*	0.00	0.00	0.00	1.00	*S*	0.00	0.00	0.00	1.00	*S*	Plat	0.00	0.05	0.95	Plat	0.00	0.00	1.00	Plat
UMMZ 63165	*Ateles*	*S*	0.00	0.15	0.63	0.21	***PG***	0.00	0.00	0.20	0.80	*S*	Plat	0.08	0.02	0.89	Plat	0.48	0.08	0.44	**Cerc**
UMMZ 63166	*Ateles*	*S*	0.00	0.06	0.21	0.73	*S*	0.00	0.00	0.03	0.97	*S*	Plat	0.00	0.00	1.00	Plat	0.00	0.00	1.00	Plat
UMMZ 63171	*Ateles*	*S*	0.00	0.00	0.00	1.00	*S*	0.00	0.00	0.00	1.00	*S*	Plat	0.00	0.00	1.00	Plat	0.00	0.00	1.00	Plat
NMNH 244863	*Ateles*	*S*	0.00	0.00	0.06	0.94	*S*	0.00	0.00	0.00	1.00	*S*	Plat	0.01	0.00	0.99	Plat	0.07	0.00	0.93	Plat
NMNH 396348	*Ateles*	*S*	0.00	0.01	0.00	0.99	*S*	0.00	0.00	0.00	1.00	*S*	Plat	0.00	0.06	0.94	Plat	0.00	0.19	0.80	Plat
UMMZ 126129	*Cebus*	*PG*	0.00	0.00	0.96	0.04	*PG*	0.00	0.00	0.74	0.26	*PG*	Plat	0.00	0.00	1.00	Plat	0.00	0.00	1.00	Plat
UMMZ 126130	*Cebus*	*PG*	0.00	0.00	0.92	0.08	*PG*	0.00	0.00	0.63	0.37	*PG*	Plat	0.00	0.00	1.00	Plat	0.00	0.00	1.00	Plat
AMNH 133606	*Cebus*	*PG*	0.02	0.00	0.98	0.00	*PG*	0.00	0.00	1.00	0.00	*PG*	Plat	0.00	0.00	1.00	Plat	0.00	0.00	1.00	Plat

		a	Positional classification										b	Taxonomic classification							
			DFA					*glmnet*						DFA				*glmnet*			
Specimen	Taxon	Class	*DG*	*KW*	*PG*	*S*	Pred	*DG*	*KW*	*PG*	*S*	Pred	Class	Cerc	Hom	Plat	Pred	Cerc	Hom	Plat	Pred
AMNH 133607	*Cebus*	*PG*	0.00	0.00	1.00	0.00	*PG*	0.00	0.00	1.00	0.00	*PG*	Plat	0.00	0.00	1.00	Plat	0.00	0.00	1.00	Plat
AMNH 133608	*Cebus*	*PG*	0.00	0.00	1.00	0.00	*PG*	0.00	0.00	1.00	0.00	*PG*	Plat	0.00	0.00	1.00	Plat	0.00	0.00	1.00	Plat
AMNH 133622	*Cebus*	*PG*	0.00	0.03	0.97	0.00	*PG*	0.00	0.00	1.00	0.00	*PG*	Plat	0.00	0.00	1.00	Plat	0.07	0.00	0.93	Plat
AMNH 133624	*Cebus*	*PG*	0.00	0.00	1.00	0.00	*PG*	0.00	0.00	1.00	0.00	*PG*	Plat	0.00	0.00	1.00	Plat	0.00	0.00	1.00	Plat
AMNH 133626	*Cebus*	*PG*	0.00	0.00	0.99	0.00	*PG*	0.00	0.00	1.00	0.00	*PG*	Plat	0.14	0.00	0.86	Plat	0.82	0.01	0.17	**Cerc**
AMNH 133628	*Cebus*	*PG*	0.02	0.00	0.98	0.00	*PG*	0.00	0.00	0.95	0.05	*PG*	Plat	0.00	0.00	1.00	Plat	0.00	0.00	1.00	Plat
AMNH 133629	*Cebus*	*PG*	0.00	0.00	1.00	0.00	*PG*	0.00	0.00	1.00	0.00	*PG*	Plat	0.02	0.00	0.98	Plat	0.03	0.00	0.97	Plat
AMNH 133631	*Cebus*	*PG*	0.00	0.00	1.00	0.00	*PG*	0.00	0.00	1.00	0.00	*PG*	Plat	0.00	0.00	1.00	Plat	0.00	0.00	1.00	Plat
AMNH 133633	*Cebus*	*PG*	0.00	0.00	1.00	0.00	*PG*	0.00	0.00	1.00	0.00	*PG*	Plat	0.00	0.00	1.00	Plat	0.00	0.00	1.00	Plat
AMNH 133635	*Cebus*	*PG*	0.00	0.00	1.00	0.00	*PG*	0.00	0.00	1.00	0.00	*PG*	Plat	0.00	0.00	1.00	Plat	0.00	0.00	1.00	Plat
AMNH 133637	*Cebus*	*PG*	0.00	0.00	1.00	0.00	*PG*	0.00	0.00	1.00	0.00	*PG*	Plat	0.00	0.00	1.00	Plat	0.00	0.00	1.00	Plat
AMNH 133638	*Cebus*	*PG*	0.00	0.00	1.00	0.00	*PG*	0.00	0.00	1.00	0.00	*PG*	Plat	0.01	0.00	0.99	Plat	0.01	0.00	0.99	Plat
AMNH 133640	*Cebus*	*PG*	0.00	0.00	1.00	0.00	*PG*	0.00	0.00	1.00	0.00	*PG*	Plat	0.00	0.00	1.00	Plat	0.00	0.00	1.00	Plat
AMNH 133654	*Cebus*	*PG*	0.00	0.00	1.00	0.00	*PG*	0.00	0.00	1.00	0.00	*PG*	Plat	0.00	0.00	1.00	Plat	0.00	0.00	1.00	Plat
AMNH 133656	*Cebus*	*PG*	0.00	0.01	0.99	0.00	*PG*	0.00	0.00	1.00	0.00	*PG*	Plat	0.00	0.00	1.00	Plat	0.01	0.00	0.99	Plat
AMNH 133660	*Cebus*	*PG*	0.00	0.08	0.92	0.00	*PG*	0.00	0.00	0.95	0.05	*PG*	Plat	0.01	0.00	0.98	Plat	0.16	0.02	0.82	Plat
AMNH 133662	*Cebus*	*PG*	0.00	0.00	1.00	0.00	*PG*	0.00	0.00	1.00	0.00	*PG*	Plat	0.00	0.00	1.00	Plat	0.00	0.00	1.00	Plat
AMNH 133666	*Cebus*	*PG*	0.01	0.00	0.99	0.00	*PG*	0.00	0.00	1.00	0.00	*PG*	Plat	0.02	0.00	0.98	Plat	0.01	0.00	0.99	Plat
AMNH 133667	*Cebus*	*PG*	0.01	0.00	0.99	0.00	*PG*	0.00	0.00	1.00	0.00	*PG*	Plat	0.06	0.00	0.94	Plat	0.16	0.00	0.84	Plat
AMNH 133668	*Cebus*	*PG*	0.01	0.00	0.99	0.00	*PG*	0.00	0.00	1.00	0.00	*PG*	Plat	0.00	0.00	1.00	Plat	0.00	0.00	1.00	Plat
AMNH 133674	*Cebus*	*PG*	0.00	0.00	1.00	0.00	*PG*	0.00	0.00	1.00	0.00	*PG*	Plat	0.00	0.00	1.00	Plat	0.00	0.00	1.00	Plat
AMNH 133677	*Cebus*	*PG*	0.00	0.00	1.00	0.00	*PG*	0.00	0.00	1.00	0.00	*PG*	Plat	0.00	0.00	1.00	Plat	0.00	0.00	1.00	Plat
AMNH 133815	*Cebus*	*PG*	0.01	0.00	0.99	0.00	*PG*	0.00	0.00	1.00	0.00	*PG*	Plat	0.00	0.00	1.00	Plat	0.00	0.00	1.00	Plat
AMNH 133851	*Cebus*	*PG*	0.00	0.00	1.00	0.00	*PG*	0.00	0.00	1.00	0.00	*PG*	Plat	0.00	0.00	1.00	Plat	0.00	0.00	1.00	Plat
AMNH 133862	*Cebus*	*PG*	0.00	0.01	0.97	0.02	*PG*	0.00	0.00	0.98	0.02	*PG*	Plat	0.01	0.00	0.99	Plat	0.16	0.01	0.83	Plat

SI references

1 MacInnes, D. G. Notes on the East African Miocene primates. *The Journal of the East Africa and Uganda Natural History Society* **17**, 141-181, (1943).

2 Pickford, M. & Andrews, P. The Tinderet Miocene sequence in Kenya. *Journal of Human Evolution* **10**, 11-33, (1981).

3 Bishop, W. W., Miller, J. A. & Fitch, F. J. New potassium-argon age determinations relevant to the Miocene fossil mammal sequence in East Africa. *American Journal of Science* **267**, 669-699, (1969).

4 Cote, S., McNulty, K. P., Stevens, N. J. & Nengo, I. O. A detailed assessment of the maxillary morphology of *Limnopithecus evansi* with implications for the taxonomy of the genus. *Journal of Human Evolution* **94**, 83-91, (2016).

5 McNulty, K. P. *et al.* Research on East African catarrhine and hominoid evolution: Results from the first year. *American Journal of Physical Anthropology* **153**, 182-182, (2014).

6 Nengo, I. O. & Rae, T. C. New hominoid fossils from the early Miocene site of Songhor, Kenya. *Journal of Human Evolution* **23**, 423-429, (1992).

7 Cote, S., Malit, N. & Nengo, I. Additional mandibles of *Rangwapithecus gordoni*, an early Miocene catarrhine from the Tinderet localities of Western Kenya. *American Journal of Physical Anthropology* **153**, 341-352, (2014).

8 Gebo, D. L., Malit, N. R. & Nengo, I. O. New proconsuloid postcranials from the early Miocene of Kenya. *Primates* **50**, 311-319, (2009).

9 Bishop, W. W. *The later tertiary in East Africa - Volcanics, sediments, and faunal inventory*. in *Background to Evolution in Africa* (eds W. W. Bishop & J. D. Clark) 31-56 (Chicago University Press, 1967).

10 Harrison, T. & Andrews, P. The anatomy and systematic position of the early Miocene proconsulid from Meswa Bridge, Kenya. *Journal of Human Evolution* **56**, 479-496, (2009).

11 Hill, A., Nengo, I. O. & Rossie, J. B. A *Rangwapithecus gordoni* mandible from the early Miocene site of Songhor, Kenya. *Journal of Human Evolution* **65**, 490-500, (2013).

12 Harrison, T. *Dendropithecoidea, Proconsuloidea, and Hominoidea*. in *Cenozoic Mammals of Africa* (eds L. Werdelin & W. J. Sanders) 429-469 (University of California Press, 2010).

13 Harrison, T. New finds of small fossil apes from the Miocene locality at Koru in Kenya. *Journal of Human Evolution* **10**, 129-137, (1981).

14 Harrison, T. A taxonomic revision of the small catarrhine primates from the Early Miocene of East Africa. *Folia Primatologica* **50**, 59-108, (1988).

15 Kay, R. F. & Ungar, P. S. *Dental evidence for diet in some Miocene catarrhines with comments on the effects of phylogeny on the interpretation of adaptation*. in *Function, Phylogeny, and Fossils* (eds D. R. Begun, C. V. Ward, & M. D. Rose) 131-151 (Plenum Press, 1997).

16 Shearer, B. M. *et al.* Dental microwear profilometry of African non-cercopithecoid catarrhines of the Early Miocene. *Journal of Human Evolution* **78**, 33-43, (2015).

17 McNulty, K. P. *et al.* New partial cranium from an early Miocene locality at Lower Kapurtay. *American Journal of Physical Anthropology* **156**, 222-222, (2015).

18 Jansma, R. J. W. & MacLatchy, L. M. First evidence of Nyanzapithecinae at Moroto II, Uganda. *American Journal of Physical Anthropology* **156**, 177-177, (2015).

19 Schmitt, D. *Forelimb mechanics during arboreal and terrestrial quadrupedalism in Old World monkeys*. in *Primate Locomotion: Recent Advances* (eds E. Strasser, J. Fleagle, A. Rosenberger, & H. McHenry) 175-200 (Springer Science+Business Media, LLC, 1998).

20 Patel, B. A. Functional morphology of cercopithecoid primate metacarpals. *Journal of Human Evolution* **58**, 320-337, (2010).

21 Patel, B. A. & Wunderlich, R. E. Dynamic pressure patterns in the hands of olive baboons (*Papio anubis*) during terrestrial locomotion: implications for cercopithecoid primate hand morphology. *Anat Rec* **293**, 710-718, (2010).

22 Polk, J. D., Williams, S. A., Peterson, J. V., Roseman, C. C. & Godfrey, L. R. Subchondral bone apparent density and locomotor behavior in extant primates and subfossil lemurs *Hadropithecus* and *Pachylemur*. *International Journal of Primatology* **31**, 275-299, (2010).

23 Begun, D. R. & Kivell, T. L. Knuckle-walking in *Sivapithecus*? The combined effects of homology and homoplasy with possible implications for pongine dispersals. *Journal of Human Evolution* **60**, 158-170, (2011).

24 Fernandez, P. J. *et al.* Functional aspects of metatarsal head shape in humans, apes, and Old World monkeys. *Journal of Human Evolution* **86**, 136-146, (2015).

25 Lewton, K. L. Pelvic form and locomotor adaptation in strepsirrhine primates. *Anat. Rec.* **298**, 230-248, (2015).

26 Selby, M. S., Simpson, S. W. & Lovejoy, C. O. The functional anatomy of the carpometacarpal complex in anthropoids and its implications for the evolution of the hominoid hand. *Anat. Rec.* **299**, 583-600, (2016).

27 Selby, M. S. & Lovejoy, C. O. Evolution of the hominoid scapula and its implications for earliest hominid locomotion. *American Journal of Physical Anthropology* **162**, 682-700, (2017).

28 Orr, C. M. Locomotor hand postures, carpal kinematics during wrist extension, and associated morphology in anthropoid primates. *Anat. Rec.* **300**, 382-401, (2017).

29 Hunt, K. D. *et al.* Standardized descriptions of primate locomotion and postural modes. *Primates* **37**, 363-387, (1996).

30 Walker, S. E. *Fine-grained differences within positional categories: a case study of Pithecia and Chiropotes*. in *Primate Locomotion: Recent Advances* (eds E. Strasser, J. Fleagle, A. Rosenberger, & H. McHenry) 31-43 (Springer Science+Business Media, LLC, 1998).

31 Patel, B. A. Not so fast: speed effects on forelimb kinematics in cercopithecine monkeys and implications for digitigrade postures in primates. *American Journal of Physical Anthropology* **140**, 92-112, (2009).

32 Patel, B. A. The interplay between speed, kinetics, and hand postures during primate terrestrial locomotion. *American Journal of Physical Anthropology* **141**, 222-234, (2010).
33 Patel, B. A., Larson, S. G. & Stern, J. T., Jr. Electromyography of wrist and finger flexor muscles in olive baboons (Papio anubis). *Journal of Experimental Biology* **215**, 115-123, (2012).
34 Wuthrich, C., MacLatchy, L. M., McCrossin, M. L. & Benefit, B. R. Can digitigrade hand postures be inferred from carpal morphology in extant and fossil cercopithecoids? *American Journal of Physical Anthropology* **165**, 308, (2018).
35 Wuthrich, C. *Computational Relationships among Form, Function, and Phylogeny in the Catarrhine Ulnar Carpus, and the Evolutionary History of Ape and Human Locomotion* Ph.D. Dissertation, University of Michigan, (2017).
36 Hunt, K. D. Positional behavior in the Hominoidea. *International Journal of Primatology* **12**, 95-18, (1991).
37 Sarringhaus, L. A., MacLatchy, L. M. & Mitani, J. C. Long bone cross-sectional properties reflect changes in locomotor behavior in developing chimpanzees. *American Journal of Physical Anthropology* **160**, 16-29, (2016).
38 Vedder, A. L. Movement patterns of a group of free-ranging mountain gorillas (*Gorilla gorilla beringei*) and their relation to food availability. *Am J Primatol* **7**, 73-88, (1984).
39 Tuttle, R. H. & Watts, D. P. *The positional behavior and adaptive complexes of Pan gorilla*. in *Primate Morphophysiology, Locomotor Analyses and Human Bipedalism* (ed S. Kondo) 261-288 (University of Tokyo Press, 1985).
40 Remis, M. J. Ranging and grouping patterns of a western lowland gorilla group at Bai Hokou, Central African Republic. *Am J Primatol* **43**, 111-133, (1997).
41 Remis, M. J. *The gorilla paradox*. in *Primate Locomotion* (ed E. Strasser) 95-106 (Plenum Press, 1998).
42 Slizewski, A., Friess, M. & Semal, P. Surface scanning of anthropological specimens: nominal-actual comparison with low cost laser scanner and high end fringe light projection surface scanning systems. *Quartar* **57**, 179-187, (2010).
43 Tocheri, M. W. *et al.* Ecological divergence and medial cuneiform morphology in gorillas. *Journal of Human Evolution* **60**, 171-184, (2011).
44 Polo, M. E. & Felicisimo, A. M. Analysis of uncertainty and repeatability of a low-cost 3D laser scanner. *Sensors* **12**, 9046-9054, (2012).
45 Patel, B. A., Yapuncich, G. S., Tran, C. & Nengo, I. O. Catarrhine hallucal metatarsals from the early Miocene site of Songhor, Kenya. *Journal of Human Evolution* **108**, 176-198, (2017).
46 Kivell, T. L. *et al.* New Neandertal wrist bones from El Sidrón, Spain (1994–2009). *Journal of Human Evolution* **114**, 45-75, (2018).
47 Ibáñez-Gimeno, P. *et al.* Forearm pronation efficiency in A.L. 288-1 (Australopithecus afarensis) and MH2 (Australopithecus sediba): Insights into their locomotor and manipulative habits. *American Journal of Physical Anthropology* **164**, 788-800, (2017).
48 Püschel, T. A., Gladman, J. T., Bobe, R. & Sellers, W. I. The evolution of the platyrrhine talus: A comparative analysis of the phenetic affinities of the Miocene platyrrhines with their modern relatives. *Journal of Human Evolution* **111**, 179-201, (2017).
49 Frelat, M. A. *et al.* Evolution of the hominin knee and ankle. *Journal of Human Evolution* **108**, 147-160, (2017).
50 Fernández, P. J. *et al.* Evolution and function of the hominin forefoot. *Proceedings of the National Academy of Sciences* **115**, 8746-8751, (2018).
51 Doran, D. M. Sex differences in adult chimpanzee positional behavior: The influence of body size on locomotion and posture. *American Journal of Physical Anthropology* **91**, 99-115, (1993).
52 Carlson, K. J. Investigating the form-function interface in African apes: Relationships between principal moments of area and positional behaviors in femoral and humeral diaphyses. *American Journal of Physical Anthropology* **127**, 312-334, (2005).
53 Doran, D. M. & Hunt, K. D. *Comparative locomotor behavior of chimpanzees and bonobos*. in *Chimpanzee Cultures* (eds R. Wrangham, W. C. McGrew, Frans B. M. De Waal, & P. Heltne) 93-108 (Harvard University Press, 1994).
54 Remis, M. J. *Feeding Ecology and Positional Behavior of Western Lowland Gorillas (Gorilla gorilla gorilla) in the Central African Republic.* Ph.D. Dissertation, Yale University, (1994).
55 Doran, D. M. *Comparative positional behavior of the African apes*. in *Great Ape Societies* (eds W. McGrew, L. Marchant, & T. Nishida) 213-224 (Cambridge University Press, 1996).
56 Doran, D. M. Ontogeny of locomotion in mountain gorillas and chimpanzees. *Journal of Human Evolution* **32**, 323-344, (1997).
57 Cant, J. G. H. Positional behavior of female Bornean orangutans (*Pongo pygmaeus*). *Am J Primatol* **12**, 71-90, (1987).
58 Thorpe, S. K. & Crompton, R. H. Orangutan positional behavior and the nature of arboreal locomotion in Hominoidea. *American Journal of Physical Anthropology* **131**, 384-401, (2006).
59 Sati, J. P. & Alfred, J. R. B. Locomotion and posture in hoolock gibbon. *Annals of Forestry* **10**, 298-306, (2002).
60 Nowak, M. G. & Reichard, U. H. *The torso-orthograde positional behavior of wild white-handed gibbons (Hylobates lar)*. in *Evolution of Gibbons and Siamang* (eds U. H. Reichard, H. Hirai, & C. Barelli) 203-225 (Springer, 2016).
61 Hunt, K., D. *The special demands of great ape locomotion and posture*. in *The Evolution of Thought* (eds Anne E. Russon & David R. Begun) 172-189 (Cambridge University Press, 2004).
62 Gebo, D. L. & Chapman, C. A. Positional behavior in five sympatric Old World monkeys. *American Journal of Physical Anthropology* **97**, 49-76, (1995).
63 Aronsen, G. P. *Positional behavior and support use in three arboreal monkeys of the Kibale forest, Uganda: the influences of forest structure, microhabitats, and energetics.* Ph.D. Dissertation, Yale University, (2004).
64 Ankel-Simons, F. *Primate Anatomy: An Introduction*. 3 edn, (Elsevier, 2007).
65 Rollinson, J. & Martin, R. D. *Comparative Aspects of Primate Locomotion, with special reference to arboreal cercopithecines*. in *Vertebrate Locomotion* (ed M. H. Day) 377-427 (Academic Press, 1981).
66 Cant, J. G. H. Positional behavior of long-tailed macaques (*Macaca fascicularis*) in northern Sumatra. *American Journal of Physical Anthropology* **76**, 29-37, (1988).

67 Isbell, L. A., Pruetz, J. D., Lewis, M. & Young, T. P. Locomotor activity differences between sympatric patas monkeys (*Erythrocebus patas*) and vervet monkeys (*Cercopithecus aethiops*): implications for the evolution of long hindlimb length in *Homo*. *American Journal of Physical Anthropology* **105**, 199-207, (1998).
68 Kern, J. A. Observations on the habits of the proboscis monkey, *Nasalis larvatus* (Wurmb), made in the Brunei Bay area, Borneo. *Zoologica* **49**, 183-192, (1964).
69 Kawabe, M. & Mano, T. Ecology and behavior of the wild proboscis monkey, *Nasalis larvatus* (Wurmb), in Sabeh, Malaysia. *Primates* **13**, 213-228, (1972).
70 Bennett, E. & Sebastian, A. Social Organization and Ecology of Proboscis Monkeys (Nasalis larvatus) in Mixed Coastal Forest in Sarawak. *International Journal of Primatology* **9**, 233-255, (1988).
71 Fleagle, J. G. *Locomotion and posture*. in *Malayan Forest Primates* (ed D. J. Chivers) 191-207 (Plenum Press, 1980).
72 Youlatos, D. & Guillot, D. *Howler monkey positional behavior*. in *Howler Monkeys* (eds M. M. Kowalewski *et al.*) 191-218 (Springer, 2015).
73 Mittermeier, R. A. Locomotion and posture in *Ateles geoffroyi* and *Ateles paniscus*. *Folia Primatologica* **30**, 161-193, (1978).
74 Cant, J. G. H. Locomotion and feeding postures of spider and howling monkeys: field study and evolutionary interpretation. *Folia Primatologica* **46**, 1-14, (1986).
75 Fontaine, R. Positional behavior in *Saimiri boliviensis* and *Ateles geoffroyi*. *American Journal of Physical Anthropology* **82**, 485-508, (1990).
76 Wright, K. A. The relationship between locomotor behavior and limb morphology in brown (*Cebus apella*) and weeper (*Cebus olivaceus*) capuchins. *Am J Primatol* **69**, 736-756, (2007).
77 Youlatos, D. Positional behavior of two sympatric Guianan capuchin monkeys, the brown capuchin (*Cebus apella*) and the wedge-capped capuchin *(Cebus olivaceus*). *Mammalia* **62**, 351-365, (1998).
78 Doran, D. M. Comparative locomotor behavior of chimpanzees and bonobos: the influence of morphology on locomotion. *American Journal of Physical Anthropology* **91**, 83-98, (1993).
79 Galdikas, B. M. F. *Orangutan Adaptation at Tanjung Puting Reserve, Central Borneo.* Ph.D. Dissertation, UCLA, (1978).
80 Wheatley, B. P. Energetics of foraging in *Macaca fascicularis* and *Pongo pygrnaeus* and a selective advantage of large body size in the orang-utan. *Primates* **23**, 348-363, (1982).
81 Ruff, C. B. Long bone articular and diaphyseal structure in old world monkeys and apes. I: locomotor effects. *American Journal of Physical Anthropology* **119**, 305-342, (2002).
82 Ruff, C. B., Burgess, M. L., Squyres, N., Junno, J.-A. & Trinkaus, E. Lower limb articular scaling and body mass estimation in Pliocene and Pleistocene hominins. *Journal of Human Evolution*, (2018).
83 Perry, J. M. G., Cooke, S. B., Runestad Connour, J. A., Burgess, M. L. & Ruff, C. B. Articular scaling and body mass estimation in platyrrhines and catarrhines: Modern variation and application to fossil anthropoids. *Journal of Human Evolution* **115**, 20-35, (2018).
84 Tsubamoto, T., Egi, N., Takai, M., Thaung, H. & Zin Maung Maung, T. Body mass estimation from the talus in primates and its application to the Pondaung fossil amphipithecid primates. *Historical Biology* **28**, 27-34, (2015).
85 Rafferty, K. L., Walker, A., Ruff, C. B., Rose, M. D. & Andrews, P. J. Postcranial estimates of body weight in *Proconsul*, with a note on a distal tibia of *P. major* from Napak, Uganda. *American Journal of Physical Anthropology* **97**, 391-402, (1995).
86 Ruff, C. B. Long bone articular and diaphyseal structure in Old World monkeys and apes. II: Estimation of body mass. *American Journal of Physical Anthropology* **120**, 16-37, (2003).
87 Smith, R. J. & Jungers, W. L. Body mass in comparative primatology. *Journal of Human Evolution* **32**, 523-559, (1997).
88 Delson, E. *et al.* Body mass in Cercopithecidae (Primates, Mammalia): Estimation and scaling in extinct and extant taxa. *American Museum of Natural History, Anthropological Papers* **83**, (2000).
89 Smith, R. J. Allometric scaling in comparative biology: problems of concept and method. *American Journal of Physiology* **246**, R152-R160, (1984).
90 Smith, R. J. *The present as a key to the past: body weight of Miocene hominoids as a test of allometric methods for paleontological inference*. in *Size and Scaling in Primate Biology* (ed W. L. Jungers) 437-448 (Plenum Press, 1985).
91 Konigsberg, L. W., Hens, S. M., Jantz, L. M. & Jungers, W. L. Stature estimation and calibration: Bayesian and maximum likelihood perspectives in physical anthropology. *Yearbook of Physical Anthropology* **41**, 65-92, (1998).
92 Uhl, N. M., Rainwater, C. W. & Konigsberg, L. W. Testing for size and allometric differences in fossil hominin body mass estimation. *American Journal of Physical Anthropology* **151**, 215-229, (2013).
93 Packard, G. C. Fitting statistical models in bivariate allometry: scaling metabolic rate to body mass in mustelid carnivores. *Comparative Biochemistry and Physiology Part A: Molecular & Integrative Physiology* **166**, 70-73, (2013).
94 Packard, G. C. Allometric variation in the antlers of cervids: a comment on Lemaitre et al. *Biol Lett* **11**, 20140923, (2015).
95 Lemaitre, J. F., Vanpe, C., Plard, F., Pelabon, C. & Gaillard, J. M. Response to Packard: make sure we do not throw out the biological baby with the statistical bath water when performing allometric analyses. *Biol Lett* **11**, 20150144, (2015).
96 Sprugel, D. G. Correcting for bias in log-transformed allometric equations. *Ecology* **64**, 209-210, (1983).
97 Smith, R. J. Logarithmic transformation bias in allometry. *American Journal of Physical Anthropology* **90**, 215-228, (1993).
98 Miller, A. J. *Subset Selection in Regression*. 2nd edn, (CRC Press, 2002).
99 Ritter, G. *Robust Cluster Analysis and Variable Selection*. (CRC Press, 2015).
100 Barr, W. A. & Scott, R. S. Phylogenetic comparative methods complement discriminant function analysis in ecomorphology. *American Journal of Physical Anthropology* **153**, 663-674, (2014).
101 Grafen, A. The phylogenetic regression. *Philosophical Transactions of the Royal Society of London B* **326**, 119-157, (1989).
102 Hadfield, J. D. & Nakagawa, S. General quantitative genetic methods for comparative biology: phylogenies, taxonomies and multi-trait models for continuous and categorical characters. *Journal of evolutionary biology* **23**, 494-508, (2010).

103 Hadfield, J. D. MCMC methods for multi-response generalized linear mixed models: The MCMCglmm R package. *Journal of Statistical Software* **33**, 1-22, (2010).
104 Carter, K. E. & Worthington, S. The evolution of anthropoid molar proportions. *BMC evolutionary biology* **16**, 110, (2016).
105 Gelman, A. Prior distributions for variance parameters in hierarchical models (comment on article by Browne and Draper). *Bayesian Analysis* **1**, 515-534, (2006).
106 Worthington, S. *New approaches to late Miocene hominoid systematics: ranking morphological characters by phylogenetic signal* PhD, New York University, (2012).
107 Pagel, M. Inferring the historical patterns of biological evolution. *Nature* **401**, 877-884, (1999).
108 Blomberg, S. P., Garland, T., Jr. & Ives, A. R. Testing for phylogenetic signal in comparative data: behavioral traits are more labile. *Evolution* **57**, 717-745, (2003).
109 Felsenstein, J. Phylogenies and the comparative method. *The American Naturalist* **125**, 1-15, (1985).
110 Arnold, C., Matthews, L. J. & Nunn, C. L. The 10kTrees website: A new online resource for primate phylogeny. *Evolutionary Anthropology* **19**, 114-118, (2010).
111 Gascuel, O. BIONJ: An improved version of the NJ algorithm based on a simple model of sequence data. *Molecular Biology and Evolution* **14**, 685-695, (1997).
112 Saitou, N. & Nei, M. The neighbor-joining method: a new method for reconstructing phylogenetic trees. *Molecular Biology and Evolution* **4**, 406-425, (1987).
113 Studier, J. A. & Keppler, K. J. A note on the neighbor-joining algorithm of Saitou and Nei. *Molecular Biology and Evolution* **5**, 729-731, (1988).
114 Felsenstein, J. *Distance matrix methods*. in *Inferring Phylogenies* (ed J. Felsenstein) 147-175 (Sinauer Associates, Inc., 2004).
115 Gascuel, O. On the optimization principle in phylogenetic analysis and the minimum-evolution criterion. *Molecular Biology and Evolution* **17**, 401-405, (2000).
116 R: A language and environment for statistical computing (R Foundation for Statistical Computing, Vienna, Austria, 2018).
117 caper: comparative analyses of phylogenetics and evolution in R. R package version 0.5.2. (2013).
118 Morpho: Calculations and visualisations related to geometric morphometrics. R package version 2.4.1.1. (2016).
119 Adams, D. C. & Otárola-Castillo, E. geomorph: an R package for the collection and analysis of geometric morphometric shape data. *Methods in Ecology and Evolution* **4**, 393-399, (2013).
120 Venables, W. N. & Ripley, B. D. *Modern Applied Statistics with S. 4th edition*. (Springer, 2002).
121 ipred: Improved Predictors. R package version 0.9-5. (2015).
122 MuMIn: Multi-Model Inference. R package version 1.15.6. (2016).
123 Friedman, J., Hastie, T. & Tibshirani, R. Regularization paths for generalized linear models via coordinate descent. *Journal of Statistical Software* **33**, 1-22, (2010).
124 caret: Classification and Regression Training. R package version 6.0-73. (2016).
125 MASSTIMATE: Body Mass Estimation Equations for Vertebrates. R package version 1.3. (2016).
126 Hmisc: Harrell Miscellaneous. R package version 4.0-0. (2016).
127 Hothorn, T., Bretz, F. & Westfall, P. Simultaneous inference in general parametric models. *Biometrical Journal* **50**, 346-363, (2008).
128 Paradis, E., Claude, J. & Strimmer, K. APE: analyses of phylogenetics and evolution in R language. *Bioinformatics* **20**, 289-290, (2004).
129 Chen, W.-C. *Overlapping Codon model, Phylogenetic Clustering, and Alternative Partial Expectation Conditional Maximization Algorithm* Ph.D. Dissertation, Iowa State University, (2011).
130 Wickham, H. *Elegant Graphics for Data Analysis*. (Springer-Verlag, 2009).
131 Beard, K. C., Teaford, M. F. & Walker, A. New wrist bones of *Proconsul africanus* and *P. nyanzae* from Rusinga Island, Kenya. *Folia Primatologica* **47**, 97-118, (1986).
132 Jenkins Jr., F. A. Wrist rotation in primates: A critical adaptation for brachiators. *Symposia of the Zoological Society of London* **48**, 429-451, (1981).
133 Rose, M. D. Hominoid postcranial specimens from the middle Miocene Chinji formation, Pakistan. *Journal of Human Evolution* **13**, 503-516, (1984).
134 Ogihara, N. *et al.* Carpal bones of *Nacholapithecus kerioi,* a middle Miocene hominoid from northern Kenya. *American Journal of Physical Anthropology* **160**, 469-482, (2016).
135 Byron, C. D. & Covert, H. H. Unexpected locomotor behaviour: brachiation by an Old World monkey (*Pygathrix nemaeus*) from Vietnam. *Journal of Zoology* **263**, 101-106, (2004).
136 Byron, C. D., Hensel, C., Morrison, J. & Nguyen, H. The skeletal anatomy of the douc langurs (Genus *Pygathrix*). *Vietnamese Journal of Primatology* **2**, 13-24, (2015).
